# High C1QTNF1 expression mediated by potential ncRNAs is associated with poor prognosis and tumor immunity in kidney renal clear cell carcinoma

**DOI:** 10.3389/fmolb.2023.1201155

**Published:** 2023-07-17

**Authors:** Jiechuan Qiu, Zicheng Wang, Leizuo Zhao, Peizhi Zhang, Yingkun Xu, Qinghua Xia

**Affiliations:** ^1^ Department of Urology, Shandong Provincial Hospital Affiliated to Shandong First Medical University, Jinan, China; ^2^ Department of Urology, Dongying People’s Hospital, Dongying, China; ^3^ Department of Urology, Shandong Provincial Hospital, Cheeloo College of Medicine, Shandong University, Jinan, China; ^4^ Department of Breast and Thyroid Surgery, The First Affiliated Hospital of Chongqing Medical University, Chongqing, China

**Keywords:** clear cell renal cell carcinoma, C1QTNF1, ceRNA, prognostic markers, adipokines

## Abstract

**Background:** Kidney renal clear cell carcinoma (KIRC) originates from proximal tubular cells and is the most common subtype of renal cell carcinoma. KIRC is characterized by changes in lipid metabolism, and obesity is a risk factor for it. C1q And TNF Related 1 (C1QTNF1), a novel adipokine and member of the C1q and TNF-related protein (CTRP) family, has been shown to affect the progression of various cancers. However, the role of C1QTNF1 in KIRC has not been studied.

**Methods:** The Wilcoxon rank sum test was used to analyze the expression of C1QTNF1 in KIRC tissues and normal tissues. The relationship between clinicopathological features and C1QTNF1 levels was also examined by logistic regression and the Wilcoxon rank sum test. In addition, the effect of C1QTNF1 on the prognosis of KIRC patients was analyzed by Kaplan-Meier (KM). The Gene Ontology (GO) and Kyoto Encyclopedia of Genes and Genomes (KEGG) were used to analyze the potential signaling pathways and biological functions of differential genes. A nomogram was constructed to predict the prognosis of KIRC patients. Spearman correlation analysis was performed to determine the association between C1QTNF1 expression and immune cell infiltration and immune checkpoint genes. The upstream miRNAs and lncRNAs of C1QTNF1 were predicted by the ENCORI online tool. Finally, we examined the proliferation, invasion, and migration abilities of KIRC cells after C1QTNF1 knockdown.

**Results:** The expression of C1QTNF1 in KIRC tissues was significantly higher than in normal renal tissues. Patients with higher C1QTNF1 expression had a poor prognosis, a finding supported by Kaplan-Meier survival analysis. C1QTNF1 expression was significantly correlated with TNM and pathologic stages, age, and gender (*p* < 0.05). The C1QTNF1 expression level was significantly correlated with immune cell infiltration and immune checkpoint genes in KIRC. Additionally, high C1QTNF1 expression was associated with poor prognosis in stage I and II, T1 and T2, T3 and T4, N0, and M0 patients (HR > 1, *p* < 0.05). The calibration diagram shows that the C1QTNF1 model has effective predictive performance for the survival of KIRC patients. Knockdown of C1QTNF1 inhibited KIRC cell proliferation, cell migration, and cell invasion. In addition, CYTOR and AC040970.1/hsa-miR-27b-3p axis were identified as the most likely upstream ncRNA-related pathways of C1QTNF1 in KIRC.

**Conclusion:** In conclusion, our study suggests that high expression of C1QTNF1 is associated with KIRC progression and immune infiltration. The increased expression of C1QTNF1 suggests a poor prognosis in KIRC patients.

## 1 Introduction

Renal cell carcinoma (RCC) is one of the most common urologic tumors. Renal clear cell carcinoma is the most common histological subtype of renal cell carcinoma (Hsieh et al., 2017). Due to the poor sensitivity of renal clear cell carcinoma to radiotherapy and chemotherapy, surgical resection is still the main treatment for KIRC (Van Poppel et al., 2011). Although targeted therapy and immune checkpoint inhibitors have made outstanding contributions to the treatment of KIRC in the past period, due to the emergence of drug resistance of tumor cells, the treatment effect of patients in the later stage is often reduced (Buczek et al., 2014; Ballesteros et al., 2021). Therefore, it is urgent to find new diagnostic and therapeutic targets for the treatment of KIRC.

The number of obesity-related diseases has been increasing over the past few decades (Seidell and Halberstadt, 2015). There is evidence that obesity promotes an increased incidence of several cancers (Lauby-Secretan et al., 2016). It has been previously shown that obesity is a risk factor for renal clear cell renal cell carcinoma (Sanchez and Simon, 2018) and that perirenal fat infiltration leads to poor clinical outcomes (da Costa et al., 2012). As obesity leads to an increase in circulating pro-inflammatory adipokines, these pro-inflammatory adipokines can lead to a variety of biological processes including tumorigenesis. A study has identified an important role for the obesity-related adipokine Chemerin in KIRC cells (Tan et al., 2021). C1QTNF1 is a member of the CTRP family, a novel adipokine (Schäffler and Buechler, 2012). Recently, it has been found that the level of C1QTNF1 is increased in obese mice compared with normal mice, and C1QTNF1 expression or secreted C1QTNF1 activate cancer cell proliferation through a p53-dependent pathway (Park et al., 2021). We hypothesize that C1QTNF1 as an adipokine may also play a deleterious role in driving KIRC tumor progression.

In this study, we used The Cancer Genome Atlas (TCGA, https://portal.gdc.cancer.gov/), Genotype-Tissue Expression (GTEx, https://www.gtexportal.org) datasets, and Kaplan-Meier (KM) plotters (https://kmplot.com/analysis) to analyze C1QTNF1 expression and its association with clinical significance. The function and pathway enrichment of C1QTNF1-related differential genes were analyzed to explore the potential mechanism of C1QTNF1 regulation involved in KIRC progression. Meanwhile, the relationship between C1QTNF1 and immune infiltration was explored in KIRC. In addition, a knockdown of C1QTNF1 was performed to determine its effect on KIRC. Through a series of correlation, expression, and survival analyses, we identified CYTOR and the AC040970.1/hsa-miR-27b-3p axis as the most likely upstream ncRNA-related pathways of C1QTNF1 in KIRC.

## 2 Materials and methods

### 2.1 Acquisition of data

Raw RNA sequencing data from 33 different cancer tissues and normal tissues were obtained from the TCGA and GTEx databases for pan-cancer analysis. The TCGA-KIRC dataset’s corresponding clinical data was also downloaded. The raw data was acquired in HTSeq-FPKM format, and converted to TPM format, followed by log2 transformation.

### 2.2 Pathological tissue harvesting

In this study, 16 pairs of KIRC tumor tissues and paired normal tissues (derived from patients who underwent surgery in Shandong Provincial Hospital from 2019 to 2021) were collected for quantitative polymerase chain reaction (qPCR) and immunohistochemistry (IHC) to verify the expression level of C1QTNF1. All the patients were aware of the intent of the study and provided written informed consent. This study followed the Declaration of Helsinki and was approved by the Hospital Medical Ethics Committee of Shandong Provincial Hospital Affiliated to Shandong First Medical University.

### 2.3 Screening of differentially expressed genes

KIRC patients were separated into C1QTNF1 high expression and low expression groups based on the median C1QTNF1 expression in the TCGA-KIRC dataset. Differential analysis between the high and low expression groups was conducted using the R package “DESeq2”. A threshold of *p* values <0.05 and |logFC| > 1.5 was set. The Spearman correlation coefficient method was employed to analyze the correlation between C1QTNF1 and the top 10 differentially expressed genes (DEGs). The results were presented using volcano plots and heat maps created using the R packages “ggplot2” and “pheatmap”.

### 2.4 Immune infiltration analysis

The relationship between C1QTNF1 expression in KIRC samples and the abundance of 24 immune cells in tumors was evaluated using the ssGSEA algorithm in the “GSVA” R package, including neutrophils, cytotoxic cells, dendritic cells (DCs), CD8^+^ T-cells, plasmacytoid DC (pDC), natural killer (NK) cells, mast cells, T gamma delta (Tgd), type 17 Th (Th17) cells, immature DCs (iDCs), eosinophils, NK CD56dim cells, regulatory T-cells (TReg), T effector memory (Tem), T-cells, T central memory (Tcm), B cells, type 1 Th (Th1) cells, macrophages, NK CD56bright cells, activated DC (aDC), T follicular helper (TFH), T helper cells, and type 2 Th (Th2) cells. Correlation analysis between C1QTNF1 and immune checkpoints was conducted *via* the “ggplot2” R package and Spearman correlation coefficient. When *p* < 0.05, the correlation was significant. TISIDB (http://cis.hku.hk/TISIDB/) is a portal containing a variety of tumor immunology data resources, which can be used to explore the relationship between genes and immunological features (Ru et al., 2019). We used TISIDB to determine the association of C1QTNF1 with TIL infiltration as well as immune checkpoints.

### 2.5 Prognostic significance of C1QTNF1 expression

The GEPIA2 database (http://gepia2.cancer-pku.cn/#index) is a free and open online database that can be used by users to determine the expression level and prognostic impact of target molecules in different tumors (Tang et al., 2019). The Kaplan-Meier Plotter online site assesses the relationship between genes and survival outcomes in a variety of cancers, and users can look for the prognostic value of specific genes analyzed (Lánczky and Győrffy, 2021). The GEPIA2 database was used to analyze the prognostic significance of C1QTNF1 expression in pan-cancer. For the TCGA-KIRC dataset, prognostic analysis was performed using R packages “survival” and “survminer”. KIRC patients were divided into a C1QTNF1 high expression group and a low expression group according to the median value of C1QTNF1 expression. The prognostic value of the C1QTNF1 expression level in KIRC was evaluated by the Kaplan-Meier curve and log-rank test. The Kaplan-Meier Plotter was used to verify the relationship between C1QTNF1 expression level and the survival outcome of KIRC patients. Based on Cox regression analysis, the independent risk factors affecting the prognosis of KIRC were analyzed, and then the factors obtained by multivariate Cox regression were included in the prognostic nomogram to evaluate the prognosis of KIRC patients. The discrimination of the nomogram was then quantified using the concordance index (C-index), and the performance of the nomogram was evaluated using a calibration plot. The R package “rms” was used to draw the nomogram and calibration plots. “pROC” and “timeROC” were used to draw the diagnostic ROC curve and the time-dependent survival ROC curve. The prognosis analysis results of KIRC subgroups were visualized by the R package “ggplot2.”

### 2.6 C1QTNF1-related genes, interacting chemicals, and candidate miRNA and lncRNA predictions

GeneMENIA (http://www.genemania.org) is a flexible, user-friendly online tool that allows users to upload their own data sets to predict the priority and function of genes (Warde-Farley et al., 2010). Genes co-expressed with C1QTNF1 were predicted by the GeneMANIA online tool. The Comparative Toxicogenomic Database (CTD, http://ctdbase.org/) is a friendly, innovative database containing toxicological information on chemicals, genes, phenotypes, diseases, and exposures that provides potential molecular mediators to develop testable hypotheses (Davis et al., 2023). CTD was used to predict the chemicals interacting with C1QTNF1. ENCORI (http://starbase.sysu.edu.cn/) is a comprehensive online database that allows users to explore the miRNA-mRNA and miRNA-lncRNA interaction networks and predict the function of miRNAs and other ncRNAs (Li et al., 2014). The authors predicted the upstream potential miRNAs and lncRNAs that interacted with C1QTNF and hsa-miR-27b-3p through the RNA Interactomes Encyclopedia (ENCORI) database. The interaction network was visualized by Cytoscape software.

### 2.7 Real-time PCR assay

Total RNA was extracted from cells and tissues with the SteadyPure Universal RNA Extraction Kit II (Accurate Biotechnology, Hunan, China). Reverse transcription of RNA into cDNA was performed with Evo M-MLV RT Premix (Accurate Biotechnology, Hunan, China) according to the manufacturer’s instructions. qPCR assays were performed using the SYBR^®^ Green Premix Pro Taq HS qPCR Kit (Accurate Biotechnology, Hunan, China) and amplified in a LightCycler 480II (Roche). The primers used in this study were as follows: β-actin-F: AAG​TGT​GAC​GTG​GAC​ATC​CGC, β-actin-R: CCG​GAC​TCG​TCA​TAC​TCC​TGC​T, C1QTNF1-F: AAG​TTC​TAC​TGC​TAC​GTG​CCC, C1QTNF1-R: TGT​GCA​GGT​AGG​TCT​CCT​TCT.

### 2.8 IHC staining

Paraffin blocks containing tissue were cut into 4 μm sections and placed on slides. After deparaffinization and rehydration, they were incubated in 3% hydrogen peroxide to block endogenous peroxidase. Slides were next incubated with the C1QTNF1 (Proteintech, 12209-1-AP, Wuhan, China) antibody at a 1:50 dilution at 4°C overnight. Finally, the slides were washed with secondary antibody at room temperature for 30 min and stained with DAB and hematoxylin.

### 2.9 Cell culture and transfection

786-O cells were purchased from the Cell Bank of the Chinese Academy of Sciences. 786-O cells were placed in RPMI-1640 medium (Gibco; Thermo Fisher Scientific, Inc., Waltham, MA, United States) supplemented with 10% fetal bovine serum (FBS) and 1% penicillin/streptomycin. The above cells were cultured in a 5% CO_2_ cell incubator at 37°C. siRNA targeting C1QTNF1 was synthesized by Sangon Bio (Shanghai, China) and transfected using INTERFERin^®^ (Polyplus, Illkirch, France) according to the manufacturer’s instructions.

### 2.10 Cell proliferation and wound healing assays

The treated cells were seeded in 96-well plates with 2000 cells per well, and 10 μL CCK8 reagent was added to the corresponding wells every 24 h, respectively. The cells were incubated in a 5% CO_2_ incubator at 37°C for 1 h. The optical density (OD) of each well was measured using a microplate reader at a wavelength of 450 nm. The appropriate cells were seeded in 6-well plates, and when the cells grew to the appropriate density, the monolayer cells were scratched with the suction tip of 200 μL pipetter, and the cells were photographed by microscope 24 h after injury.

### 2.11 Statistical analysis

All statistical analyses were performed using R (V 3.6.3). Wilcoxon rank-sum test was used to investigate the levels of C1QTNF1 in both tumor and noncancer samples. The chi-square test, or Fisher exact test, was used to compare and analyze the statistical significance between the two groups of categorical variables. Univariate and multivariate analyses were performed using the Cox proportional hazards model to evaluate the independent prognostic factors of KIRC patients. Only *p* values of less than 0.05 (two-sided) were considered to indicate statistical significance. For data on C1qTNF1-related functions, statistical analysis was performed using GraphPad Prism 8.0.

## 3 Results

### 3.1 C1QTNF1 expression in multiple cancers

**FIGURE 1:**
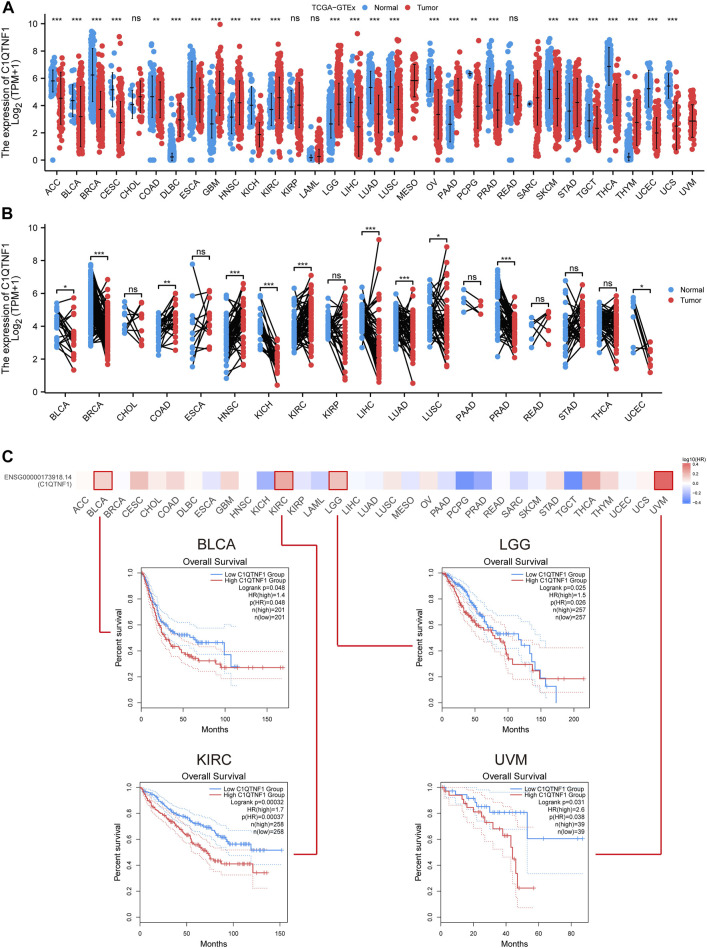
Differential expression of C1QTNF1 in pan-cancer and its prognostic value. **(A)** Expression levels of C1QTNF1 in multiple cancers and normal tissues in the TCGA-GTEx dataset. **(B)** Differential expression of C1QTNF1 between KIRC tissues and matched normal renal tissues in the TCGA-KIRC dataset. **(C)** Prognostic value of different C1QTNF1 levels on the GEPIA2 website in pan-cancer. NS, *p* > 0.05, *p < 0.05, **p < 0.01, ***p < 0.001.

The authors evaluated the expression levels of C1QTNF1 mRNA in 33 different cancer tissues and normal tissues using TCGA and GTEx data. Among them, C1QTNF1 expression levels were increased in several tumor tissues, including Lymphoid Neoplasm Diffuse Large B-cell Lymphoma (DLBC), Glioblastoma multiforme (GBM), Head and Neck squamous cell carcinoma (HNSC), and KIRC (Figure 1A). Meanwhile, we investigated the expression levels of C1QTNF1 in paired tumor tissues and normal tissues in the TCGA dataset and found that the expression levels of C1QTNF1 were increased in Colon adenocarcinoma (COAD), HNSC, and KIRC (Figure 1B). Kaplan-Meier survival curves showed that high expression of C1QTNF1 was significantly associated with a worse prognosis in Bladder Urothelial Carcinoma (BLCA), Brain Lower Grade Glioma (LGG), KIRC, and Uveal Melanoma (UVM) (Figure 1C).

### 3.2 Increased expression of C1QTNF1 suggests poor prognosis in KIRC patients

**FIGURE 2:**
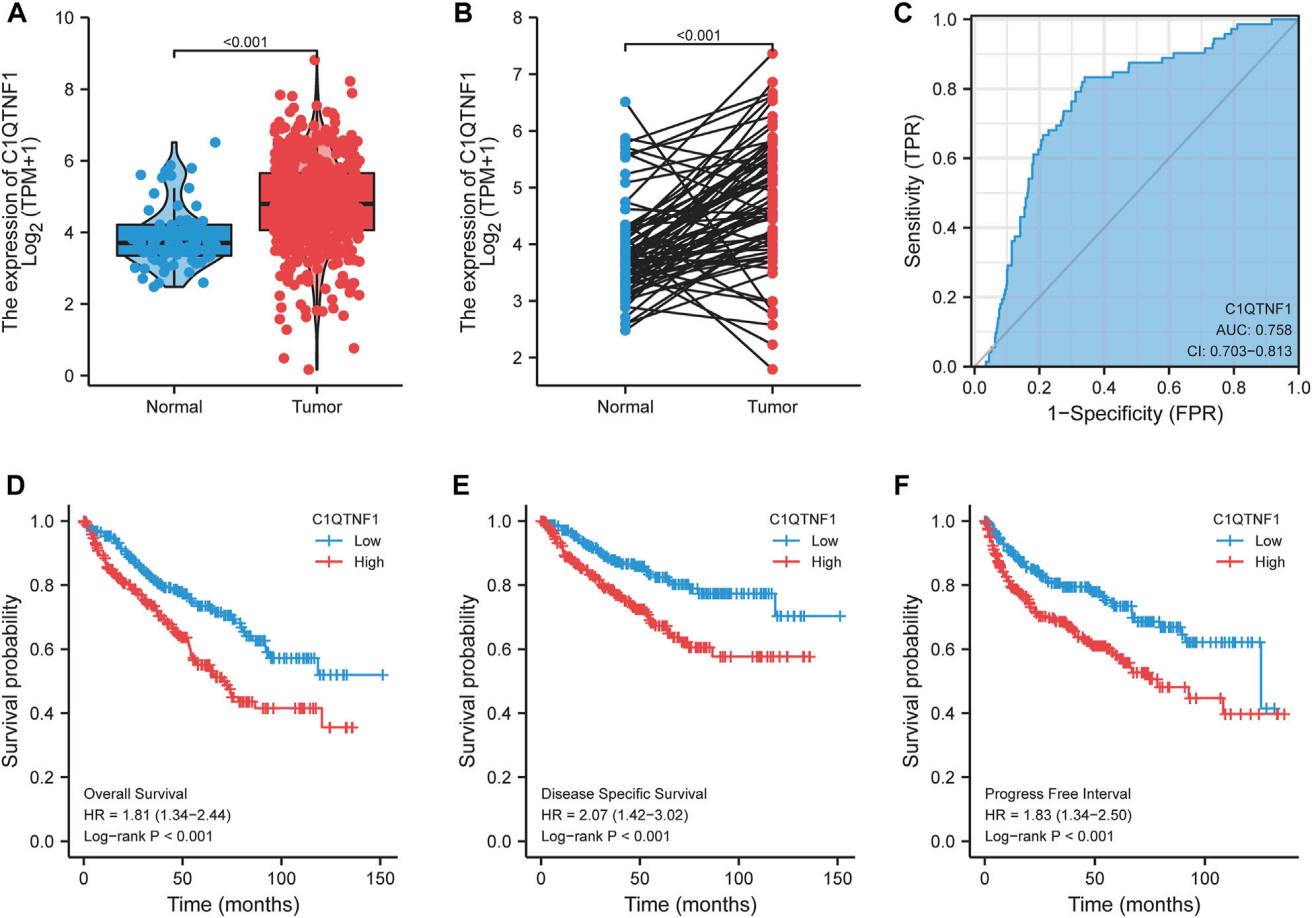
Expression level of C1QTNF1 in KIRC and its diagnostic and prognostic value. **(A, B)** Expression levels of C1QTNF1 in mismatched and matched KIRC tissues and normal tissues in the TCGA-KIRC dataset. **(C)** C1QTNF1 has a good ability to distinguish KIRC tissues from normal renal tissues. **(D–F)** KIRC patients with higher C1QTNF1 expression showed worse overall survival, disease specific survival, and progress free interval. *p* < 0.05 was considered statistically significant.

By comparing the expression of C1QTNF1 in unpaired and paired KIRC tumor tissues and normal tissue samples, we found that the expression level of C1QTNF1 was significantly increased (Figures 2A, B). The ROC curve showed that C1QTNF1 could effectively diagnose KIRC (Figure 2C). Survival analysis suggested that KIRC patients with high C1QTNF1 expression had a poor prognosis (Figures 2D–F). In addition, the prognostic analysis of C1QTNF1 using the Kaplan-Meier Plotter online website also obtained the same results (Supplementary Figure S1).

### 3.3 C1QTNF1 is associated with the clinical characteristics of KIRC patients

**FIGURE 3:**
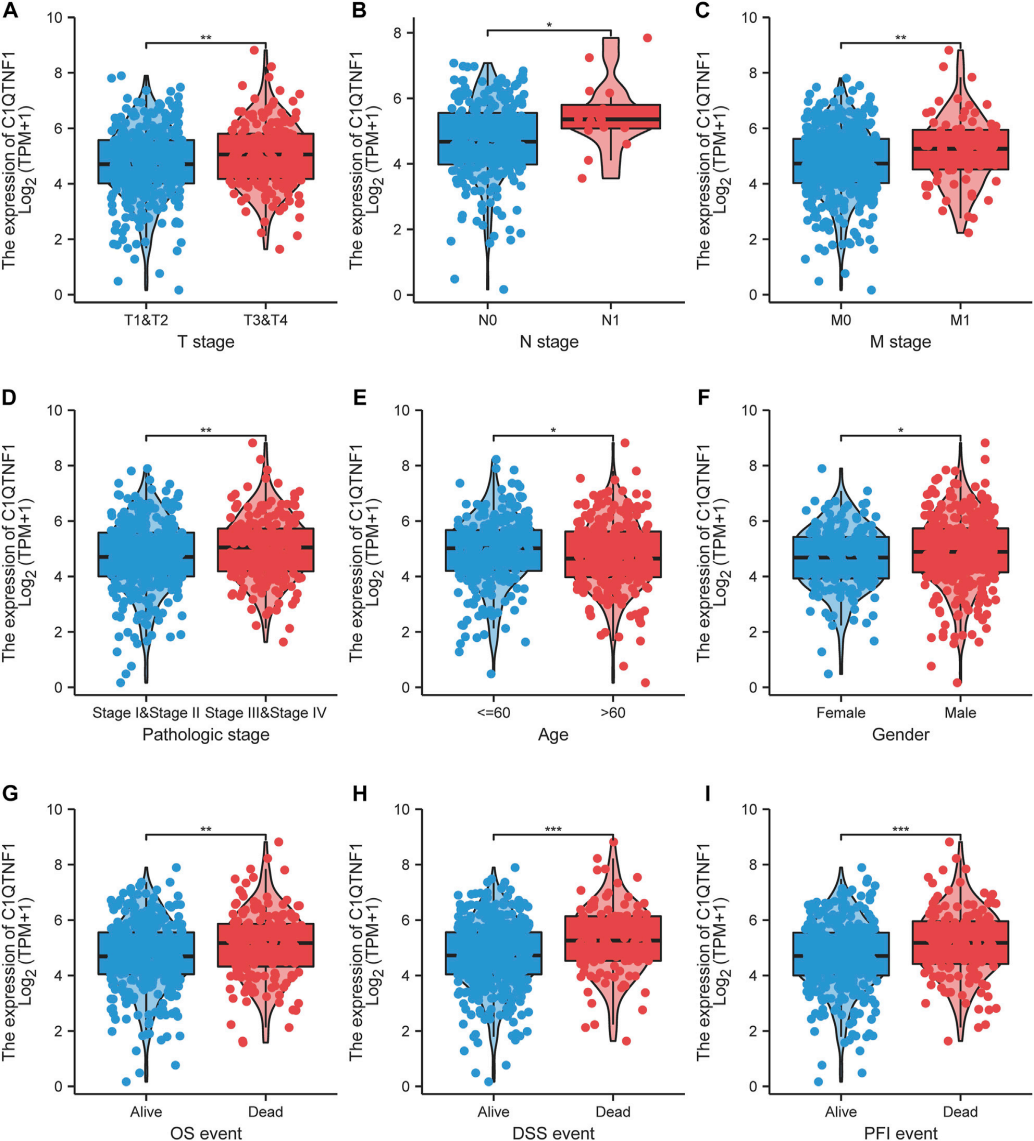
Association of C1QTNF1 with clinical characteristics of KIRC patients. **(A)** T stage; **(B)** N stage; **(C)** M stage; **(D)** Pathologic stage; **(E)** Age; **(F)** Gender; **(G)** OS event; **(H)** DSS event; **(I)** PFI event. NS, *p* > 0.05, *p < 0.05, **p < 0.01, ***p < 0.001.

As shown in Figures 3A–I, C1QTNF1 expression was significantly correlated with TNM stage, Pathologic stage, age, sex, Overall Survival (OS) events, Disease Specific Survival (DSS) events, and Progress Free Interval (PFI) events (Table 1). Logistic regression analysis also indicated that NCAPG2 expression was significantly correlated with poor clinicopathological prognosis, including T stage (T3, T4 vs. T1, T2), N stage (N1 vs. N0), M stage (M1 vs. M0), Pathological stage (III and IV vs. I and II), and age (>60 vs.≤60) (Table 2).

**TABLE 1:** Clinicopathological features of KIRC patients.

Characteristic	Low expression of C1QTNF1	High expression of C1QTNF1	p
n	269	270	
T stage, n (%)			0.002
T1	144 (26.7%)	134 (24.9%)	
T2	46 (8.5%)	25 (4.6%)	
T3	77 (14.3%)	102 (18.9%)	
T4	2 (0.4%)	9 (1.7%)	
N stage, n (%)			0.009
N0	134 (52.1%)	107 (41.6%)	
N1	3 (1.2%)	13 (5.1%)	
M stage, n (%)			0.002
M0	226 (44.7%)	202 (39.9%)	
M1	26 (5.1%)	52 (10.3%)	
Pathologic stage, n (%)			0.003
Stage I	142 (26.5%)	130 (24.3%)	
Stage II	39 (7.3%)	20 (3.7%)	
Stage III	58 (10.8%)	65 (12.1%)	
Stage IV	29 (5.4%)	53 (9.9%)	
Gender, n (%)			0.116
Female	102 (18.9%)	84 (15.6%)	
Male	167 (31%)	186 (34.5%)	
Age, n (%)			0.007
≤60	118 (21.9%)	151 (28%)	
>60	151 (28%)	119 (22.1%)	
Age, median (IQR)	62 (53, 72)	59 (51, 68)	0.002

**TABLE 2:** Logistic regression analysis of correlation between C1QTNF1 and clinical features.

Characteristics	Total (N)	Odds ratio (OR)	*p*-value
T stage (T3 and T4 vs. T1 and T2)	539	1.679 (1.176–2.405)	0.004
N stage (N1 vs. N0)	257	5.427 (1.697–24.110)	0.010
M stage (M1 vs. M0)	506	2.238 (1.359–3.765)	0.002
Pathologic stage (Stage III&Stage IV vs. Stage I and Stage II)	536	1.637 (1.153–2.330)	0.006
Gender (Male vs. Female)	539	1.352 (0.948–1.934)	0.097
Age (>60 vs.≤60)	539	0.616 (0.438–0.865)	0.005

### 3.4 Pathway and functional enrichment analysis of C1QTNF1-related differential genes

**FIGURE 4:**
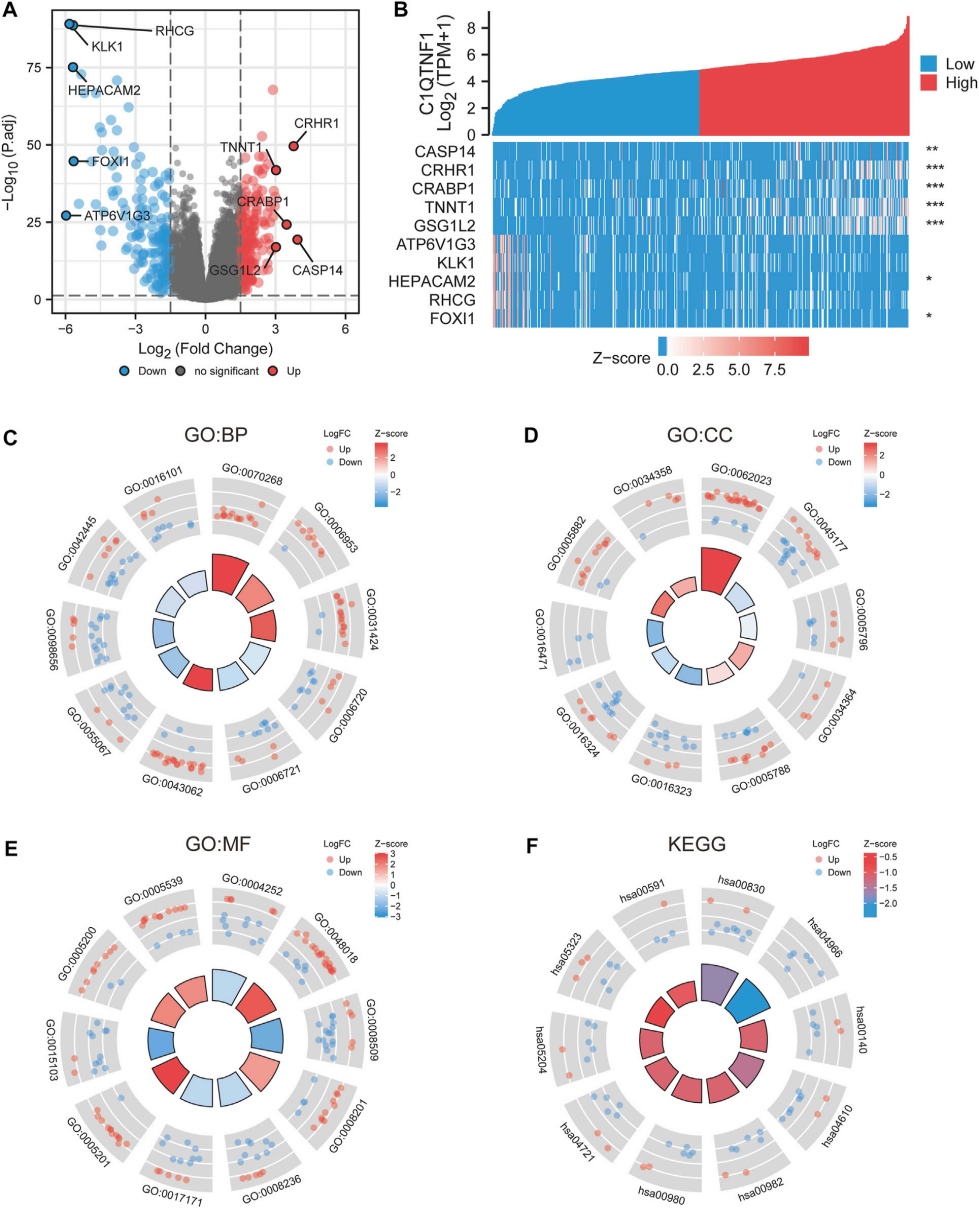
Differential genes and functional enrichment between C1QTNF1 high and low expression groups. **(A)** DEGs volcano plot, with blue and red dots indicating significantly downregulated and upregulated DEGs, respectively. **(B)** Heat map of the top five related genes in the high C1QTNF1 expression group and the low C1QTNF1 expression group. **(C–F)** GO and KEGG analysis of DEGs.

We analyzed the gene expression profile between the C1QTNF1 high expression group and the low expression group, and 357 differentially expressed genes (DEGs) were obtained. These included 170 downregulated genes and 187 upregulated genes (Figure 4A). The heat map shows the expression of the top five genes in the high versus low expression groups (Figure 4B). In addition, we performed Gene Ontology (GO) functional enrichment and Kyoto Encyclopedia of Genes and Genomes (KEGG) pathway analysis of differentially expressed genes between high and low-expression groups (Tables 3, 4). The main biological processes included cornification, acute-phase response, keratinization, isoprenoid metabolic process, terpenoid metabolic process, extracellular structure organization, monovalent inorganic cation homeostasis, anion transmembrane transport, hormone metabolic process, and diterpenoid metabolic process (Figure 4C). The most enriched cellular components were collagen-containing extracellular matrix, apical part of cell, Golgi lumen, high-density lipoprotein particle, endoplasmic reticulum lumen, basolateral plasma membrane, apical plasma membrane, vacuolar proton-transporting V-type ATPase complex, intermediate filament, and plasma lipoprotein particle (Figure 4D). The most enriched molecular functions were serine-type endopeptidase activity, receptor ligand activity, anion transmembrane transporter activity, heparin binding, serine-type peptidase activity, serine hydrolase activity, extracellular matrix structural constituent, inorganic anion transmembrane transporter activity, structural constituent of cytoskeleton, and glycosaminoglycan binding (Figure 4E). The KEGG pathway was enriched in Retinol metabolism, Collecting duct acid secretion, Steroid hormone biosynthesis, Complement, and coagulation cascades, Drug metabolism - cytochrome P450, Metabolism of xenobiotics by cytochrome P450, Synaptic vesicle cycle, Chemical carcinogenesis, Rheumatoid arthritis, and Linoleic acid metabolism (Figure 4F).

**TABLE 3:** GO enrichment analysis.

Ontology	ID	Description	GeneRatio	BgRatio	p-Value
BP	GO:0070268	cornification	14/322	112/18,670	8.16844E-09
BP	GO:0006953	acute-phase response	9/322	47/18,670	9.24978E-08
BP	GO:0031424	keratinization	17/322	224/18,670	3.75682E-07
BP	GO:0006720	isoprenoid metabolic process	13/322	139/18,670	8.70814E-07
BP	GO:0006721	terpenoid metabolic process	12/322	120/18,670	1.12715E-06
BP	GO:0043062	extracellular structure organization	23/322	422/18,670	1.29364E-06
BP	GO:0055067	monovalent inorganic cation homeostasis	13/322	154/18,670	2.77519E-06
BP	GO:0098656	anion transmembrane transport	18/322	288/18,670	2.86174E-06
BP	GO:0042445	hormone metabolic process	16/322	232/18,670	2.91929E-06
BP	GO:0016101	diterpenoid metabolic process	11/322	110/18,670	3.16297E-06
CC	GO:0062023	collagen-containing extracellular matrix	27/340	406/19,717	2.45826E-09
CC	GO:0045177	apical part of cell	20/340	384/19,717	1.27061E-05
CC	GO:0005796	Golgi lumen	9/340	102/19,717	6.79779E-05
CC	GO:0034364	high-density lipoprotein particle	5/340	26/19,717	7.22903E-05
CC	GO:0005788	endoplasmic reticulum lumen	16/340	309/19,717	0.00010012
CC	GO:0016323	basolateral plasma membrane	13/340	217/19,717	0.000106688
CC	GO:0016324	apical plasma membrane	16/340	318/19,717	0.000139454
CC	GO:0016471	vacuolar proton-transporting V-type ATPase complex	4/340	17/19,717	0.000173105
CC	GO:0005882	intermediate filament	12/340	214/19,717	0.000358123
CC	GO:0034358	plasma lipoprotein particle	5/340	37/19,717	0.000410123
MF	GO:0004252	serine-type endopeptidase activity	14/304	160/17,697	7.02317E-07
MF	GO:0048018	receptor ligand activity	25/304	482/17,697	9.93195E-07
MF	GO:0008509	anion transmembrane transporter activity	20/304	327/17,697	1.0333E-06
MF	GO:0008201	heparin binding	14/304	169/17,697	1.35878E-06
MF	GO:0008236	serine-type peptidase activity	14/304	182/17,697	3.27091E-06
MF	GO:0017171	serine hydrolase activity	14/304	186/17,697	4.21794E-06
MF	GO:0005201	extracellular matrix structural constituent	13/304	163/17,697	4.94288E-06
MF	GO:0015103	inorganic anion transmembrane transporter activity	12/304	146/17,697	8.41712E-06
MF	GO:0005200	structural constituent of cytoskeleton	10/304	102/17,697	1.01926E-05
MF	GO:0005539	glycosaminoglycan binding	15/304	229/17,697	1.04661E-05

**TABLE 4:** KEGG enrichment analysis.

Ontology	ID	Description	GeneRatio	BgRatio	*p*-value
KEGG	hsa00830	Retinol metabolism	9/151	68/8076	4.23273E-06
KEGG	hsa04966	Collecting duct acid secretion	6/151	27/8076	8.27733E-06
KEGG	hsa00140	Steroid hormone biosynthesis	7/151	61/8076	0.00013017
KEGG	hsa04610	Complement and coagulation cascades	8/151	85/8076	0.000176726
KEGG	hsa00982	Drug metabolism - cytochrome P450	7/151	71/8076	0.00033954
KEGG	hsa00980	Metabolism of xenobiotics by cytochrome P450	7/151	77/8076	0.000559259
KEGG	hsa04721	Synaptic vesicle cycle	7/151	78/8076	0.000604919
KEGG	hsa05204	Chemical carcinogenesis	7/151	82/8076	0.000818032
KEGG	hsa05323	Rheumatoid arthritis	7/151	93/8076	0.001718651
KEGG	hsa00591	Linoleic acid metabolism	4/151	29/8076	0.001938271

### 3.5 Relationship between C1QTNF1 and immune infiltration

**FIGURE 5:**
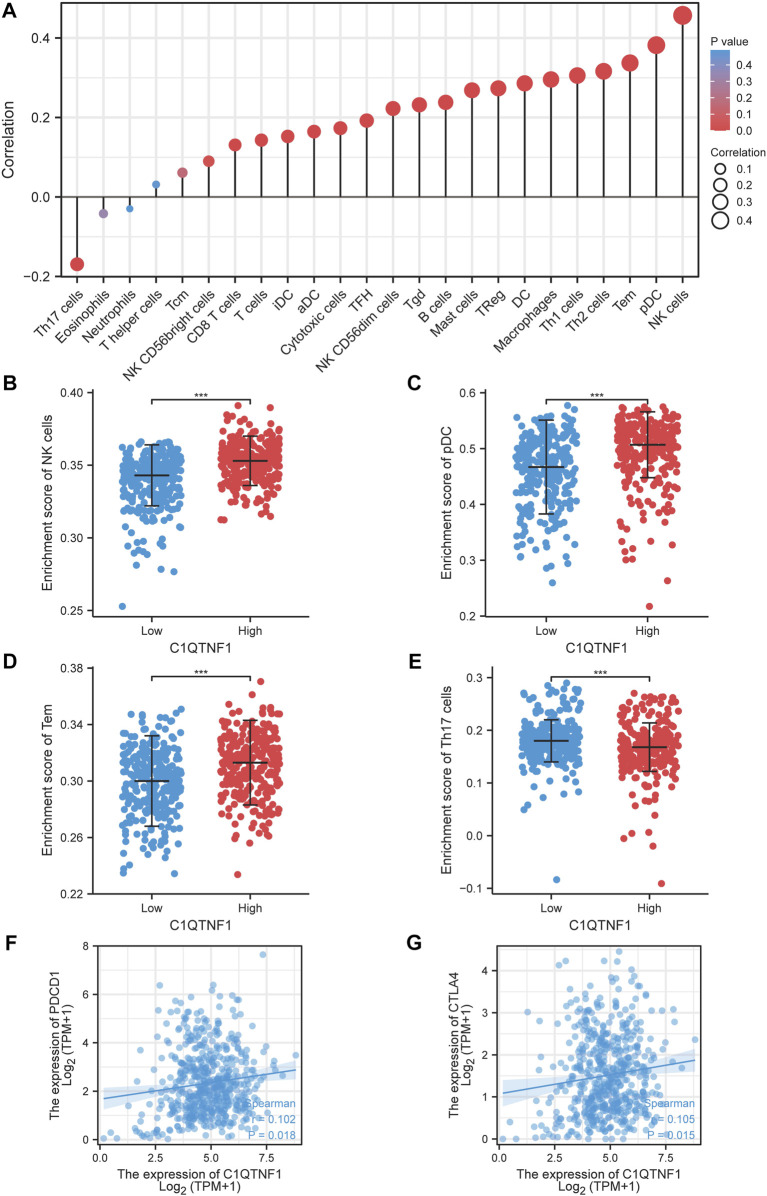
Relationship between C1QTNF1 expression and KIRC immune infiltration. **(A)** Relationship between C1QTNF1 expression and abundance of multiple immune cells, the size of the dots represents the absolute value of Spearman’s correlation coefficient. **(B–E)** Comparison of immune infiltration levels of immune cells (including NK cells, pDC, Tem cells, and Th17) between the C1QTNF1 high expression group and low expression group. **(F–G)** Correlation of C1QTNF1 with immune checkpoints.

We explored the relationship between C1QTNF1 and immune cell infiltration based on the ssGSEA method. C1QTNF1 and NK cells, pDC, Tem, Th2 cells, Th1 cells, Macrophages, DC, TReg, Mast cells, B-cells, Tgd, NK CD56dim cells, TFH, Cytotoxic cells, aDC, iDC, T-cells, CD8 T-cells, and NK CD56 bright cells were positively correlated, while Th17 cells were negatively correlated (Figure 5A). In addition, we analyzed the infiltration levels of several immune cells with the highest correlation between the C1QTNF1 high expression group and the low expression group and found that the infiltration levels of NK cells, pDC, Tem, and Th17cells were statistically significant between the C1QTNF1 high expression group and the low expression group (Figures 5B–E). Finally, we also found that two immune checkpoints, PDCD1 and CTLA4, were positively correlated with the expression level of C1QTNF1 (Figures 5F, G). In addition, we found a strong association between C1QTNF1 and TIL infiltration through the TISIDB database, and it was also positively correlated with two immune checkpoints, PDCD1 and CTLA4 (Supplementary Figure S2). This suggests that C1QTNF1 may be closely related to the immune infiltration of tumors.

### 3.6 Prognostic analysis of C1QTNF1 expression

**FIGURE 6:**
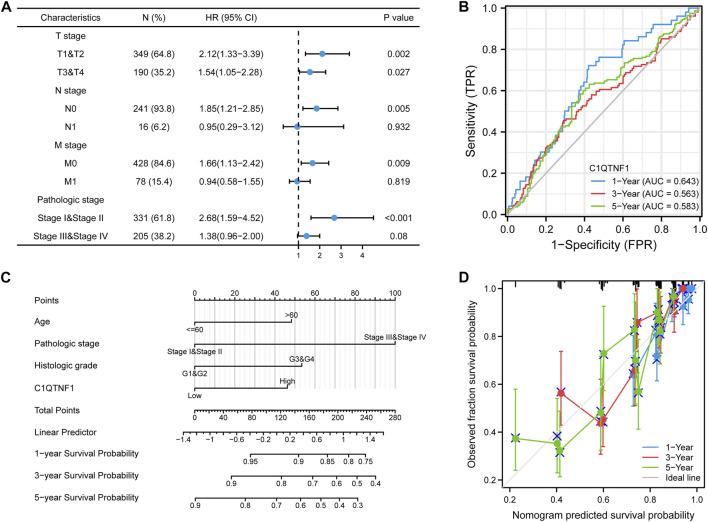
Prognostic analysis of C1QTNF1 subtypes and construction of a nomogram. **(A)** Prognostic forest plots of C1QTNF1 in different subgroups. **(B)** time-dependent ROC curves. **(C)** Nomogram. **(D)** Calibration curve.

Analysis of the prognostic impact of C1QTNF1 on different clinical subgroups showed that C1QTNF1 was a risk factor for stages T1 and T2, T3 and T4, N0, M0, and Stage I and Stage II (HR > 1, *p* < 0.05) (Figure 6A). Time-dependent ROC curve analysis showed that the AUC values for predicting the 1-, 3-, and 5-year survival rates of KIRC patients based on C1QTNF1 expression levels were all greater than 0.5 (Figure 6B). Univariate and multivariate Cox analyses were performed by combining multiple clinical factors with C1QTNF1 to finally determine the independent factors predicting the prognosis (Table 5). The predictors obtained above were used to construct a prediction model, and a nomogram was drawn to assess the risk probability (Figure 6C). In addition, the calibration plots showed that the model had good predictive value for the survival time of patients at 1, 3, and 5 years (Figure 6D).

**TABLE 5:** Univariate and multivariate Cox regression analysis.

Characteristics	Total(N)	Univariate analysis		Multivariate analysis	
		Hazard ratio (95% CI)	*p*-value	Hazard ratio (95% CI)	*p*-value
T stage	539				
T1 and T2	349	References			
T3 and T4	190	3.228 (2.382–4.374)	**<0.001**	0.975 (0.424–2.246)	0.953
N stage	257				
N0	241	References			
N1	16	3.453 (1.832–6.508)	**<0.001**	1.705 (0.831–3.496)	0.146
Pathologic stage	536				
Stage I and Stage II	331	References			
Stage III and Stage IV	205	3.946 (2.872–5.423)	**<0.001**	2.567 (1.074–6.133)	**0.034**
Gender	539				
Female	186	References			
Male	353	0.930 (0.682–1.268)	0.648		
Age	539				
≤60	269	References			
>60	270	1.765 (1.298–2.398)	**<0.001**	1.638 (1.069–2.510)	**0.023**
Race	532				
White	467	References			
Black or African American	57	0.857 (0.465–1.582)	0.622		
Asian	8	0.545 (0.076–3.905)	0.546		
C1QTNF1	539				
Low	269	References			
High	270	1.819 (1.338–2.472)	**<0.001**	1.610 (1.040–2.491)	**0.033**
Histologic grade	531				
G1 and G2	249	References			
G3 and G4	282	2.702 (1.918–3.807)	**<0.001**	1.871 (1.145–3.058)	**0.012**

The bold values represents *p values* are statistically significant.

### 3.7 Knockdown of C1QTNF1 inhibited the proliferation, invasion, and migration of KIRC cell lines

**FIGURE 7:**
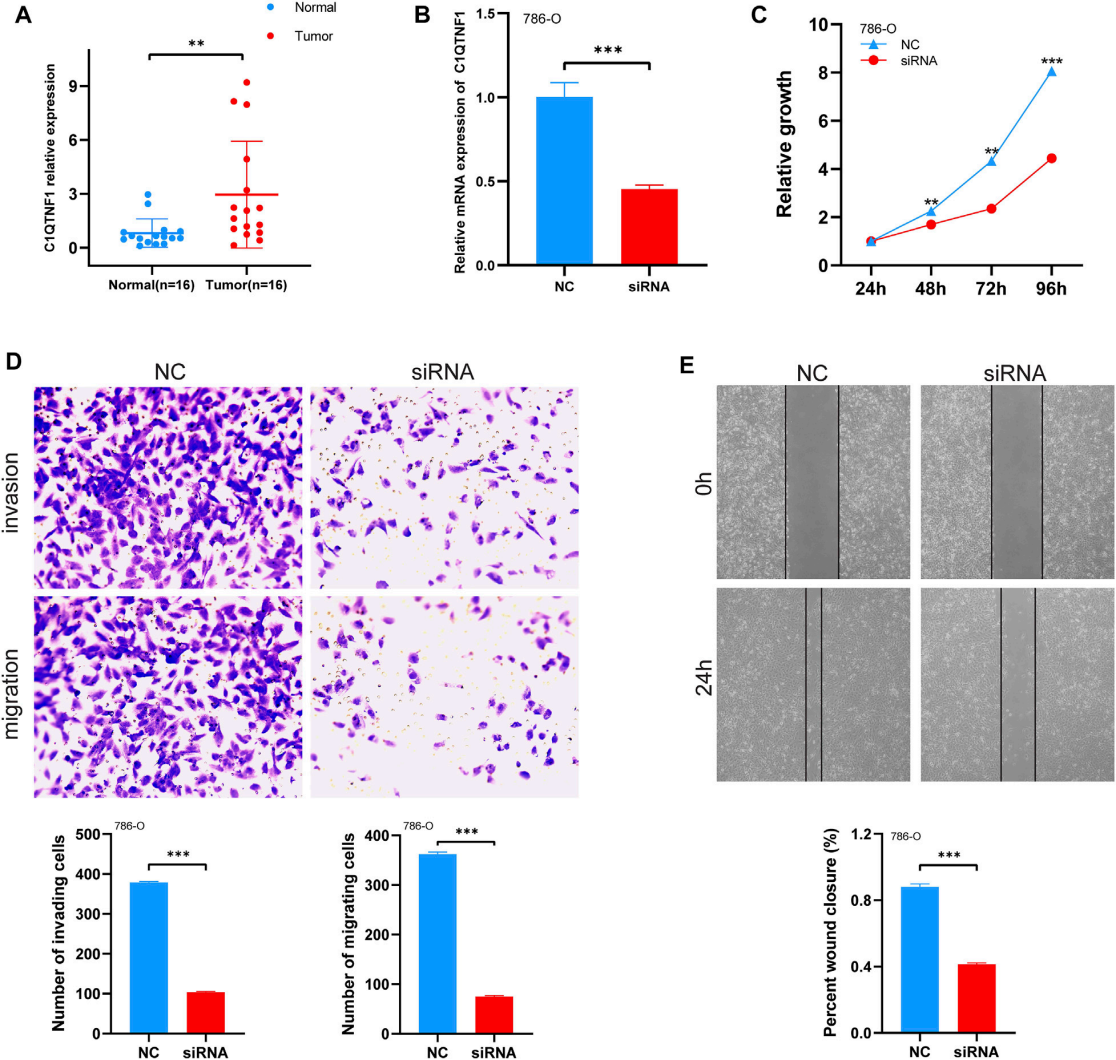
Expression levels of C1QTNF1 in clinical samples and changes in cell proliferation, migration, and invasion after knockdown of C1QTNF1. **(A)** qRT-PCR analysis of C1QTNF1 transcript levels in KIRC and normal tissues. **(B)** To verify the knockdown efficiency of C1QTNF1 in 786-O cells using a qPCR assay. **(C–E)** CCK8 and transwell assays were used to detect the changes in cell proliferation, migration, and invasion after the C1QTNF1 knockdown.

To verify the expression level of C1QTNF1 in KIRC, the authors examined the expression of C1QTNF1 in clinical samples using qRT-PCR. The results confirmed that C1QTNF1 was elevated in KIRC tissues (Figure 7A). Immunohistochemical staining results also showed that the expression of C1QTNF1 protein was increased in KIRC tissues (Supplementary Figure S3). Subsequently, we knocked down C1QTNF1 in KIRC cell lines, and qRT-PCR showed that the expression of C1QTNF1 was significantly reduced after knockdown (Figure 7B). CCK-8, wound healing assay, and Transwell assay confirmed that knockdown of C1QTNF1 inhibited the proliferation, invasion, and migration of KIRC cells (Figures 7C–E).

### 3.8 Construction of interaction networks and potential miRNAs analysis of C1QTNF1

Using GeneMANIA, we generated a gene-gene network of 20 potential C1QTNF1 interactions, They are AVPR2, SGTA, HEYL, C1QTNF6, MAFF, SOD3, BDKRB2, PRPF31, GADD45G, MFGE8, CST3, IRF4, ABLIM1, GSN, FSTL3, SF3A1, CMKLR1, ITGA7, SYNPO, and PLA2G 5 (Supplementary Figure S4A). Chemical agents associated with the C1QTNF1 gene were predicted from the CDC database (Supplementary Figure S4B) (Supplementary Table S1). Increasing evidence has confirmed that ncRNAs can regulate gene expression in various ways in tumors (Qi et al., 2015). To determine whether C1QTNF1 is regulated by certain ncRNAs, we predicted the upstream miRNAs that might bind to C1QTNF1 and finally found 28 miRNAs (Supplementary Figure S4C) (Supplementary Table S2). Due to the mechanism by which upstream miRNAs negatively regulate C1QTNF1 expression at the post-transcriptional level, there should be a negative relationship between C1QTNF1 and upstream miRNAs. Therefore, the correlation between C1QTNF1 and 28 miRNAs was examined in the ENCORI database. The results suggested a negative correlation between hsa-miR-27b-3p and C1QTNF1 (Supplementary Figure S4D). We then determined the expression level of hsa-miR-27b-3p in the TCGA-KIRC database. The results showed that the expression level of hsa-miR-27b-3p in KIRC was lower than that in adjacent normal tissue controls (Supplementary Figure S4E). We also explored the correlation between the hsa-miR-27b-3p expression level and the prognosis of KIRC patients. The results showed that high expression of hsa-miR-27b-3p was significantly associated with a good prognosis of KIRC (Supplementary Figure S4F). Combined with correlation analysis, expression analysis, and survival analysis, we suggest that hsa-miR-27b-3p may be the most likely upstream regulatory miRNA of C1QTNF1 in KIRC.

### 3.9 Exploration of potential lncRNAs upstream of hsa-miR-27b-3p

Next, ENCORI was used to predict the lncRNAs upstream of hsa-miR-27b-3p. Finally, 143 possible lncRNAs were obtained (Supplementary Table S3). The competing endogenous RNA (ceRNA) hypothesis suggests that lncRNA competitively binds tumor suppressor miRNAs to reduce the inhibitory effect of miRNAs on target mRNAs. It can be seen that there is a negative correlation between lncRNA and target miRNA in the ceRNA network, while there is a positive correlation between lncRNA and target mRNA. Therefore, we evaluated the expression correlation of hsa-miR-27b-3p/C1QTNF1 and 143 lncRNAs in the ENCORI database. The results suggested that CYTOR, AC040970.1, AC016717.2, AC010980.2, and LINC02381 were negatively correlated with the expression of hsa-miR-27b-3p, but positively correlated with the expression of C1QTNF1 (Supplementary Figures S5A, B). Subsequently, we performed expression analysis and prognostic analysis of the above 5 lncRNAs in KIRC (Supplementary Figures S5C, D). CYTOR and AC040970.1 may be the two most potential lncRNAs upstream of the hsa-miR-27b-3p/C1QTNF1 axis in KIRC.

## 4 Discussion

Renal-cell carcinoma is a cancer worldwide and is the second most common cause of death from urologic tumors (Sung et al., 2021). As the most common type of renal cell carcinoma, KIRC is the main cause of death in patients with renal cell carcinoma (Hsieh et al., 2017). At present, the treatment of local renal cell carcinoma is still surgical resection (Ljungberg et al., 2015). Because the treatment effect of advanced renal cell carcinoma is still not satisfactory. Therefore, there is an urgent need to find a potential prognostic marker as a molecular therapeutic target to improve the overall prognosis of patients with advanced RCC.

As a public health problem worldwide, obesity is associated with a high incidence of cancer (Avgerinos et al., 2019). At present, some scholars believe that weight gain is associated with an increased incidence of kidney cancer. Obese patients’ adipose tissue triggers inflammation, hypoxia, and angiogenesis, which may subsequently promote tumor formation (Lee et al., 2015). Adipose tissue secretes various adipokines, and their association with cancer is diverse. It has been suggested that adipose-tissue signaling factors may be responsible for the interrelationship between obesity and cancer (Stone et al., 2018). For example, leptin, which has pro-inflammatory effects, can increase the tumorigenicity of cancer stem cells (Lee et al., 2015), and leptin has been associated with an increased risk of renal cancer (Yoon et al., 2019). It has recently been shown that Chemerin, as a multifunctional adipokine, can inhibit the fatty acid oxidation of KIRC, thereby increasing the resistance of cancer cells to ferroptosis (Tan et al., 2021).

The CTRP family belongs to the adipokine superfamily, which is involved in glucose and lipid metabolism in obesity metabolic disorders (Wong et al., 2008; Shanaki et al., 2020). Among them, CTRP3, CTRP4, CTRP6, and CTRP8 are involved in the progression of various cancers, including osteosarcoma, liver cancer, colon cancer, and lung cancer (Kong et al., 2021). C1QTNF1 (CTRP1), a member of this family, has previously been shown to be significantly upregulated in GBM tissues, and its knockout can affect the proliferation and migration of human GBM cells (Chen and Su, 2019). In addition, it has been shown that C1QTNF1 expression is increased in obese mice, and overexpression of C1QTNF1 can inhibit the transcription and protein expression of the tumor suppressor gene P53, leading to tumor progression (Park et al., 2021). Knockdown of C1QTNF1 in A549 and HCT116 resulted in tumor cell proliferation, invasion, and growth (Park et al., 2022).

In this study, we analyzed the expression of C1QTNF1 in normal and tumor tissues from the TCGA and GTEx datasets and found that C1QTNF1 was upregulated in multiple tumor tissues. Analysis on the GEPIA2 website showed that high expression of C1QTNF1 can lead to a poor prognosis in patients with a variety of cancers. In the TCGA-KIRC dataset, C1QTNF1 expression was found to be upregulated compared with normal renal tissues. The ROC curve showed that C1QTNF1 was a useful marker to distinguish KIRC from normal renal tissues. The prognostic analysis of C1QTNF1 in KIRC patients suggested that KIRC patients with high expression of C1QTNF1 tended to have a poor prognosis. C1QTNF1 expression was associated with multiple clinical features. To further analyze the potential function of C1QTNF1, the differentially expressed genes between C1QTNF1 high and low expression groups were analyzed. We found that these genes were mainly enriched in Retinol metabolism, Collecting duct acid secretion, and Steroid hormone biosynthesis pathways. Stratified analysis showed that C1QTNF1 was a very effective predictor within subgroups. In addition, Cox regression analysis showed that C1QTNF1 could be used as an independent prognostic factor for KIRC patients.

The tumor microenvironment is closely related to the occurrence and development of tumors (Roma-Rodrigues et al., 2019), and tumor cells, as an important part of the tumor immune microenvironment, can affect the efficacy of tumor treatment and the prognosis of tumor patients (Zhang et al., 2018; Lyu et al., 2020). Our study analyzed the association between C1QTNF1 expression and tumor immune cell infiltration. The results showed that C1QTNF1 expression and NK cells, pDC, Tem, Th2 cells, Th1 cells, Macrophages, DC, TReg, Mast cells, B cells, Tgd, NK CD56dim cells, TFH, Cytotoxic cells, aDC, iDC, T-cells, CD8 T-cells, and NK CD56bright cells were positively correlated with each other. In KIRC, when the infiltration level of Treg cells is high, the prognosis of patients is often poor (Zhang et al., 2019). In addition, some studies have shown that CXCL13-secreting CD8+T cells impair the immune function of total CD8+T cells, and suggest that KIRC patients have a poor prognosis (Dai et al., 2021). This suggests that C1QTNF1 may be involved in the process of tumor immunity. Tumor development is closely related to tumor immune evasion. In this process, immune checkpoints play an important role. PDCD1 and CTLA4 are two important immune checkpoint proteins involved in the process of immune evasion (Krummel and Allison, 1996; Goodman et al., 2017). At present, a variety of drugs targeting immune checkpoint proteins have been approved for the treatment of patients (Lee et al., 2019). C1QTNF1 expression was positively correlated with these two immune checkpoint proteins. This suggests that targeting C1QTNF1 may be an effective approach to improving the efficacy of immunotherapy in KIRC patients.

To find the potential upstream molecular mechanism of C1QTNF1, we analyzed the miRNAs associated with C1QTNF1 in KIRC. The ceRNA hypothesis suggests (Salmena et al., 2011) that there should be a negative correlation between C1QTNF1 and upstream miRNAs due to the mechanism by which upstream miRNAs negatively regulate C1QTNF1 expression at the post-transcriptional level. Through correlation analysis, expression level analysis and prognostic analysis, we found that hsa-miR-27b-3p was the most likely upstream tumor suppressor miRNA of C1QTNF1. At present, several studies have shown that miR-27b-3p plays the role of a tumor suppressor miRNA (Sun et al., 2017; Liang and Zhang, 2021). Based on the ceRNA hypothesis, 143 lncRNAs upstream of hsa-miR-27b-3p/C1QTNF1 were predicted. By performing expression analysis, survival analysis, and correlation analysis, two most likely upregulated lncRNAs were identified, including CYTOR and AC040970.1. CYTOR has been reported to function as an oncogene in a variety of malignancies (Wei et al., 2021; Wang et al., 2022; Wu et al., 2023). The Ensembl ID for AC040970.1 is ENSG00000253210. Few studies have been conducted on AC040970.1; therefore, further investigation is important. In conclusion, CYTOR and AC040970.1/hsa-miR-27b-3p axis were identified as potential regulatory pathways in KIRC.

This study has deepened our understanding of the association between C1QTNF1 and KIRC. However, some limitations still exist. First, the study made use of fewer databases, which can lead to selection bias. Secondly, although we explored the correlation between C1QTNF1 and immune infiltration in KIRC patients, there is a lack of experiments to verify it. In this study, only the CCK-8 assay was performed and no colony formation assay was performed, which may not be able to avoid errors due to the presence of cells that can no longer divide. The protein expression level after C1QTNF1 knockdown was not verified, so it is not possible to determine whether the protein expression level is also decreased. Finally, more *in vitro* and *in vivo* experiments are still needed to explore the underlying molecular mechanisms of C1QTNF1 in KIRC.

## 5 Conclusion

In this study, we explored the expression of C1QTNF1 in KIRC and its prognostic value. We analyzed the association of C1QTNF1 with immune infiltration in KIRC patients. A possible upstream regulatory mechanism for C1QTNF1 was identified. In conclusion, C1QTNF1 is a promising prognostic factor, and these findings may provide new therapeutic approaches for clinical management and prognostic evaluation of KIRC.

## Data Availability

The raw data supporting the conclusion of this article will be made available by the authors, without undue reservation.
