# Supplementary figures and images for: High C1QTNF1 expression mediated by potential ncRNAs is associated with poor prognosis and tumor immunity in kidney renal clear cell carcinoma
Source: Front Mol Biosci. 2023 Jul 17;10:1201155. doi: 10.3389/fmolb.2023.1201155 (PMC10387556; doi:10.3389/fmolb.2023.1201155)

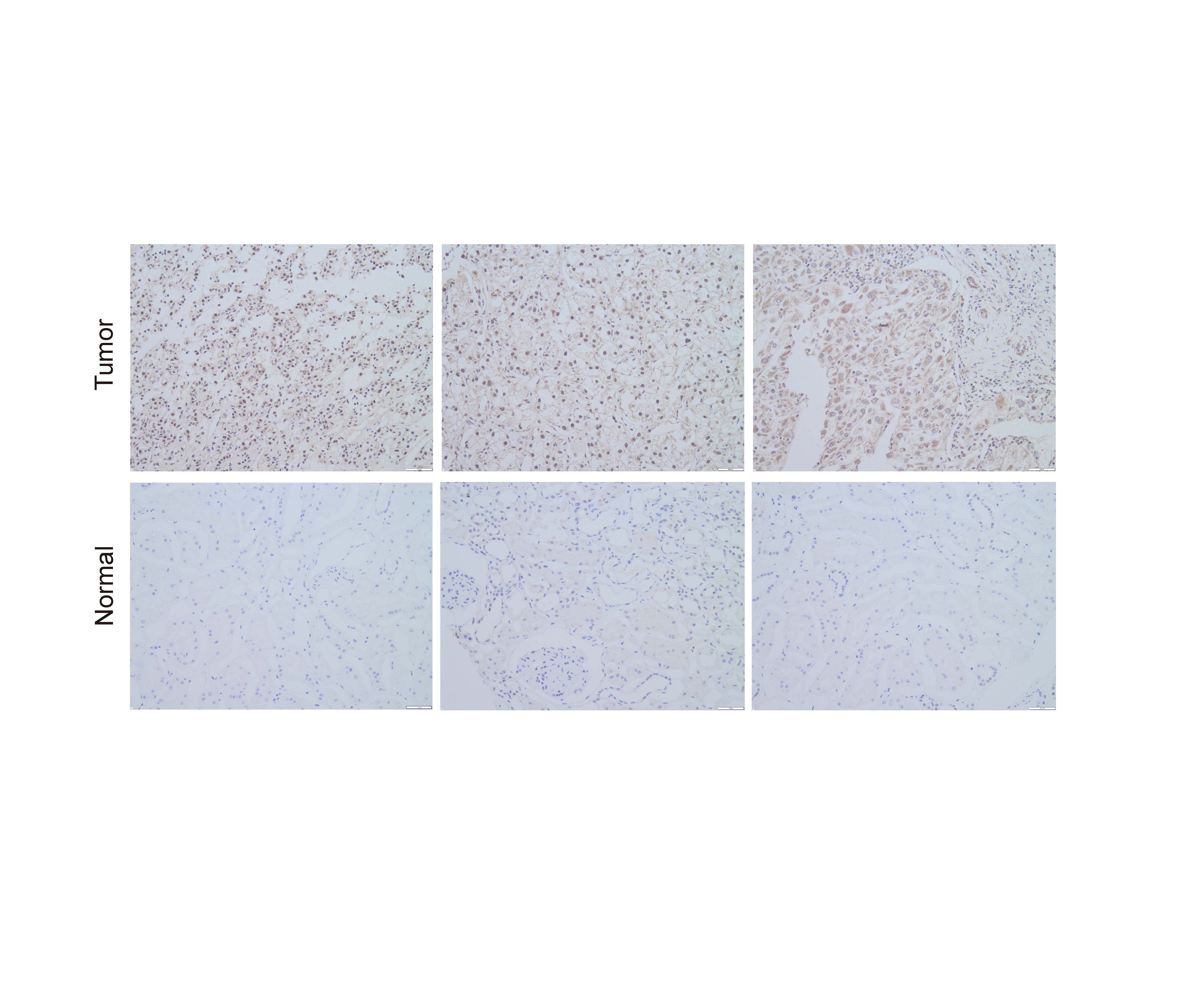

Supplement: Supplementary file 2 [file Image3.JPEG]

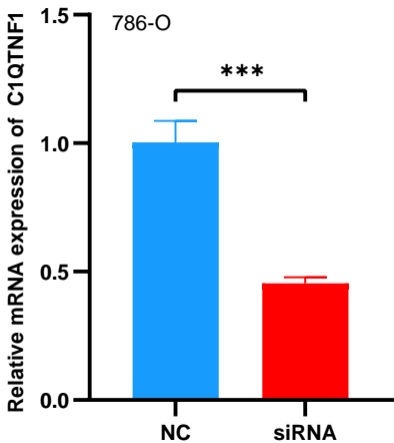

Supplement: Supplementary file 3 [file DataSheet3.ZIP › C1QTNF1 original data 3/siC1QTNF1-pcr/786-C1Q-敲除.pdf]

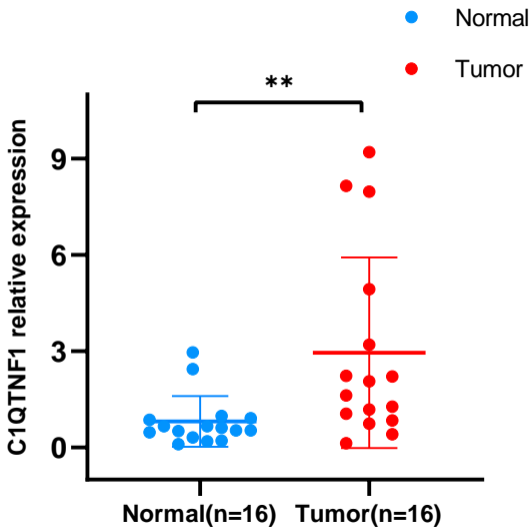

Supplement: Supplementary file 3 [file DataSheet3.ZIP › C1QTNF1 original data 3/Tissue PCR Validation/组织PCR-16对.pdf]

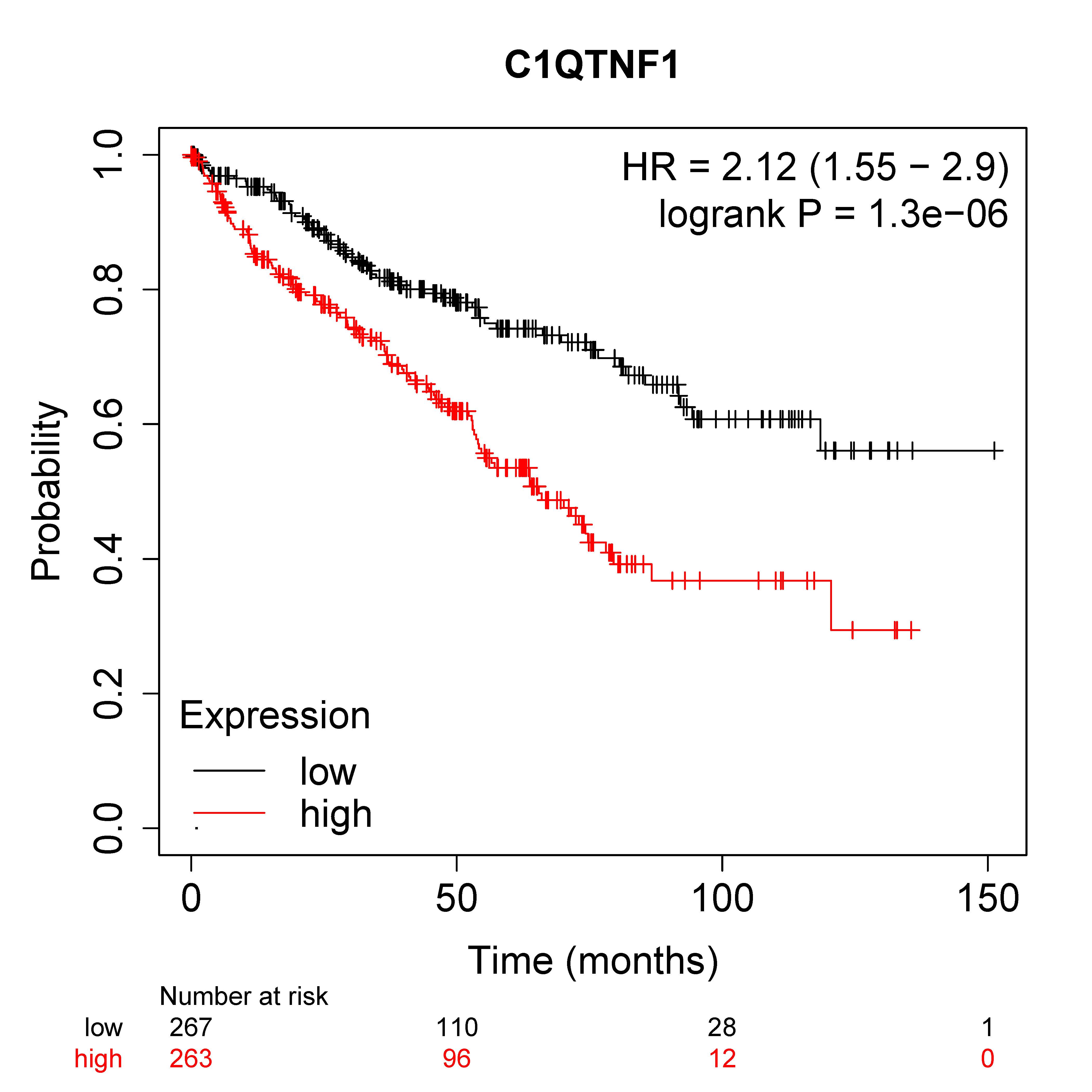

Supplement: Supplementary file 6 [file Image1.JPEG]

Enrichment score of Th17 cells

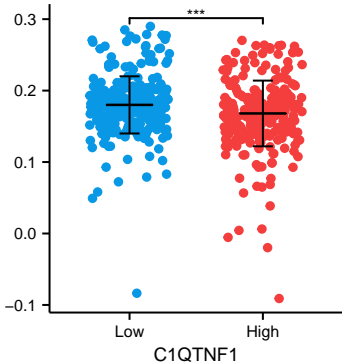

Supplement: Supplementary file 8 [file DataSheet1.ZIP › C1QTNF1 original data 1/Analysis of immune infiltration/免疫浸润_分组比较图_2022-11-12_00_40_04.pdf]

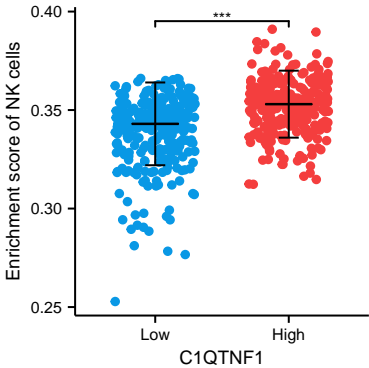

Supplement: Supplementary file 8 [file DataSheet1.ZIP › C1QTNF1 original data 1/Analysis of immune infiltration/免疫浸润_分组比较图_2022-11-12_00_40_17.pdf]

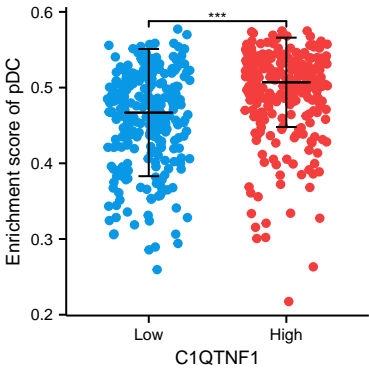

Supplement: Supplementary file 8 [file DataSheet1.ZIP › C1QTNF1 original data 1/Analysis of immune infiltration/免疫浸润_分组比较图_2022-11-12_00_40_26.pdf]

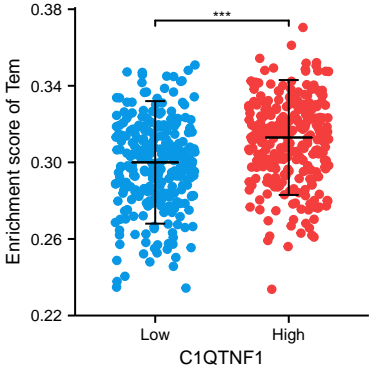

Supplement: Supplementary file 8 [file DataSheet1.ZIP › C1QTNF1 original data 1/Analysis of immune infiltration/免疫浸润_分组比较图_2022-11-12_00_40_52.pdf]

Correlation

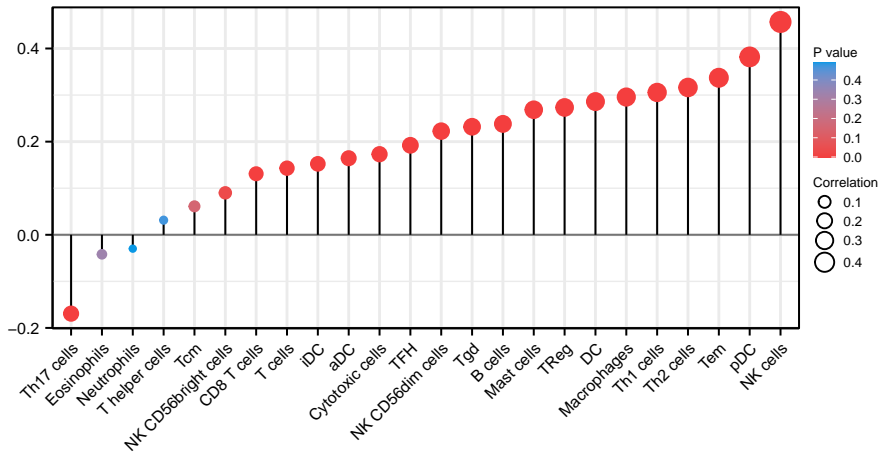

Supplement: Supplementary file 8 [file DataSheet1.ZIP › C1QTNF1 original data 1/Analysis of immune infiltration/免疫浸润_棒棒糖图_2022-11-12_00_38_07.pdf]

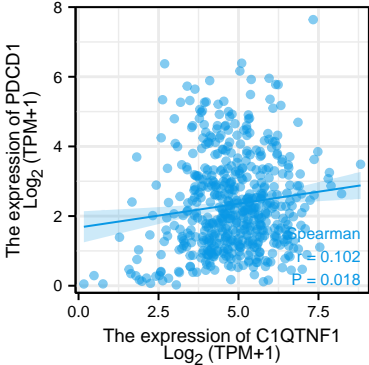

Supplement: Supplementary file 8 [file DataSheet1.ZIP › C1QTNF1 original data 1/Analysis of immune infiltration/分子相关性分析_2022-11-12_00_41_24.pdf]

The expression of CTLA4  
 $\text{Log}_2(\text{TPM}+1)$

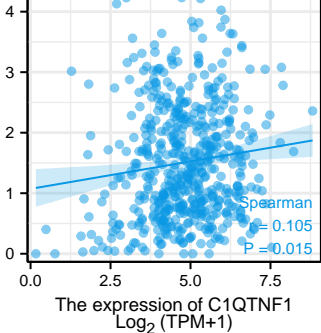

Supplement: Supplementary file 8 [file DataSheet1.ZIP › C1QTNF1 original data 1/Analysis of immune infiltration/分子相关性分析_2022-11-12_00_41_37.pdf]

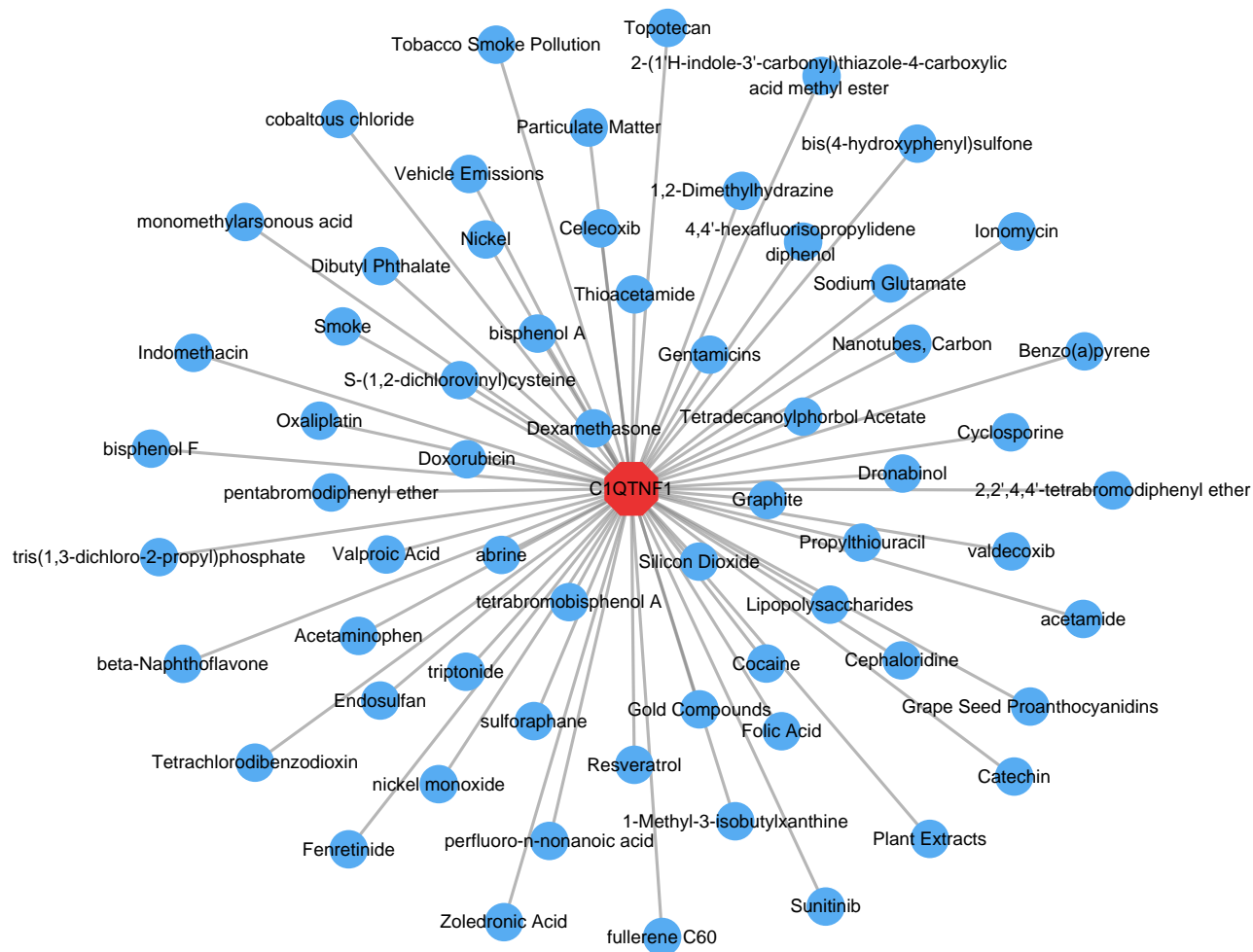

Supplement: Supplementary file 8 [file DataSheet1.ZIP › C1QTNF1 original data 1/Chemical Interaction Analysis (CTD)/药物网络.pdf]

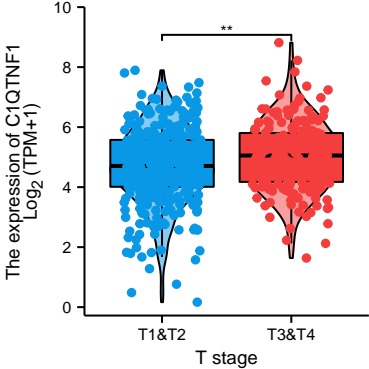

Supplement: Supplementary file 8 [file DataSheet1.ZIP › C1QTNF1 original data 1/Clinical relevance ananlysis/临床相关性_2022-11-11_23_44_18.pdf]

The expression of C1QTNF1  
 $\text{Log}_2(\text{TPM}+1)$

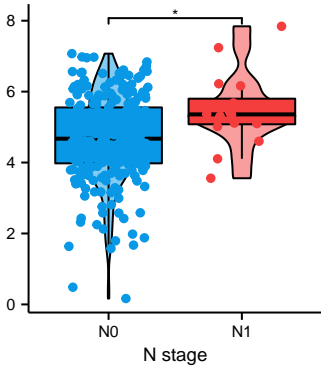

Supplement: Supplementary file 8 [file DataSheet1.ZIP › C1QTNF1 original data 1/Clinical relevance ananlysis/临床相关性_2022-11-11_23_45_14.pdf]

The expression of C1QTNF1  
 $\text{Log}_2(\text{TPM}+1)$

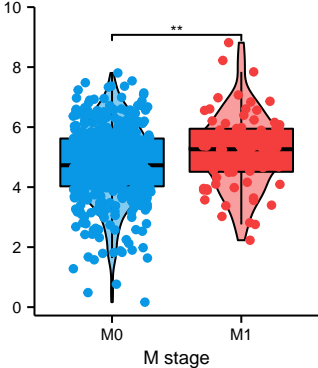

Supplement: Supplementary file 8 [file DataSheet1.ZIP › C1QTNF1 original data 1/Clinical relevance ananlysis/临床相关性_2022-11-11_23_45_35.pdf]

The expression of C1QTNF1  
 $\text{Log}_2(\text{TPM}+1)$

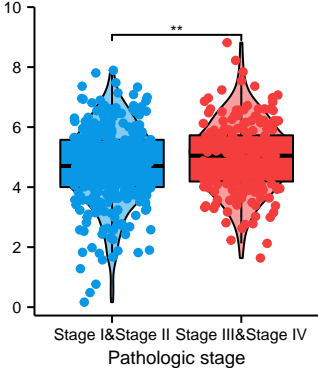

Supplement: Supplementary file 8 [file DataSheet1.ZIP › C1QTNF1 original data 1/Clinical relevance ananlysis/临床相关性_2022-11-11_23_45_50.pdf]

The expression of C1QTNF1  
 $\text{Log}_2(\text{TPM}+1)$

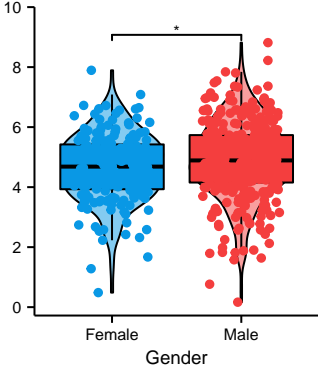

Supplement: Supplementary file 8 [file DataSheet1.ZIP › C1QTNF1 original data 1/Clinical relevance ananlysis/临床相关性_2022-11-11_23_46_09.pdf]

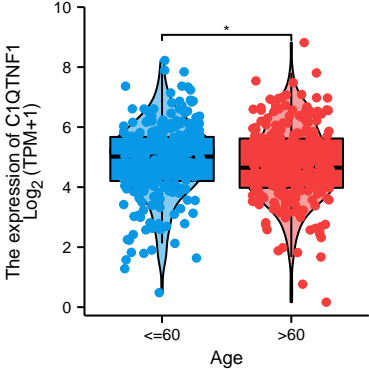

Supplement: Supplementary file 8 [file DataSheet1.ZIP › C1QTNF1 original data 1/Clinical relevance ananlysis/临床相关性_2022-11-11_23_46_35.pdf]

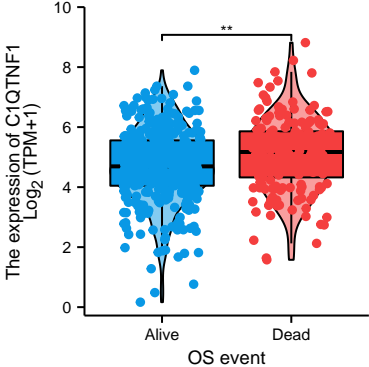

Supplement: Supplementary file 8 [file DataSheet1.ZIP › C1QTNF1 original data 1/Clinical relevance ananlysis/临床相关性_2022-11-11_23_46_56.pdf]

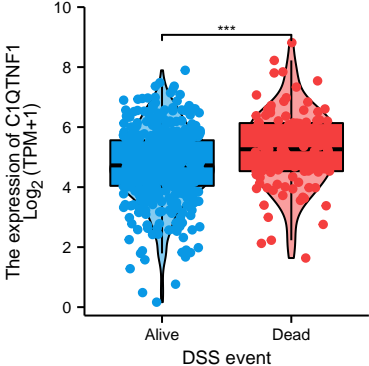

Supplement: Supplementary file 8 [file DataSheet1.ZIP › C1QTNF1 original data 1/Clinical relevance ananlysis/临床相关性_2022-11-11_23_47_10.pdf]

The expression of C1QTNF1  
 $\text{Log}_2(\text{TPM}+1)$

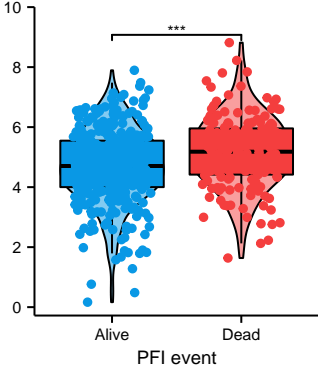

Supplement: Supplementary file 8 [file DataSheet1.ZIP › C1QTNF1 original data 1/Clinical relevance ananlysis/临床相关性_2022-11-11_23_47_25.pdf]

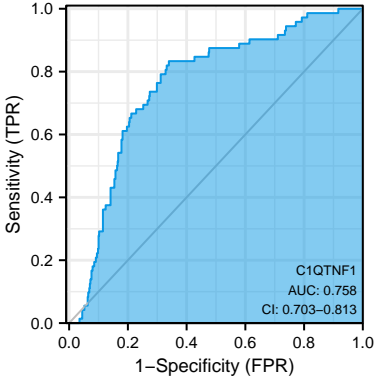

Supplement: Supplementary file 8 [file DataSheet1.ZIP › C1QTNF1 original data 1/Expression difference and diagnostic analysis of KIRC/ROC曲线_2022-11-11_16_18_29.pdf]

The expression of C1QTNF1  
 $\text{Log}_2(\text{TPM}+1)$

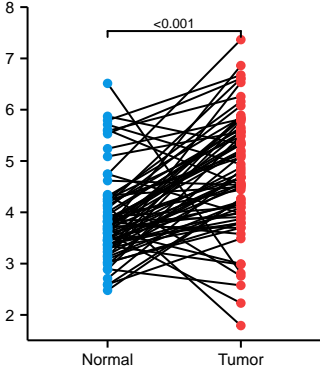

Supplement: Supplementary file 8 [file DataSheet1.ZIP › C1QTNF1 original data 1/Expression difference and diagnostic analysis of KIRC/表达差异_配对样本_2022-11-11_16_15_49.pdf]

The expression of C1QTNF1  
 $\text{Log}_2(\text{TPM}+1)$

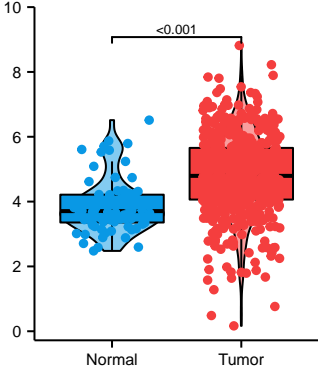

Supplement: Supplementary file 8 [file DataSheet1.ZIP › C1QTNF1 original data 1/Expression difference and diagnostic analysis of KIRC/表达差异_非配对样本_2022-11-11_16_17_27.pdf]

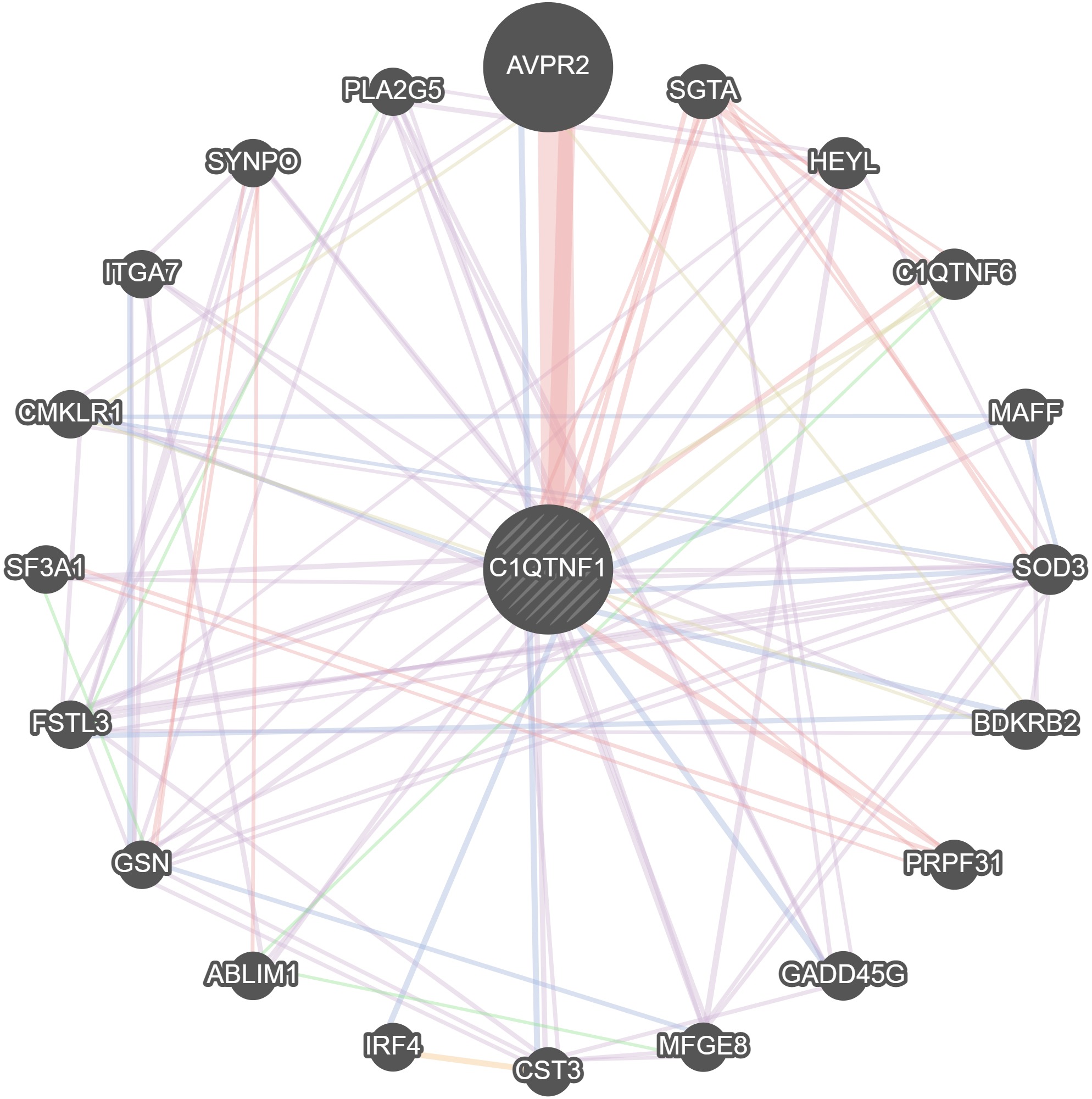

Supplement: Supplementary file 8 [file DataSheet1.ZIP › C1QTNF1 original data 1/geneMINA/genemania-network.jpg]

LogFC

Up  
Down

Z-score

2  
0  
-2

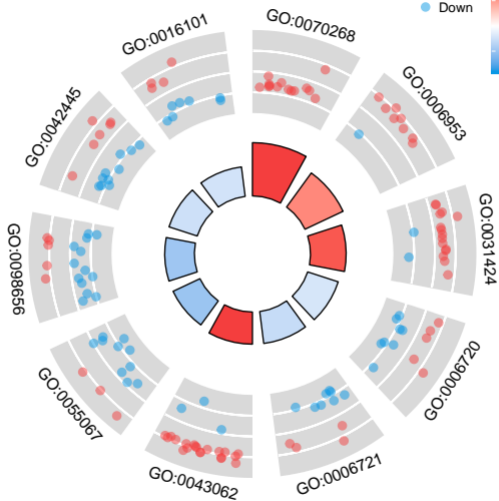

Supplement: Supplementary file 8 [file DataSheet1.ZIP › C1QTNF1 original data 1/GO and KEGG enrichment analysis/GOKEGG圈图_BP.pdf]

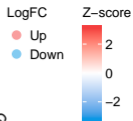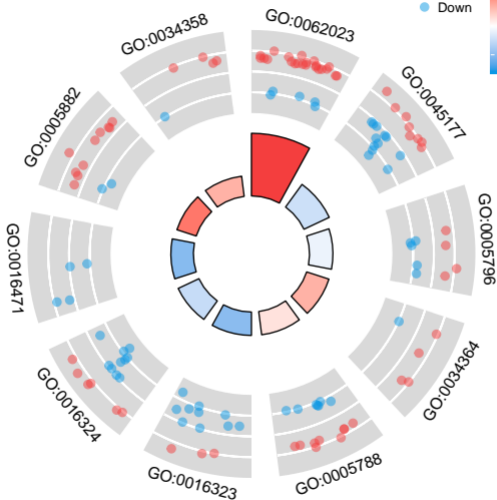

Supplement: Supplementary file 8 [file DataSheet1.ZIP › C1QTNF1 original data 1/GO and KEGG enrichment analysis/GOKEGG圈图_CC.pdf]

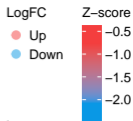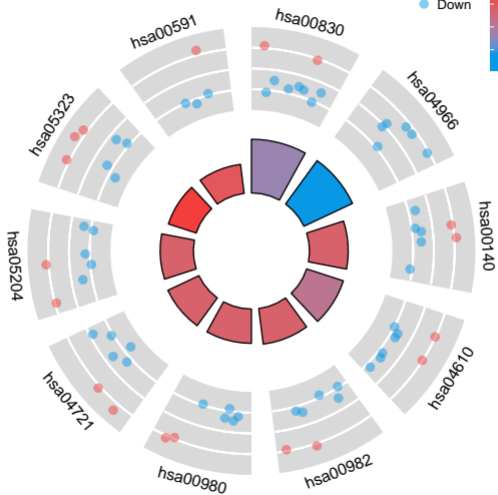

Supplement: Supplementary file 8 [file DataSheet1.ZIP › C1QTNF1 original data 1/GO and KEGG enrichment analysis/GOKEGG圈图_KEGG.pdf]

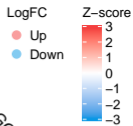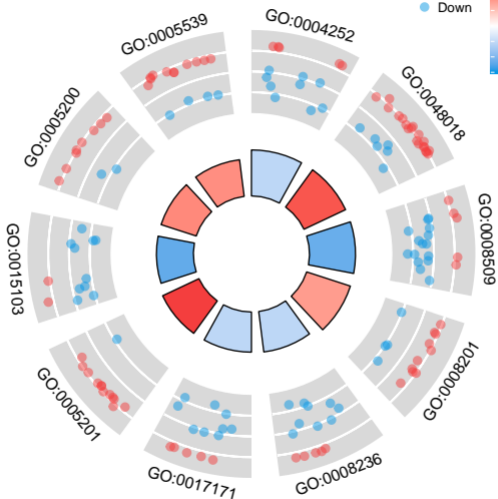

Supplement: Supplementary file 8 [file DataSheet1.ZIP › C1QTNF1 original data 1/GO and KEGG enrichment analysis/GOKEGG圈图_MF.pdf]

# hsa-miR-27b-3p vs. AC010980.2, 517 samples (KIRC)

Data Source: ENROCI project

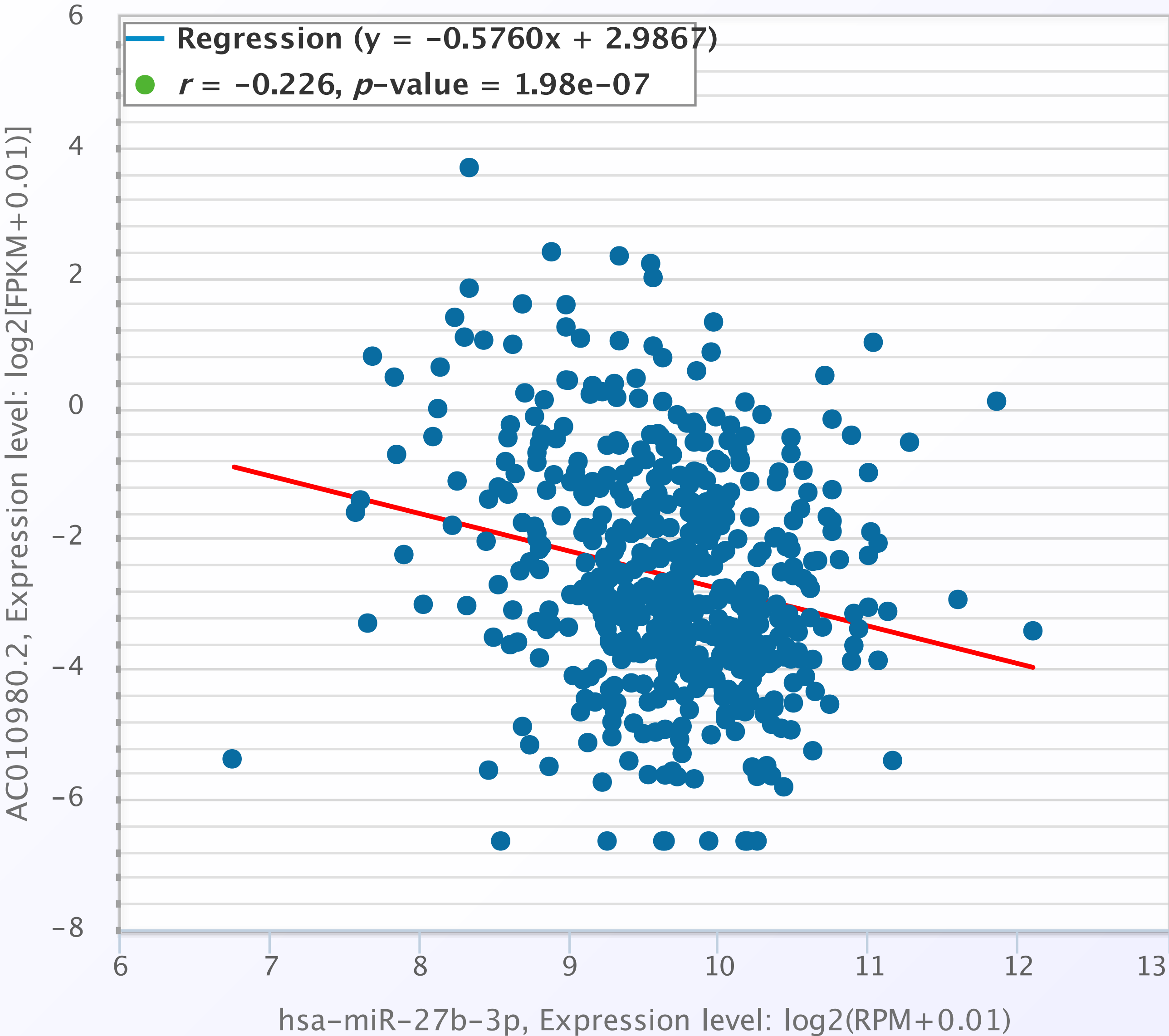

Supplement: Supplementary file 8 [file DataSheet1.ZIP › C1QTNF1 original data 1/LncRNA/ENCORI-hsa-miR-27b-3p_and_AC010980.2_coExp_in_miRNA-RNA_scatter_log2.pdf]

# hsa-miR-27b-3p vs. AC016717.2, 517 samples (KIRC)

Data Source: ENROCI project

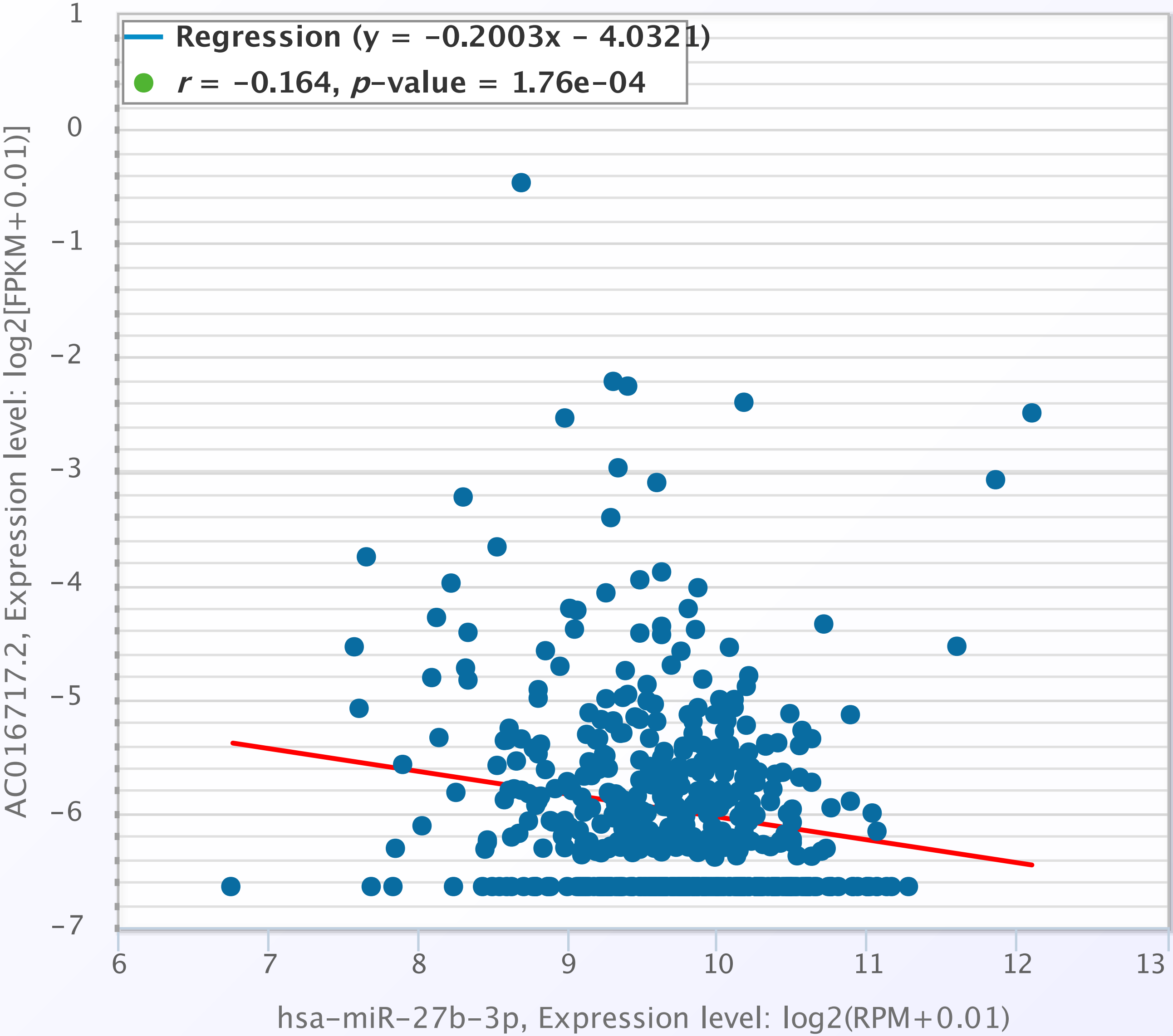

Supplement: Supplementary file 8 [file DataSheet1.ZIP › C1QTNF1 original data 1/LncRNA/ENCORI-hsa-miR-27b-3p_and_AC016717.2_coExp_in_miRNA-RNA_scatter_log2.pdf]

# hsa-miR-27b-3p vs. AC040970.1, 517 samples (KIRC)

Data Source: ENROCI project

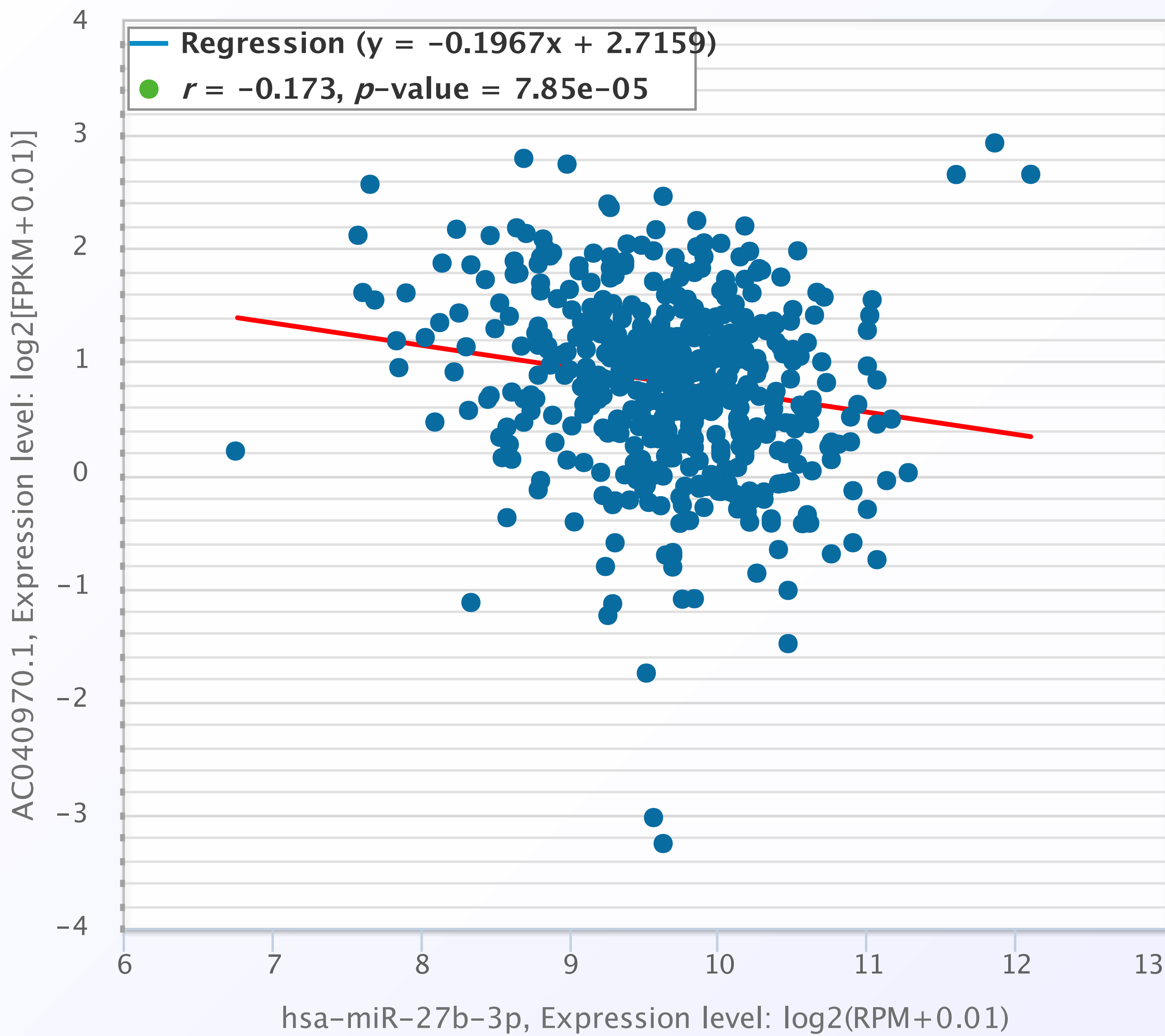

Supplement: Supplementary file 8 [file DataSheet1.ZIP › C1QTNF1 original data 1/LncRNA/ENCORI-hsa-miR-27b-3p_and_AC040970.1_coExp_in_miRNA-RNA_scatter_log2.pdf]

# hsa-miR-27b-3p vs. AC239868.1, 517 samples (KIRC)

Data Source: ENROCI project

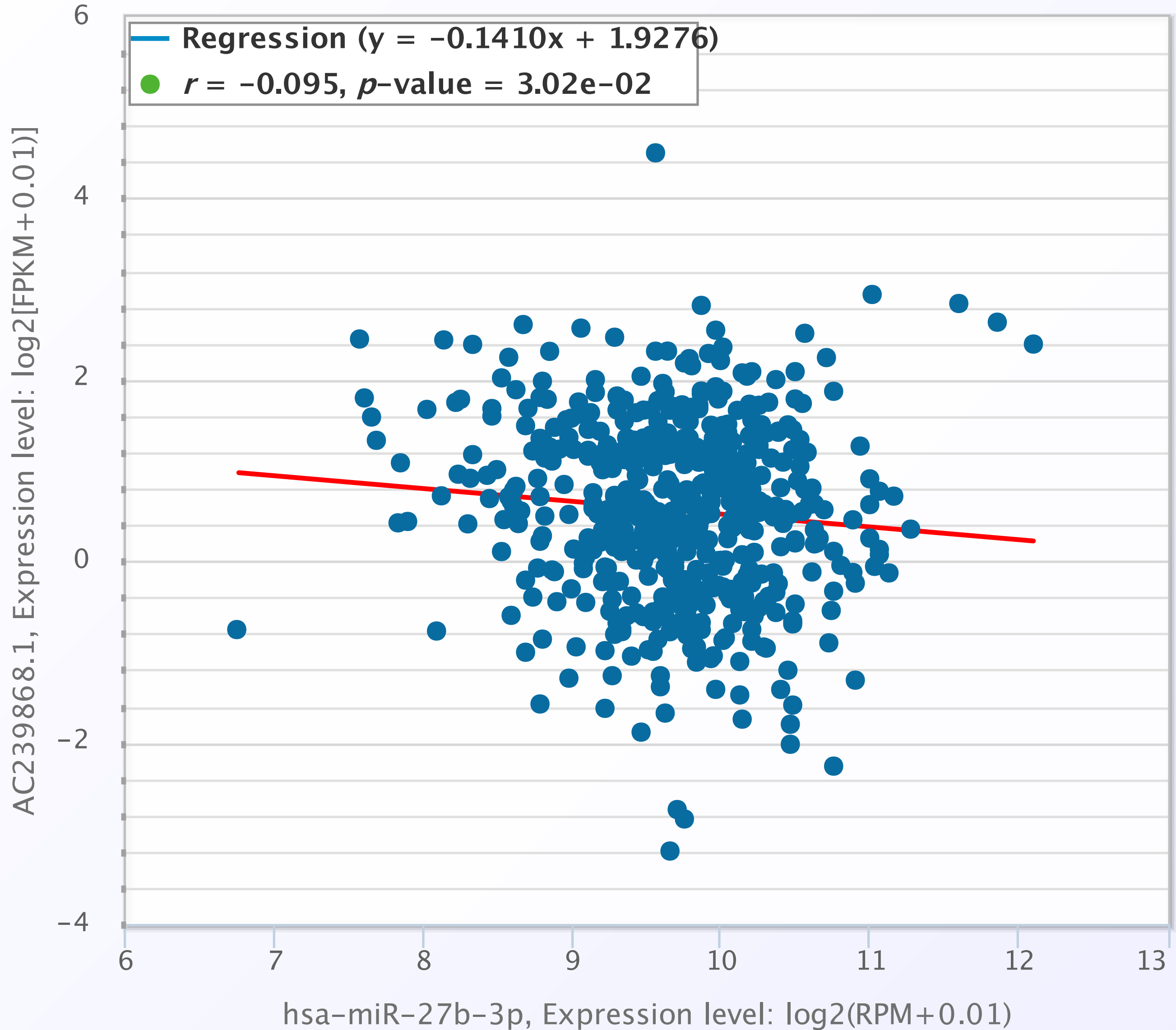

Supplement: Supplementary file 8 [file DataSheet1.ZIP › C1QTNF1 original data 1/LncRNA/ENCORI-hsa-miR-27b-3p_and_AC239868.1_coExp_in_miRNA-RNA_scatter_log2.pdf]

# hsa-miR-27b-3p vs. CASC19, 517 samples (KIRC)

Data Source: ENROCI project

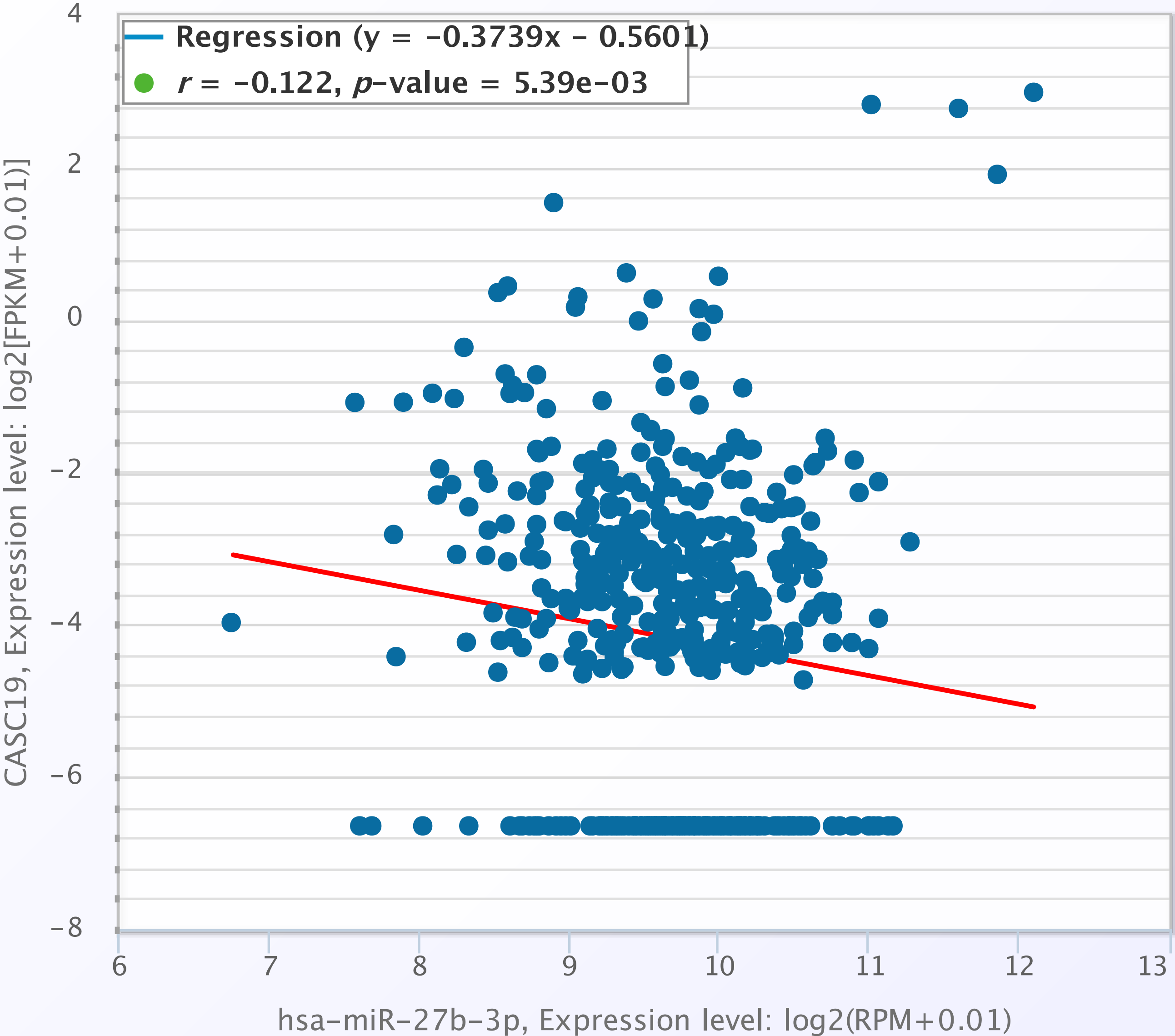

Supplement: Supplementary file 8 [file DataSheet1.ZIP › C1QTNF1 original data 1/LncRNA/ENCORI-hsa-miR-27b-3p_and_CASC19_coExp_in_miRNA-RNA_scatter_log2.pdf]

# hsa-miR-27b-3p vs. CYTOR, 517 samples (KIRC)

Data Source: ENROCI project

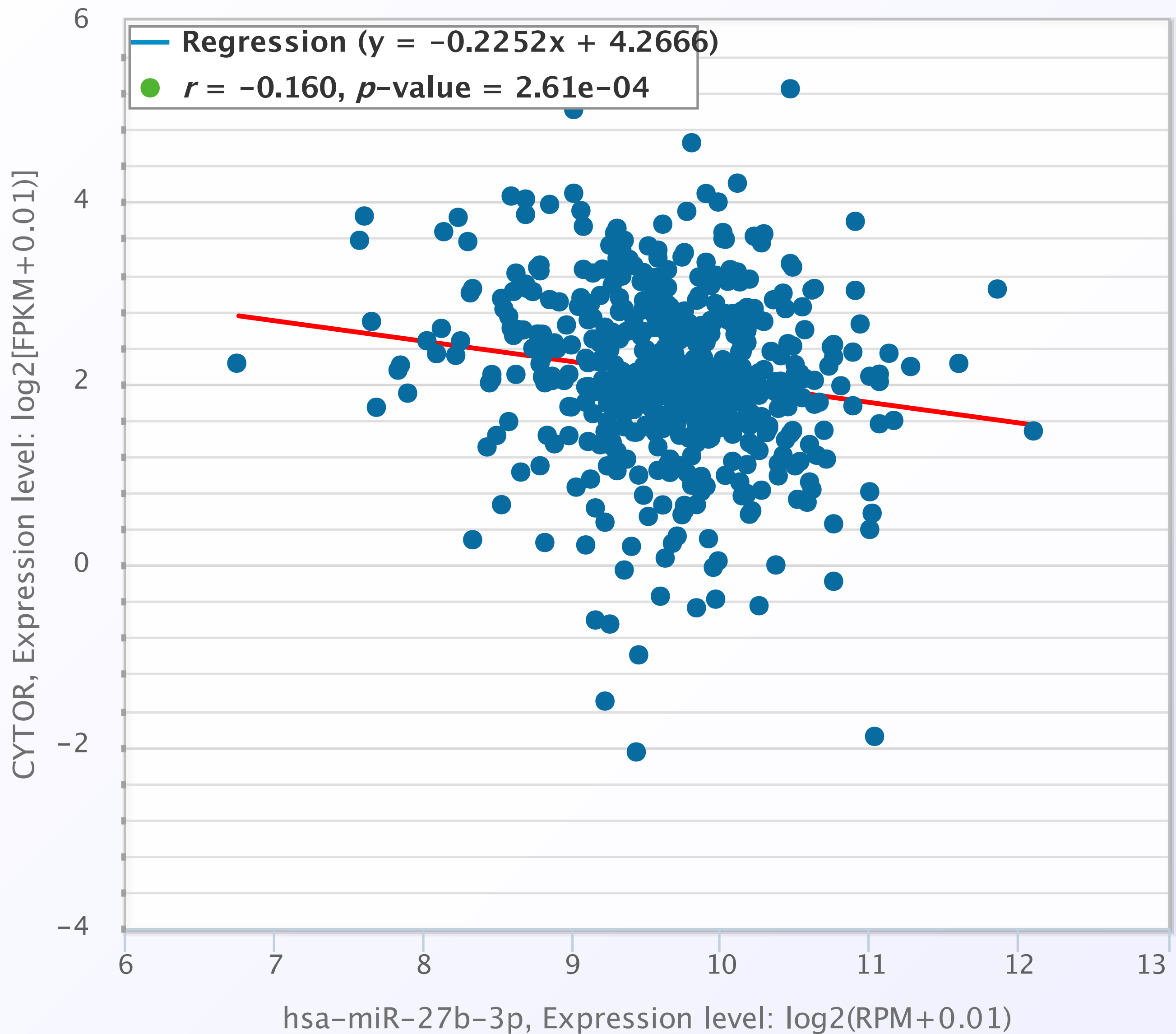

Supplement: Supplementary file 8 [file DataSheet1.ZIP › C1QTNF1 original data 1/LncRNA/ENCORI-hsa-miR-27b-3p_and_CYTOR_coExp_in_miRNA-RNA_scatter_log2.pdf]

# hsa-miR-27b-3p vs. LINC02381, 517 samples (KIRC)

Data Source: ENROCI project

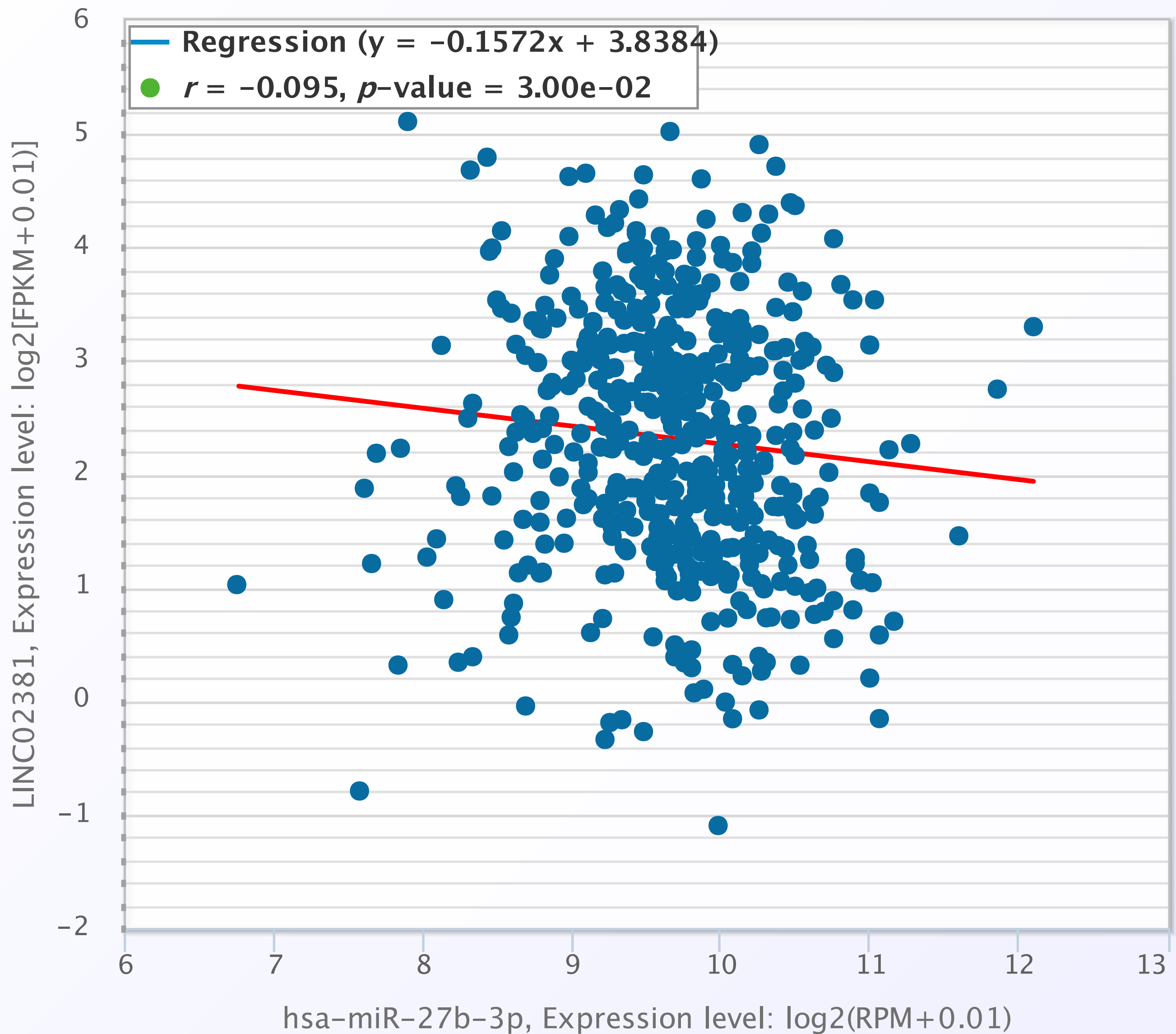

Supplement: Supplementary file 8 [file DataSheet1.ZIP › C1QTNF1 original data 1/LncRNA/ENCORI-hsa-miR-27b-3p_and_LINC02381_coExp_in_miRNA-RNA_scatter_log2.pdf]

# AC010980.2 vs. C1QTNF1, 535 samples (KIRC)

Data Source: ENROCI project

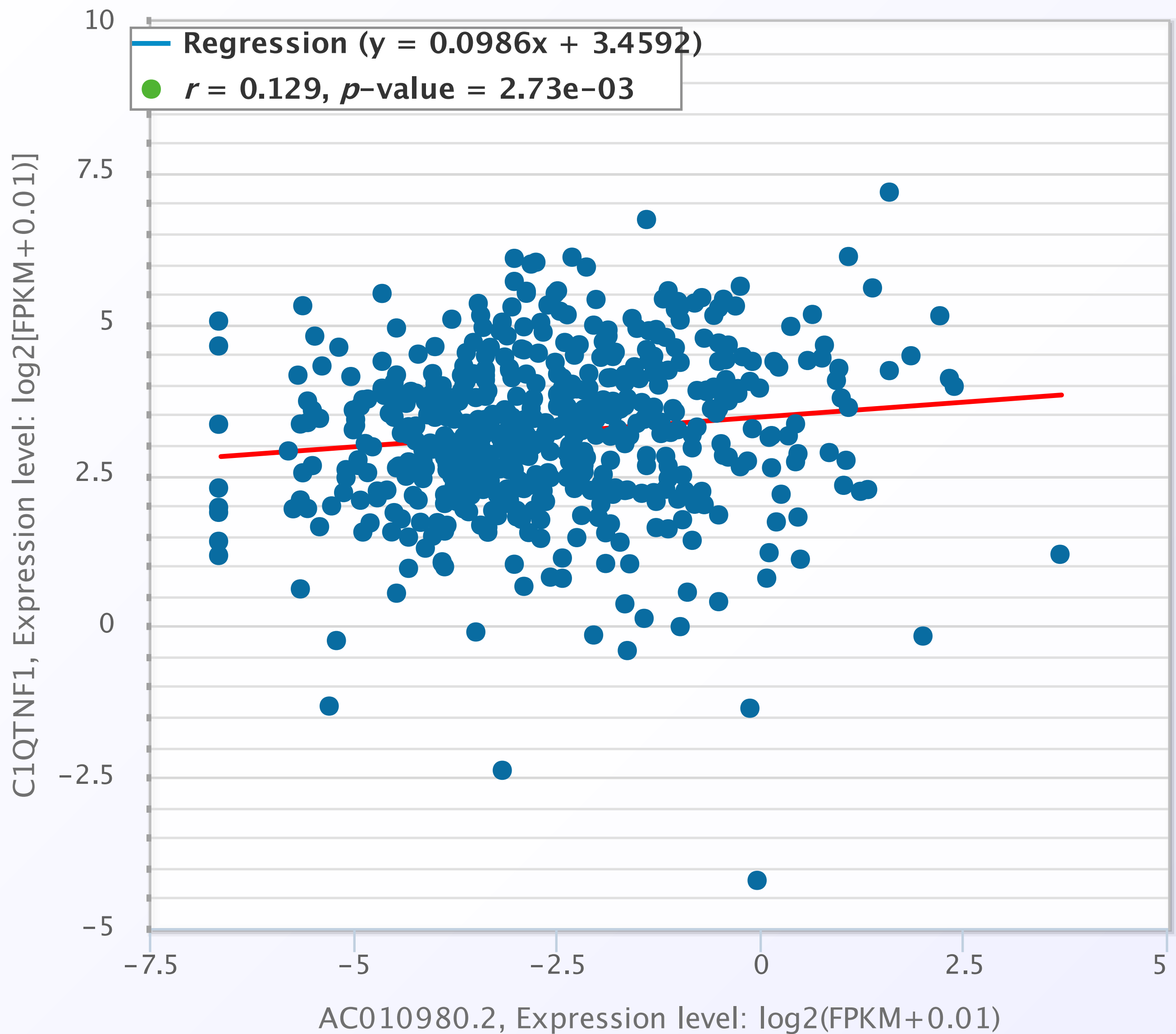

Supplement: Supplementary file 8 [file DataSheet1.ZIP › C1QTNF1 original data 1/LncRNA/lincRNA与C1QTNF1/ENCORI-AC010980.2_and_C1QTNF1_coExp_in_RNA-RNA_scatter_log2.pdf]

# AC016717.2 vs. C1QTNF1, 535 samples (KIRC)

Data Source: ENROCI project

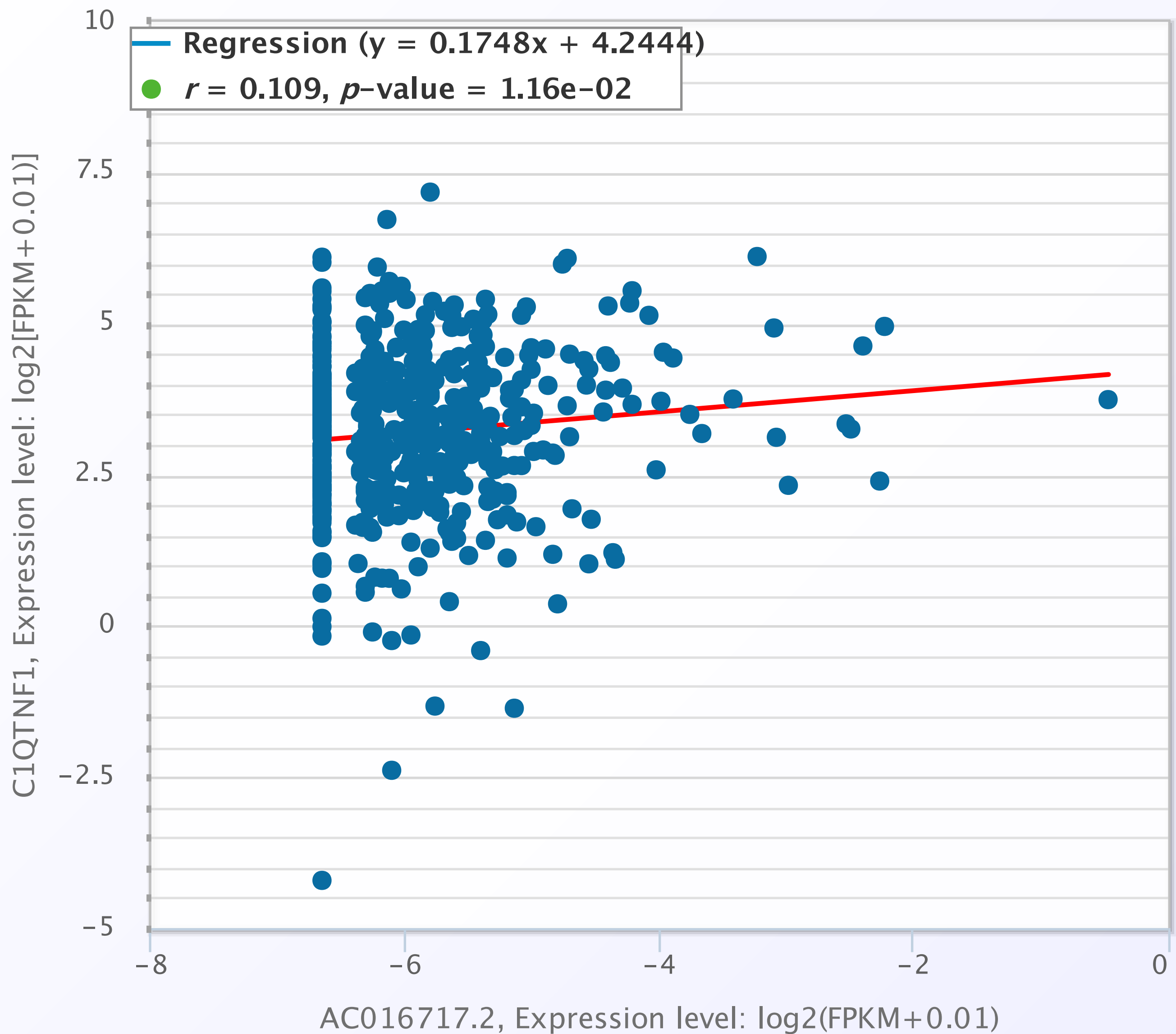

Supplement: Supplementary file 8 [file DataSheet1.ZIP › C1QTNF1 original data 1/LncRNA/lincRNA与C1QTNF1/ENCORI-AC016717.2_and_C1QTNF1_coExp_in_RNA-RNA_scatter_log2.pdf]

# AC040970.1 vs. C1QTNF1, 535 samples (KIRC)

Data Source: ENROCI project

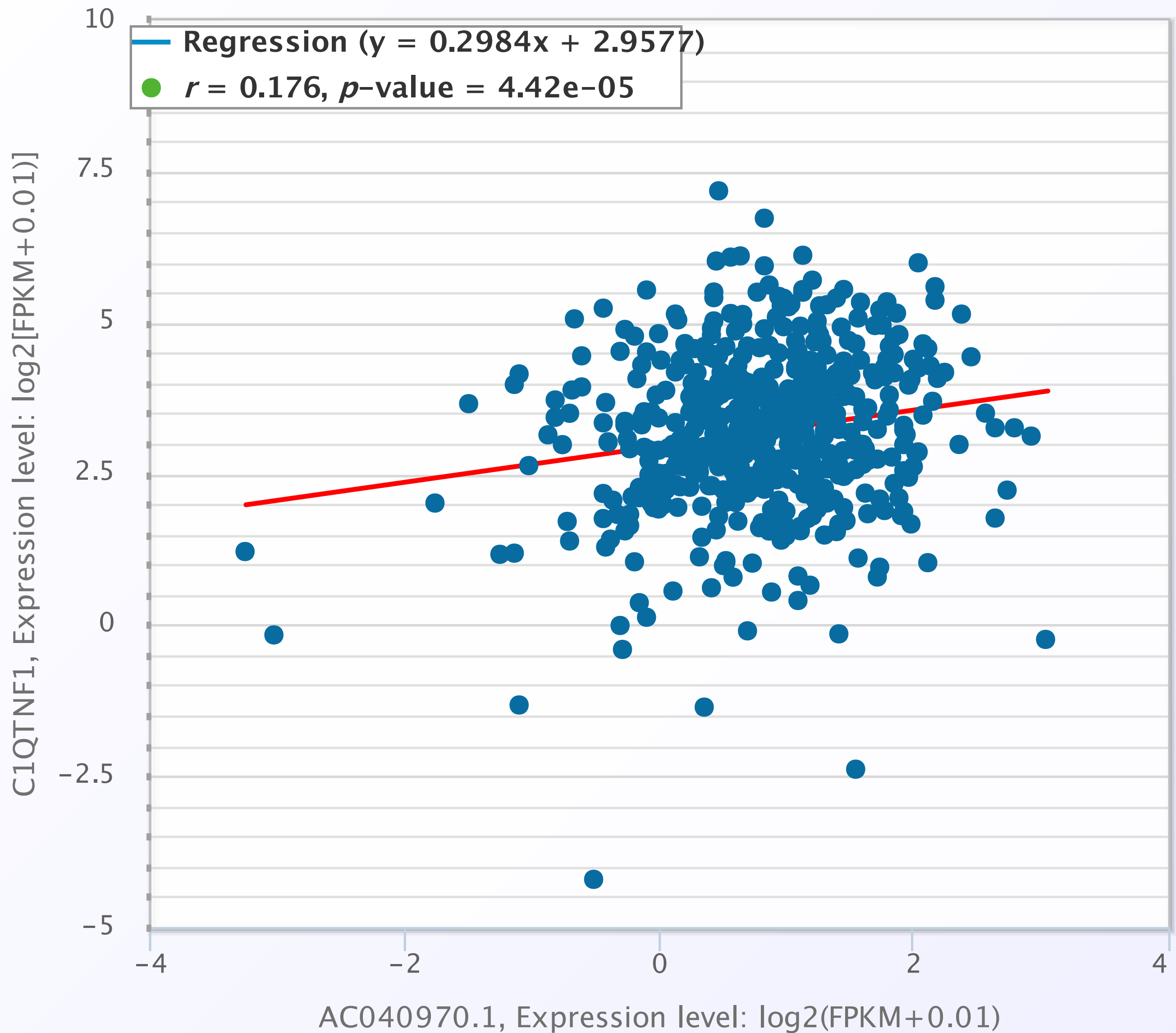

Supplement: Supplementary file 8 [file DataSheet1.ZIP › C1QTNF1 original data 1/LncRNA/lincRNA与C1QTNF1/ENCORI-AC040970.1_and_C1QTNF1_coExp_in_RNA-RNA_scatter_log2.pdf]

# AC239868.1 vs. C1QTNF1, 535 samples (KIRC)

Data Source: ENROCI project

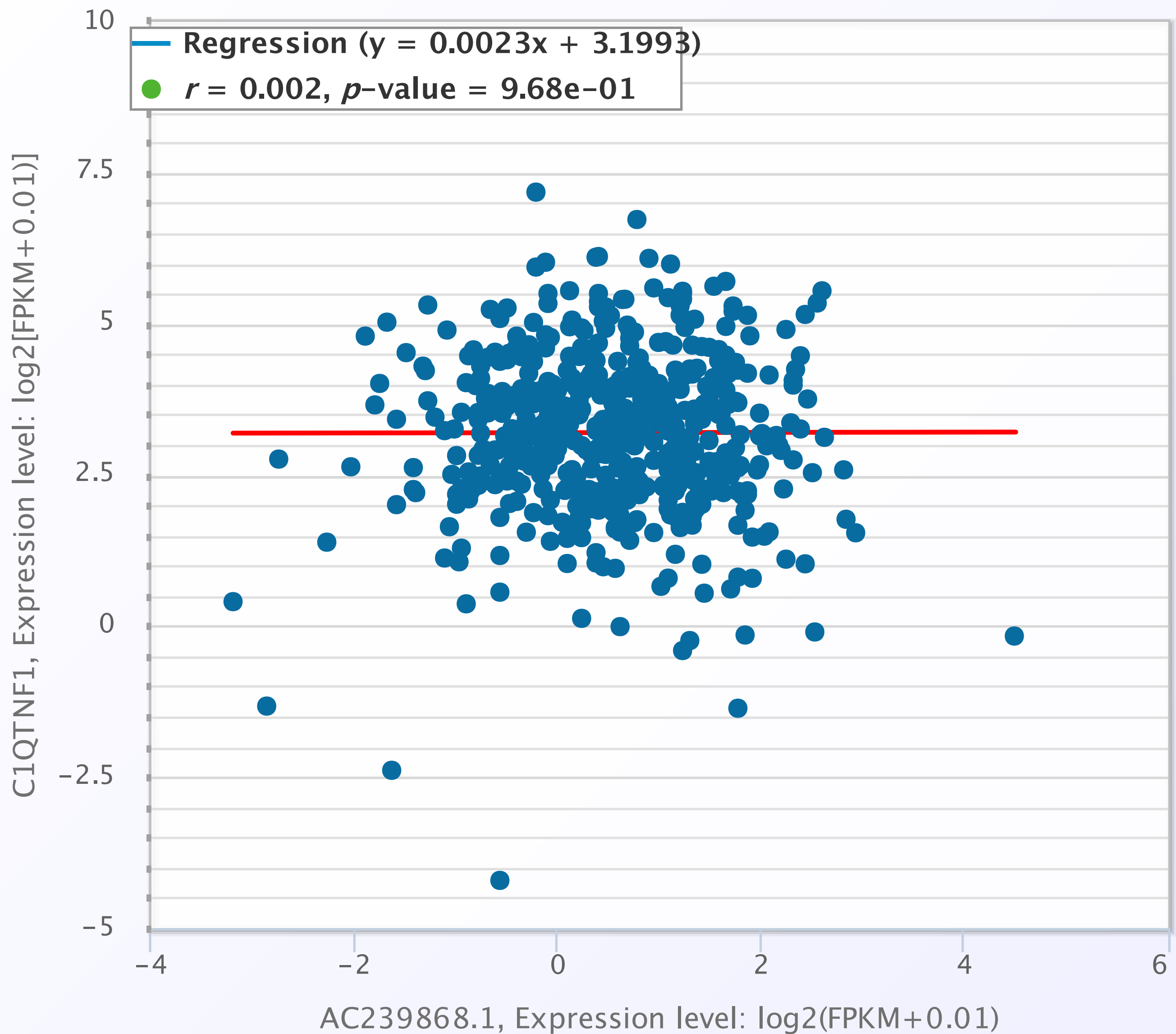

Supplement: Supplementary file 8 [file DataSheet1.ZIP › C1QTNF1 original data 1/LncRNA/lincRNA与C1QTNF1/ENCORI-AC239868.1_and_C1QTNF1_coExp_in_RNA-RNA_scatter_log2.pdf]

# CASC19 vs. C1QTNF1, 535 samples (KIRC)

Data Source: ENROCI project

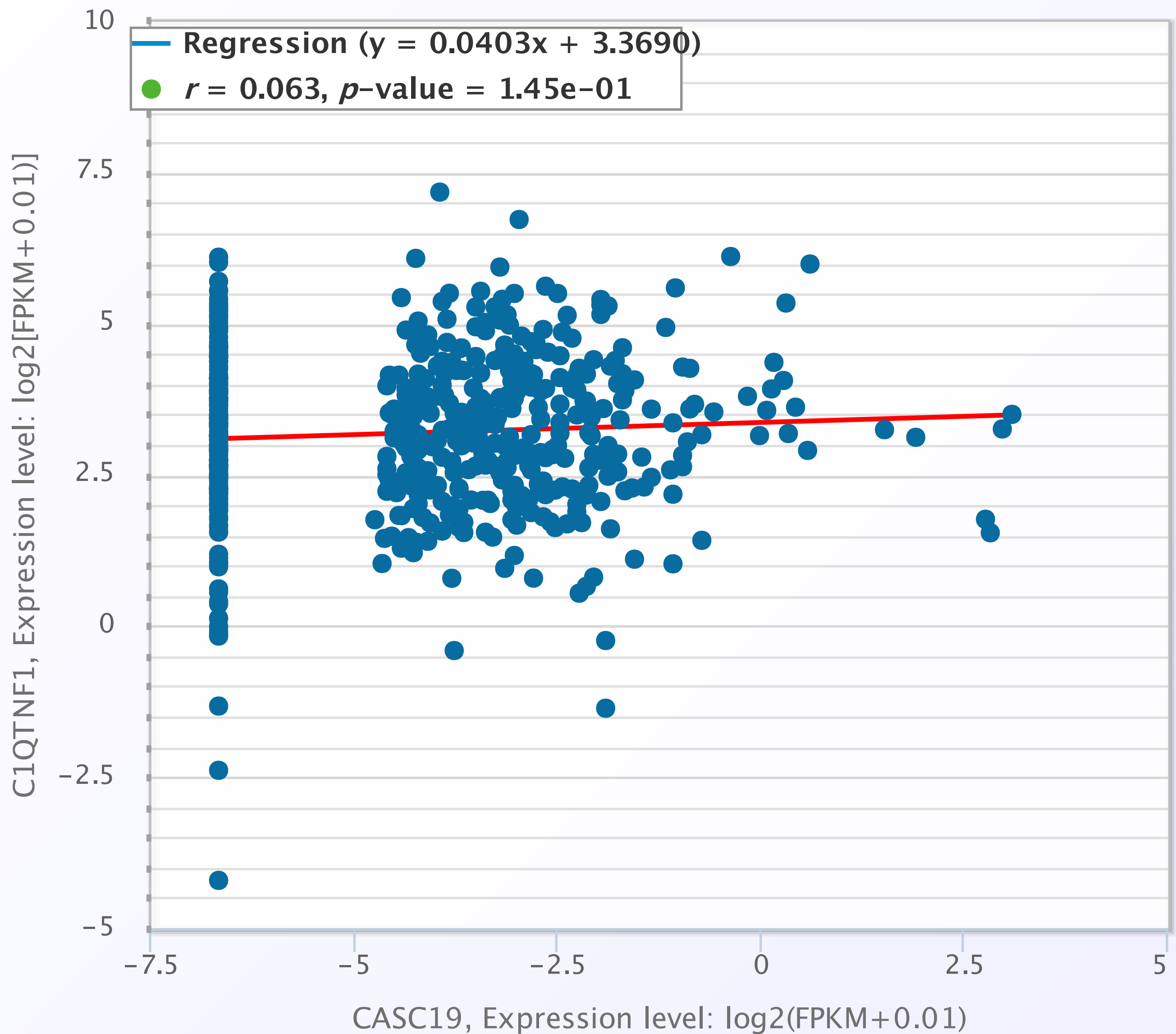

Supplement: Supplementary file 8 [file DataSheet1.ZIP › C1QTNF1 original data 1/LncRNA/lincRNA与C1QTNF1/ENCORI-CASC19_and_C1QTNF1_coExp_in_RNA-RNA_scatter_log2.pdf]

# CYTOR vs. C1QTNF1, 535 samples (KIRC)

Data Source: ENROCI project

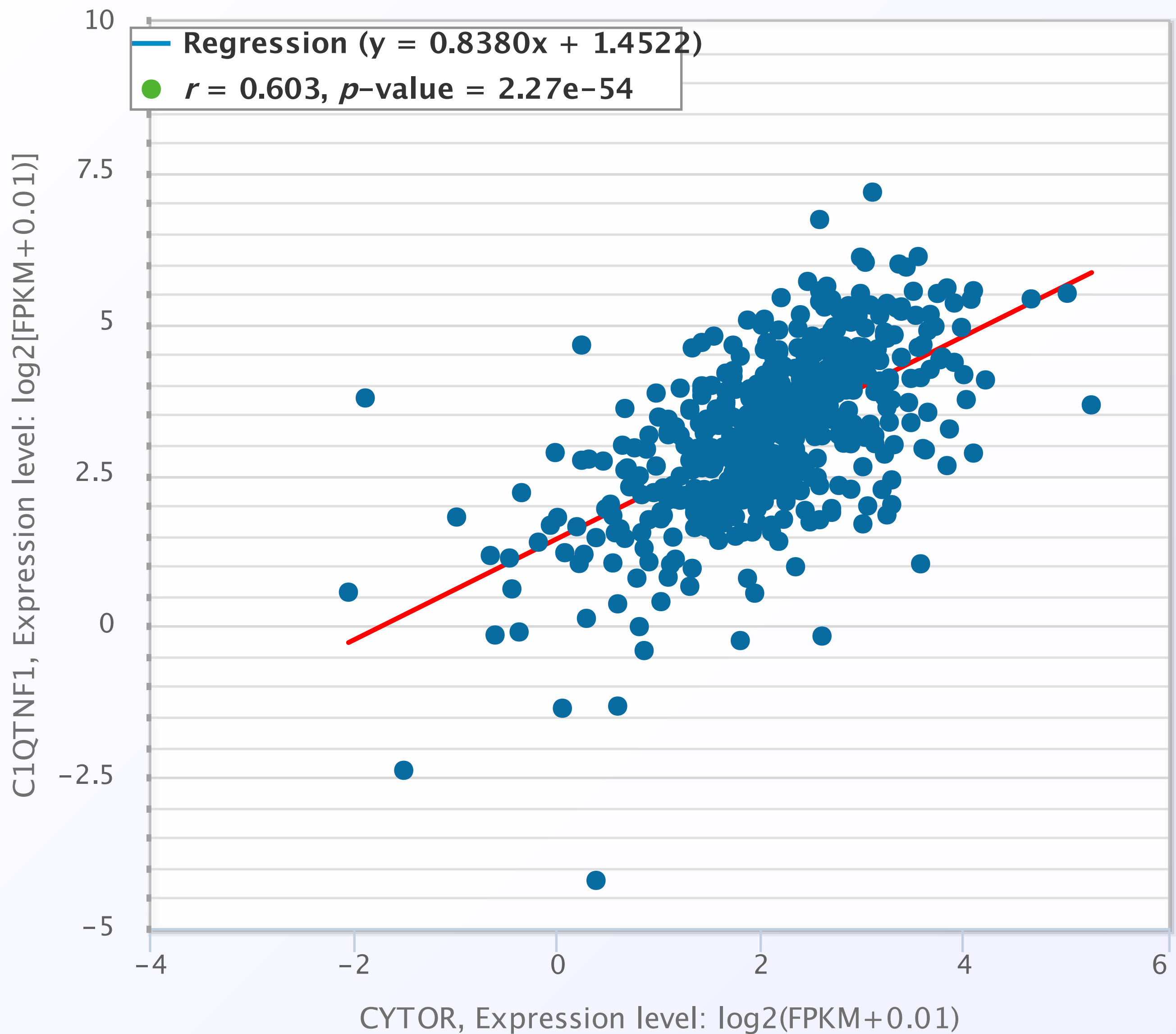

Supplement: Supplementary file 8 [file DataSheet1.ZIP › C1QTNF1 original data 1/LncRNA/lincRNA与C1QTNF1/ENCORI-CYTOR_and_C1QTNF1_coExp_in_RNA-RNA_scatter_log2.pdf]

# LINC02381 vs. C1QTNF1, 535 samples (KIRC)

Data Source: ENROCI project

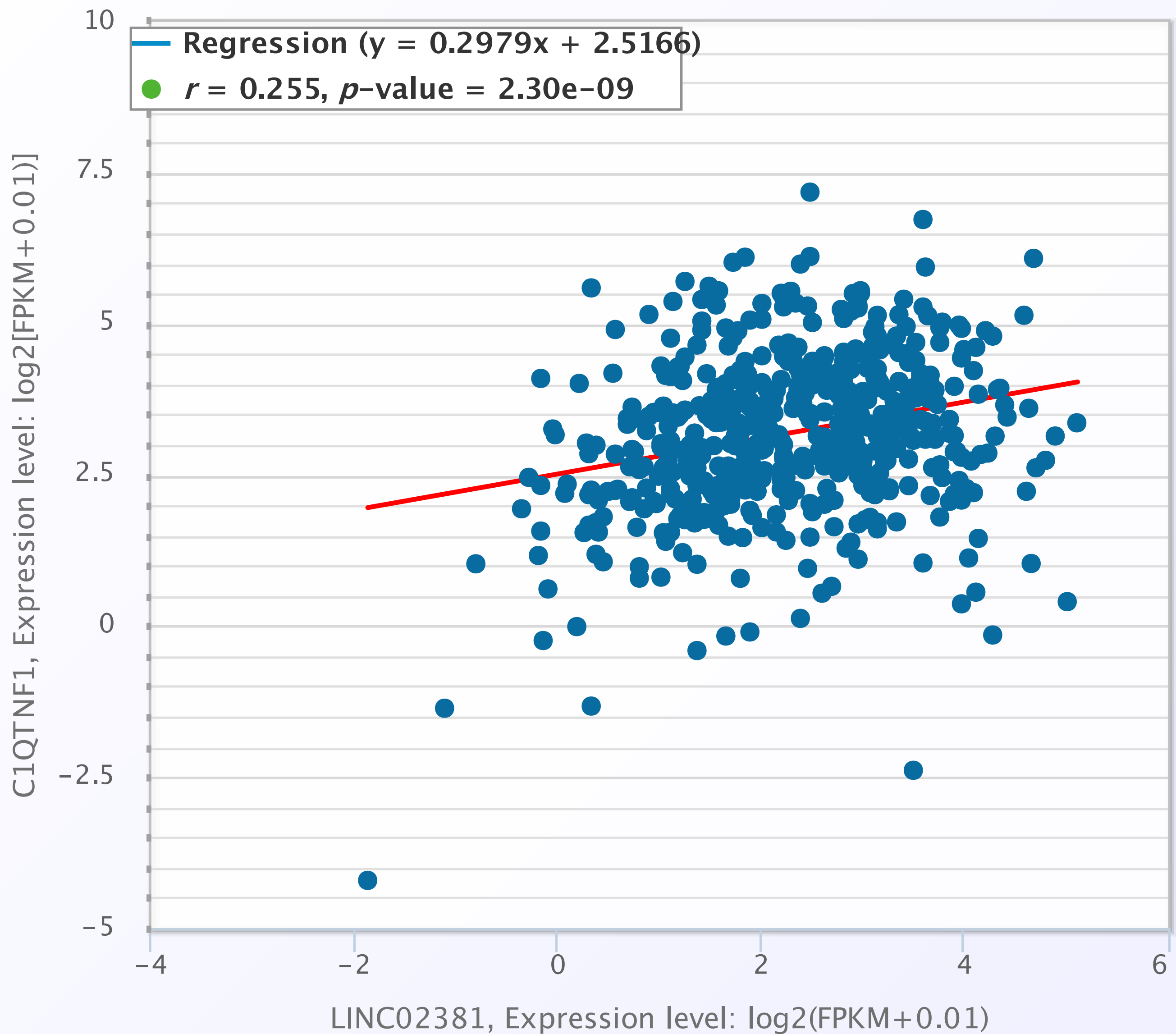

Supplement: Supplementary file 8 [file DataSheet1.ZIP › C1QTNF1 original data 1/LncRNA/lincRNA与C1QTNF1/ENCORI-LINC02381_and_C1QTNF1_coExp_in_RNA-RNA_scatter_log2.pdf]

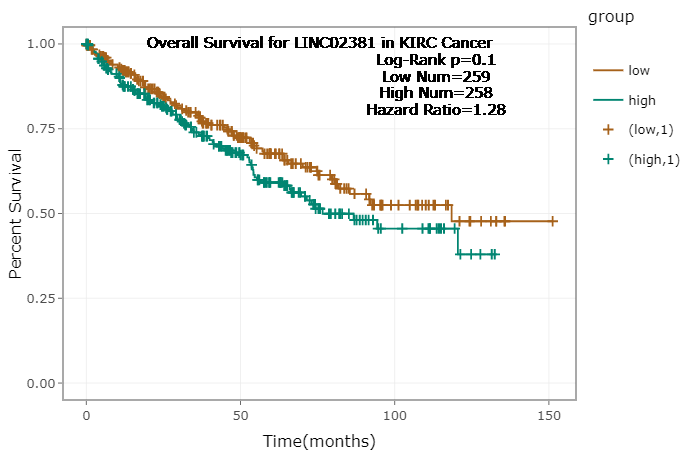

Supplement: Supplementary file 8 [file DataSheet1.ZIP › C1QTNF1 original data 1/LncRNA/lincRNA生存/newplot (1).png]

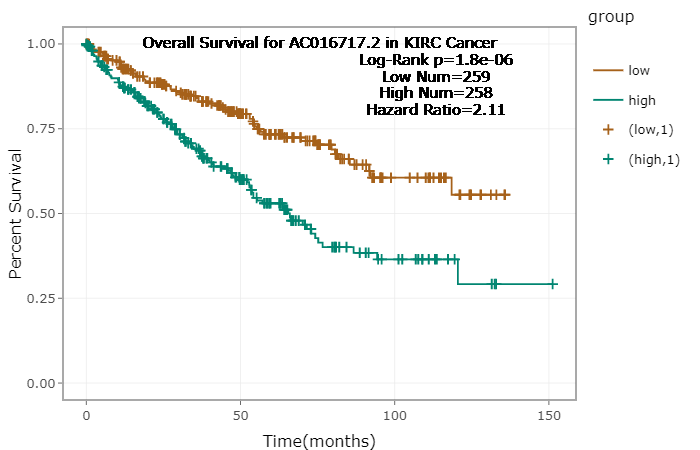

Supplement: Supplementary file 8 [file DataSheet1.ZIP › C1QTNF1 original data 1/LncRNA/lincRNA生存/newplot (2).png]

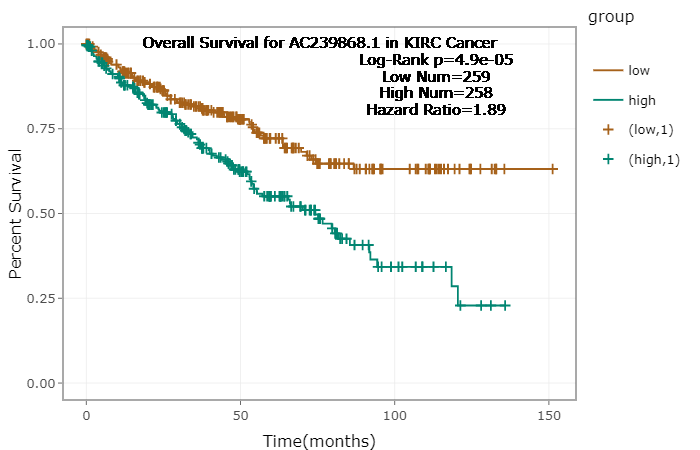

Supplement: Supplementary file 8 [file DataSheet1.ZIP › C1QTNF1 original data 1/LncRNA/lincRNA生存/newplot (3).png]

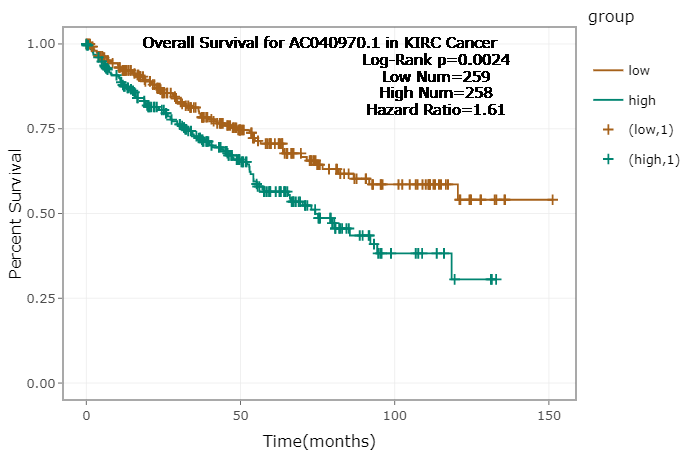

Supplement: Supplementary file 8 [file DataSheet1.ZIP › C1QTNF1 original data 1/LncRNA/lincRNA生存/newplot (4).png]

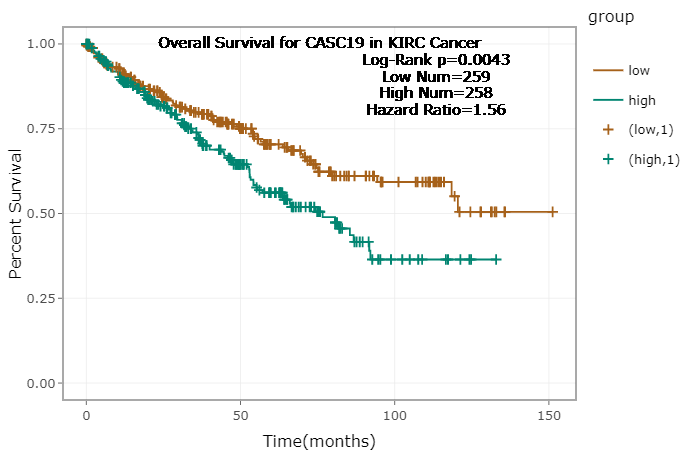

Supplement: Supplementary file 8 [file DataSheet1.ZIP › C1QTNF1 original data 1/LncRNA/lincRNA生存/newplot (5).png]

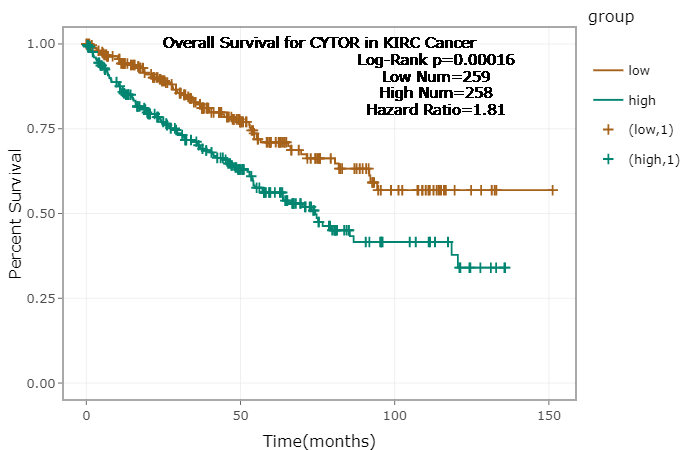

Supplement: Supplementary file 8 [file DataSheet1.ZIP › C1QTNF1 original data 1/LncRNA/lincRNA生存/newplot (6).png]

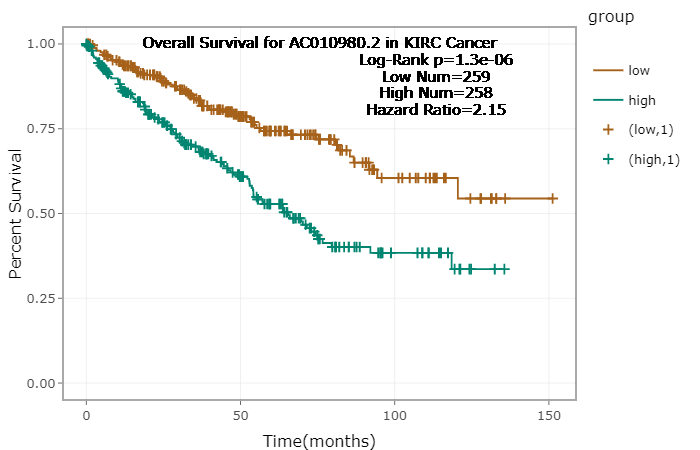

Supplement: Supplementary file 8 [file DataSheet1.ZIP › C1QTNF1 original data 1/LncRNA/lincRNA生存/newplot.png]

# AC010980.2 with 535 cancer and 72 normal samples in KIRC

Data Source: ENROCI project

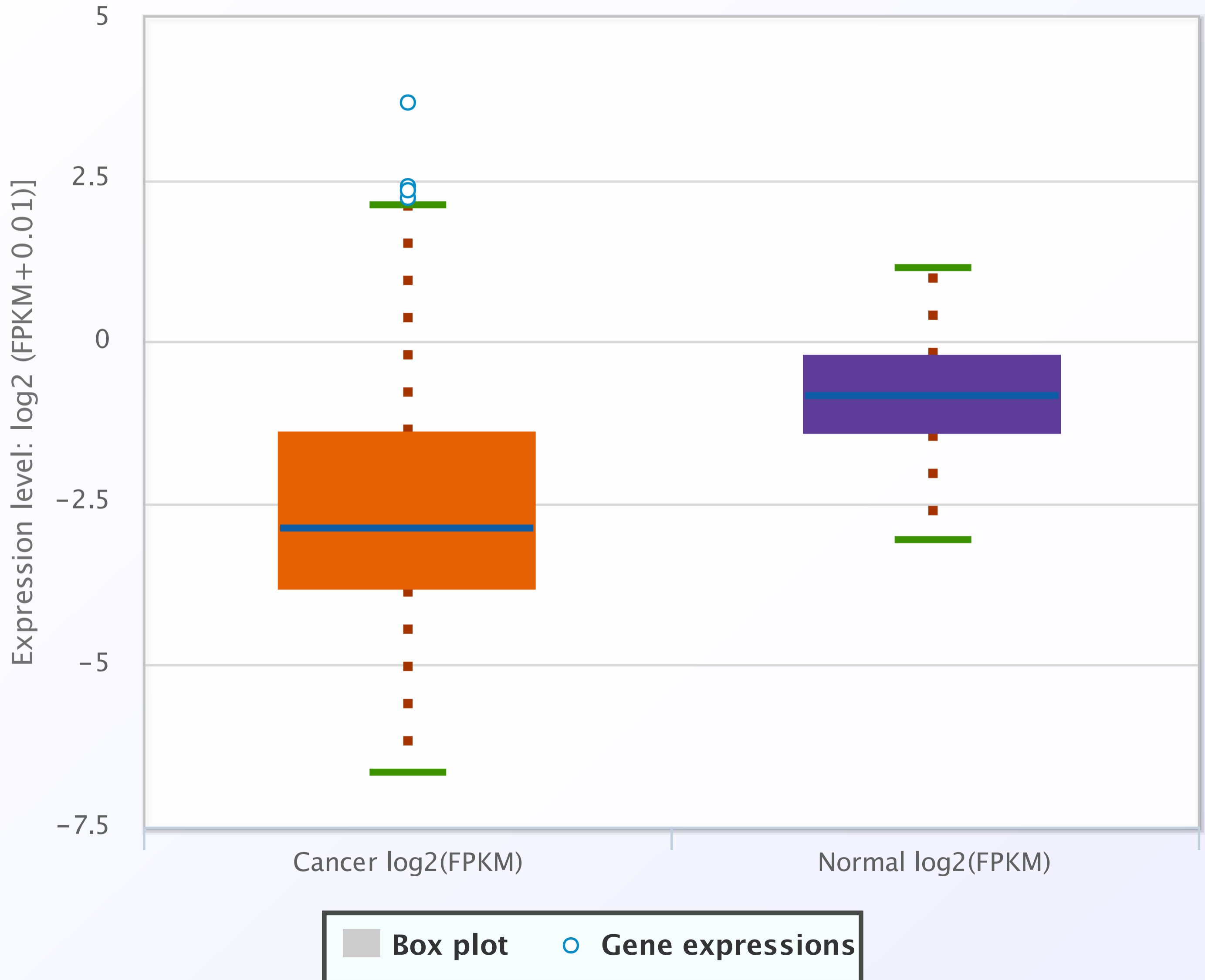

Supplement: Supplementary file 8 [file DataSheet1.ZIP › C1QTNF1 original data 1/LncRNA/lincRNA表达/ENCORI-AC010980.2_in_KIRC_boxplot(log2).pdf]

# AC016717.2 with 535 cancer and 72 normal samples in KIRC

Data Source: ENROCI project

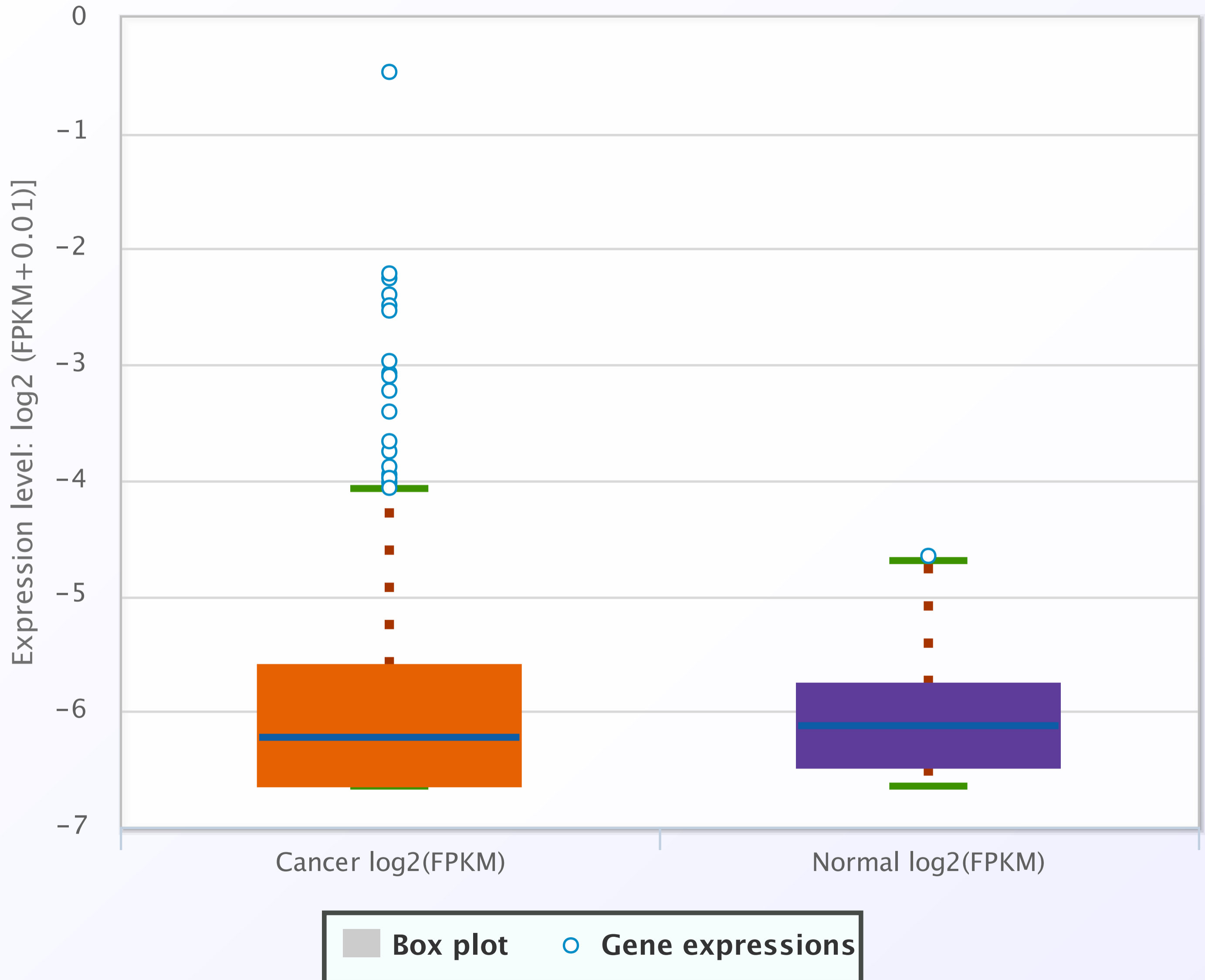

Supplement: Supplementary file 8 [file DataSheet1.ZIP › C1QTNF1 original data 1/LncRNA/lincRNA表达/ENCORI-AC016717.2_in_KIRC_boxplot(log2).pdf]

# AC040970.1 with 535 cancer and 72 normal samples in KIRC

Data Source: ENROCI project

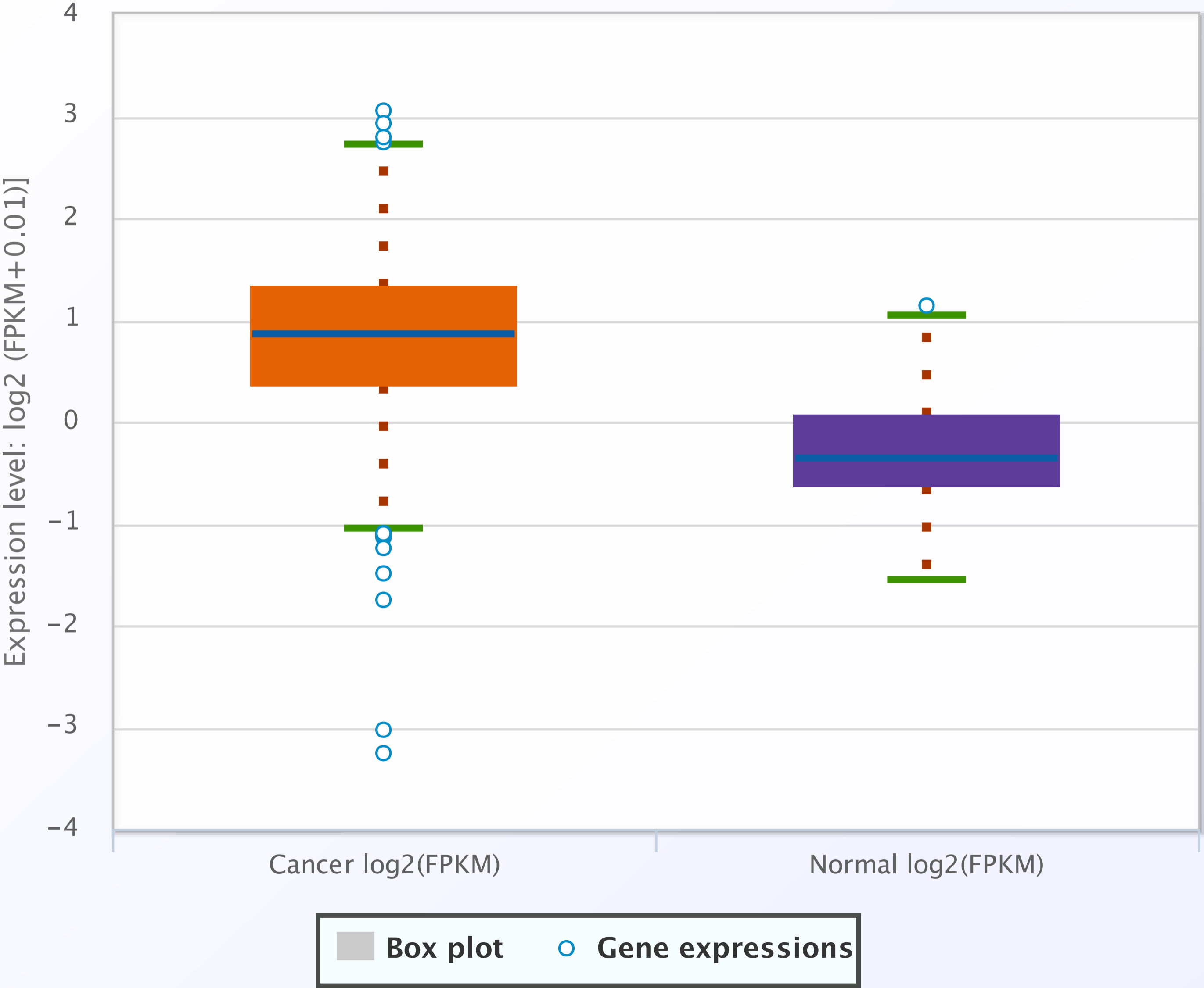

Supplement: Supplementary file 8 [file DataSheet1.ZIP › C1QTNF1 original data 1/LncRNA/lincRNA表达/ENCORI-AC040970.1_in_KIRC_boxplot(log2).pdf]

# AC239868.1 with 535 cancer and 72 normal samples in KIRC

Data Source: ENROCI project

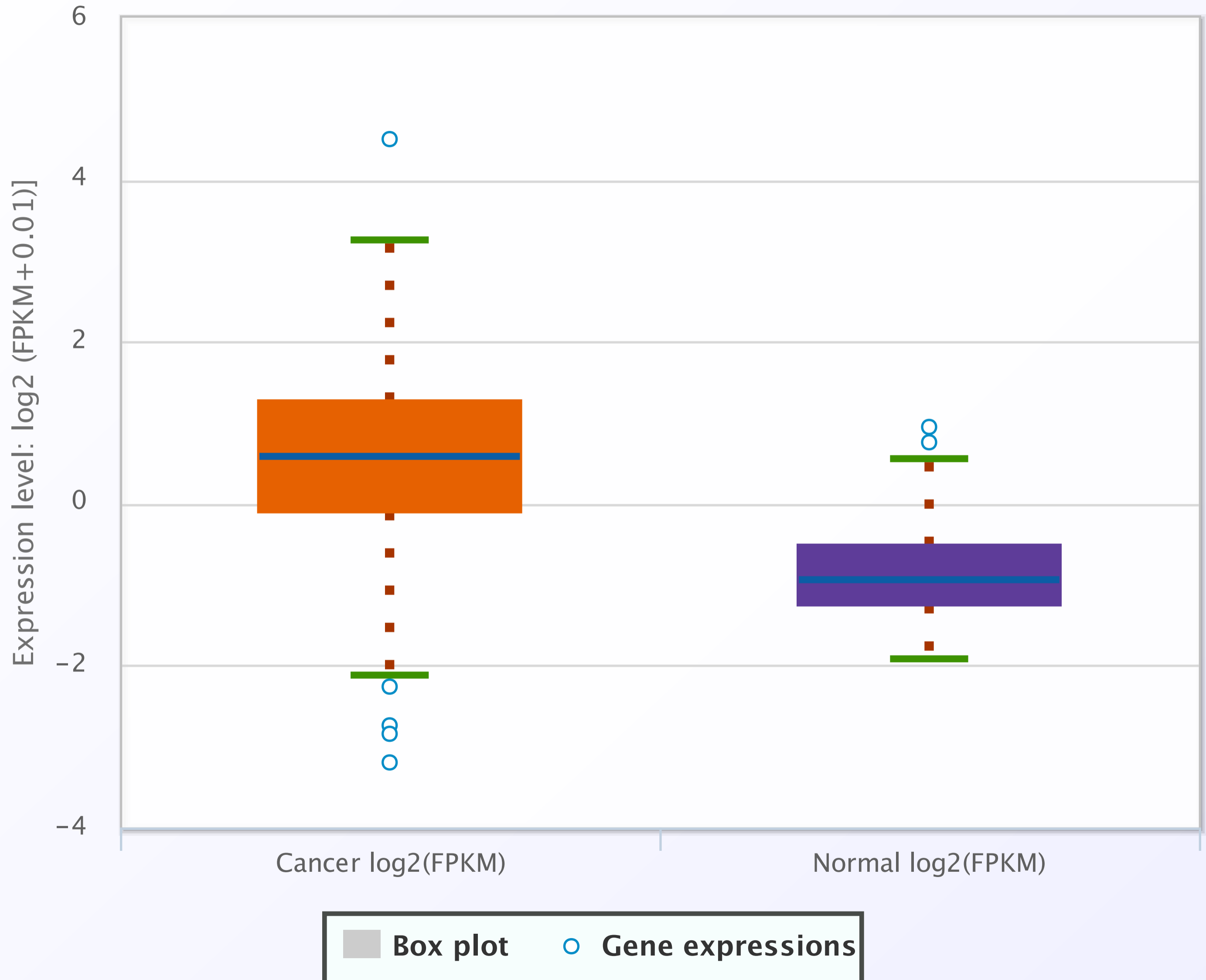

Supplement: Supplementary file 8 [file DataSheet1.ZIP › C1QTNF1 original data 1/LncRNA/lincRNA表达/ENCORI-AC239868.1_in_KIRC_boxplot(log2).pdf]

# CASC19 with 535 cancer and 72 normal samples in KIRC

Data Source: ENROCI project

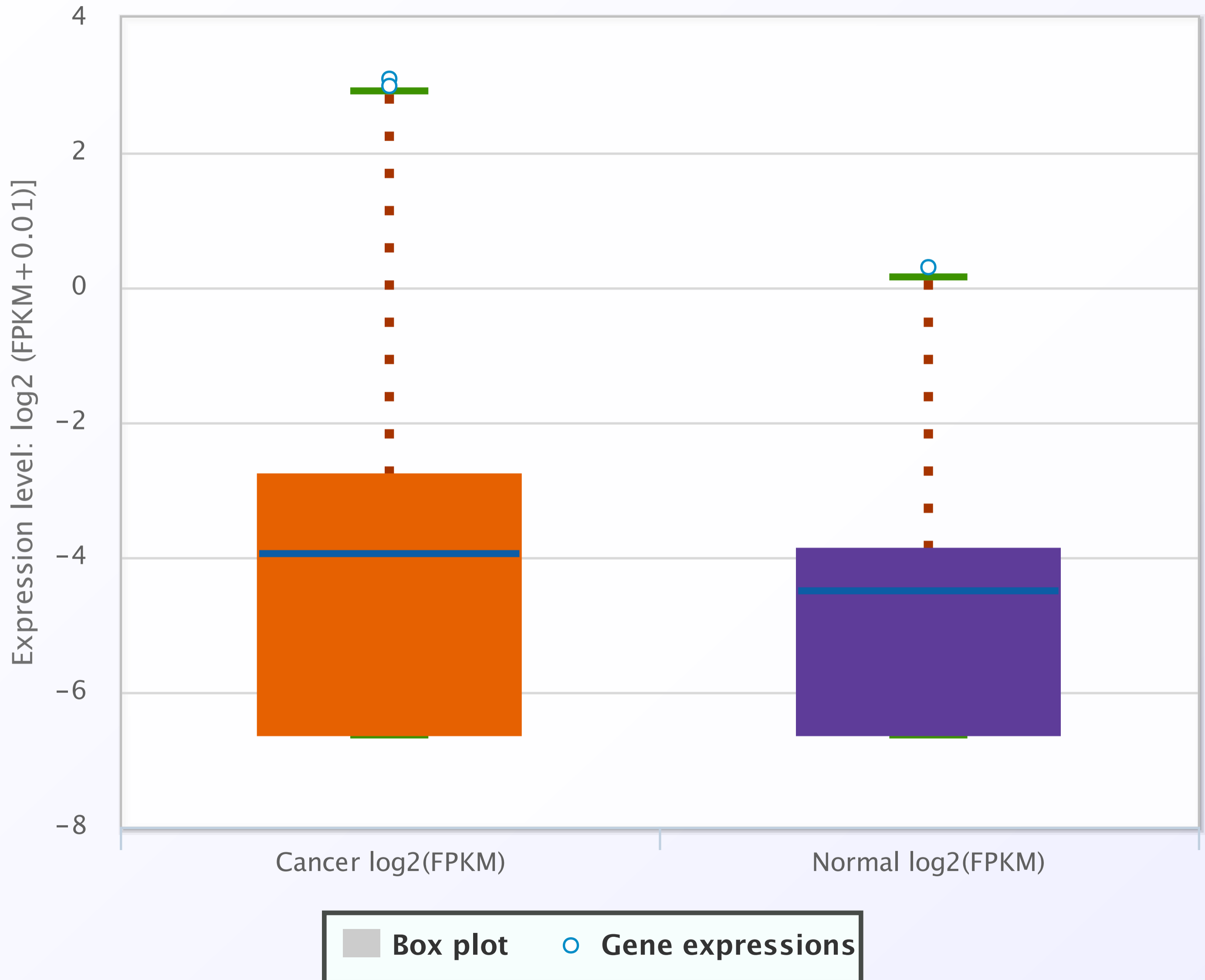

Supplement: Supplementary file 8 [file DataSheet1.ZIP › C1QTNF1 original data 1/LncRNA/lincRNA表达/ENCORI-CASC19_in_KIRC_boxplot(log2).pdf]

# CYTOR with 535 cancer and 72 normal samples in KIRC

Data Source: ENROCI project

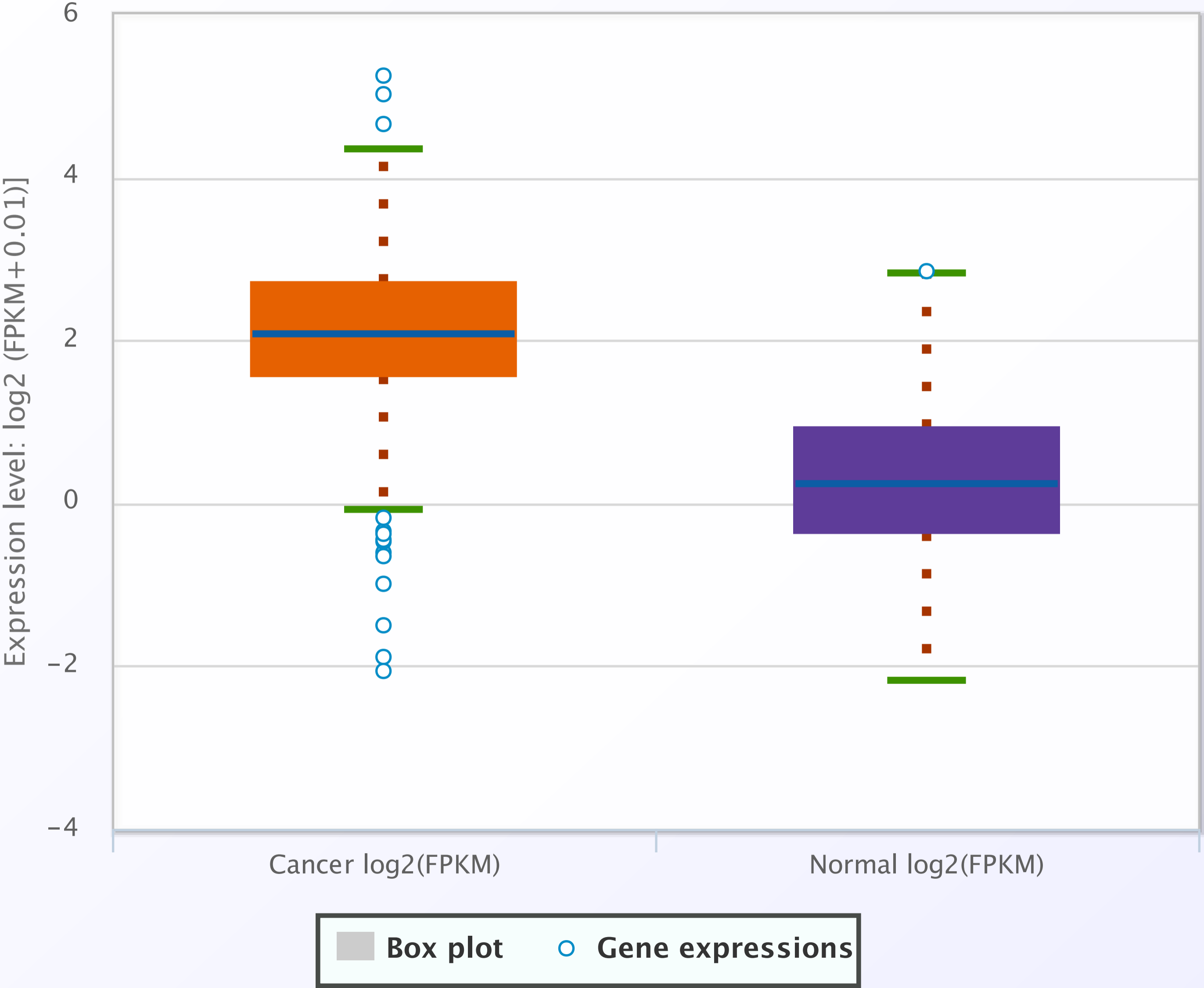

Supplement: Supplementary file 8 [file DataSheet1.ZIP › C1QTNF1 original data 1/LncRNA/lincRNA表达/ENCORI-CYTOR_in_KIRC_boxplot(log2).pdf]

# LINC02381 with 535 cancer and 72 normal samples in KIRC

Data Source: ENROCI project

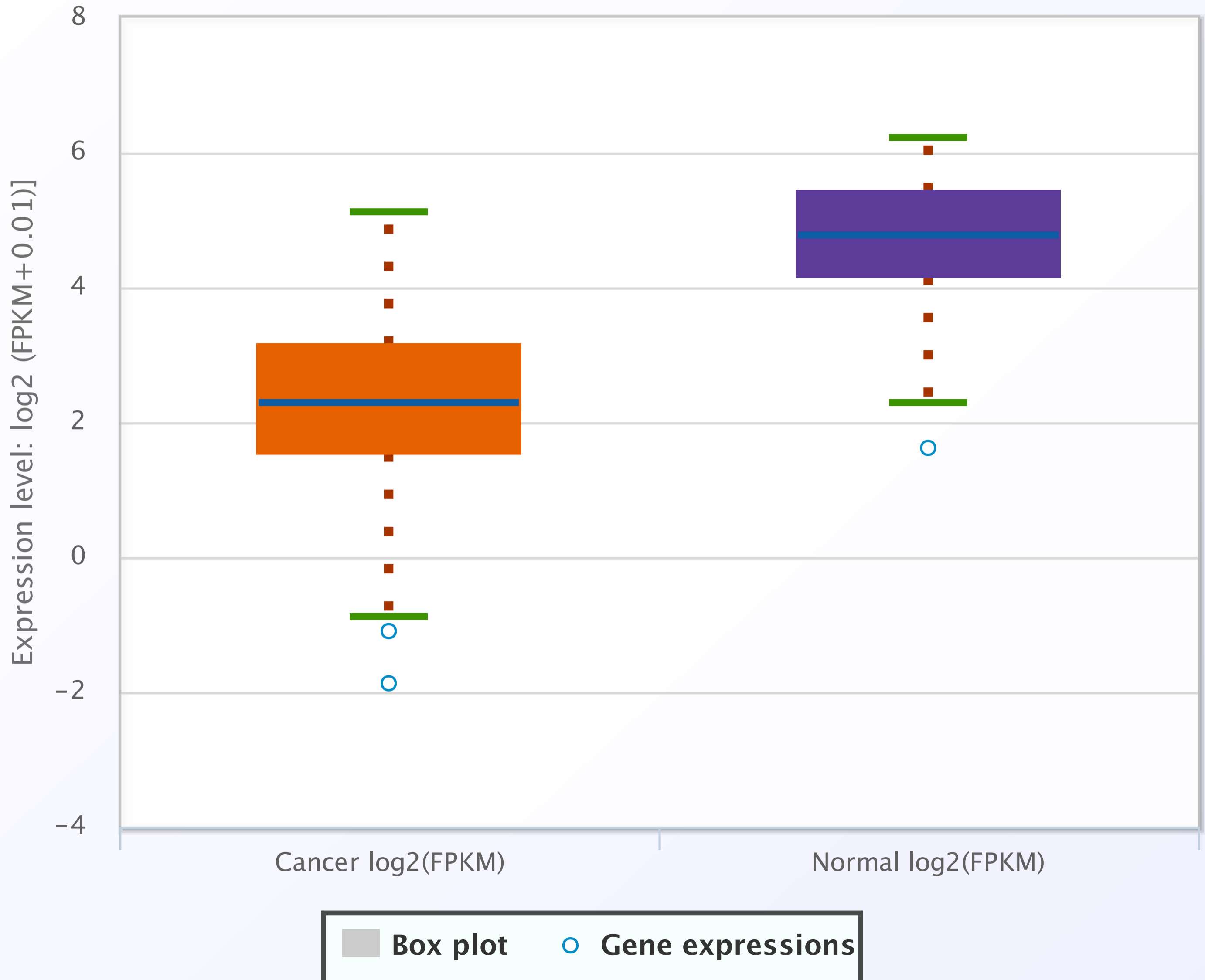

Supplement: Supplementary file 8 [file DataSheet1.ZIP › C1QTNF1 original data 1/LncRNA/lincRNA表达/ENCORI-LINC02381_in_KIRC_boxplot(log2).pdf]

# hsa-miR-27b-3p vs. C1QTNF1, 517 samples (KIRC)

Data Source: ENCORI project

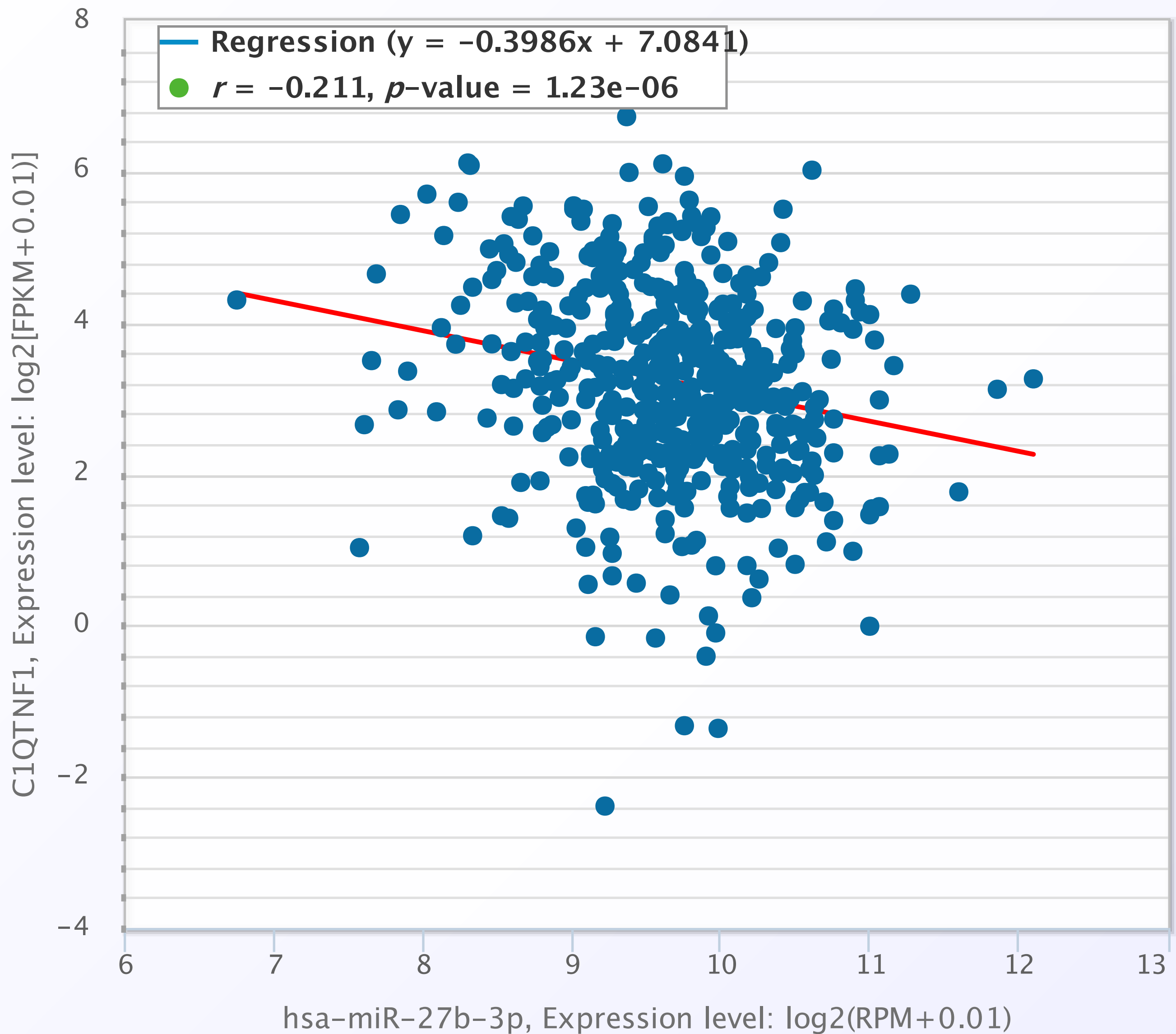

Supplement: Supplementary file 8 [file DataSheet1.ZIP › C1QTNF1 original data 1/miRNA/ENCORI-hsa-miR-27b-3p_and_C1QTNF1_coExp_in_miRNA_scatter_log2.pdf]

# hsa-miR-27b-3p with 517 cancer and 71 normal samples in KIRC

Data Source: ENROCI project

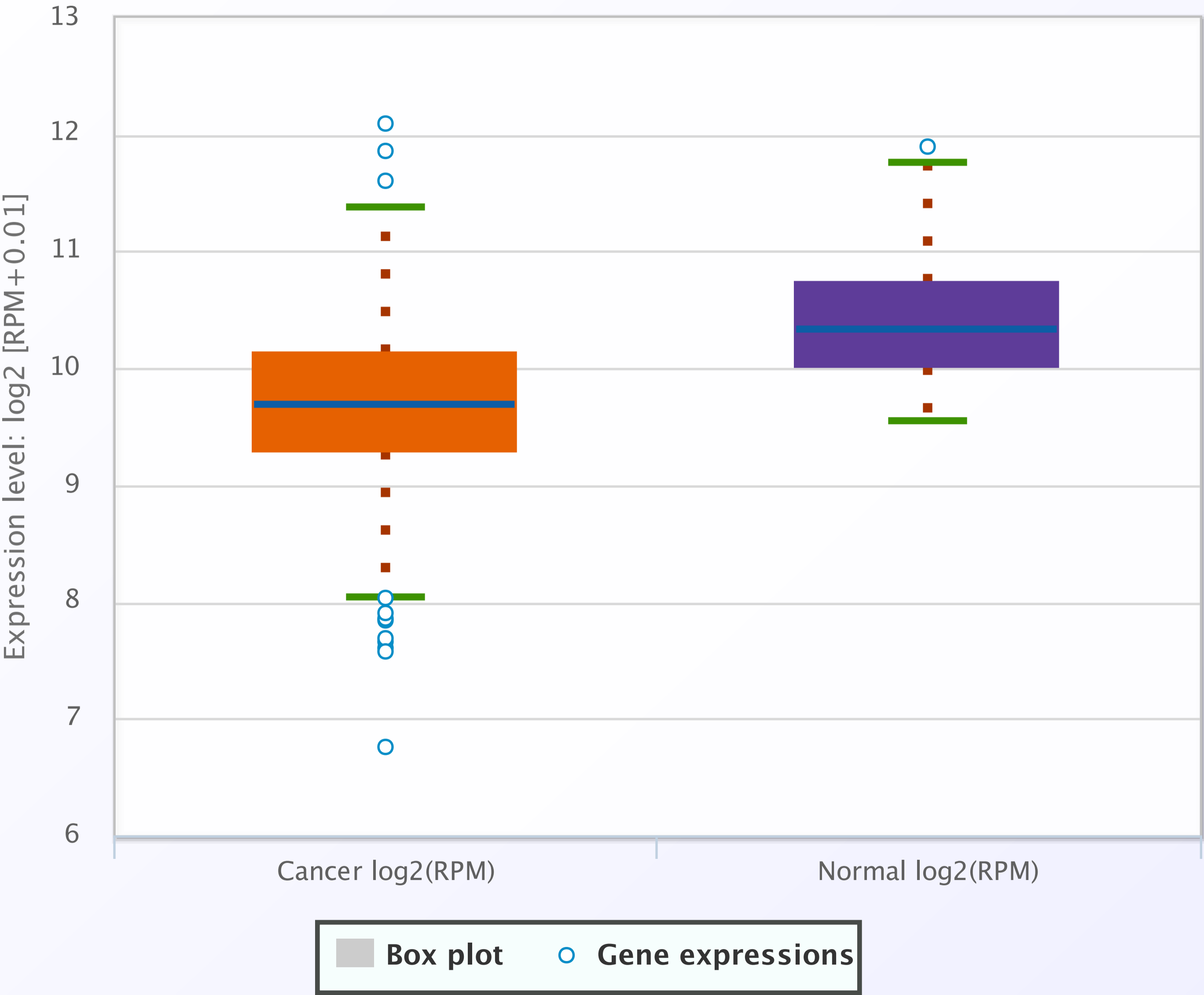

Supplement: Supplementary file 8 [file DataSheet1.ZIP › C1QTNF1 original data 1/miRNA/ENCORI-hsa-miR-27b-3p_in_KIRC_boxplot(log2).pdf]

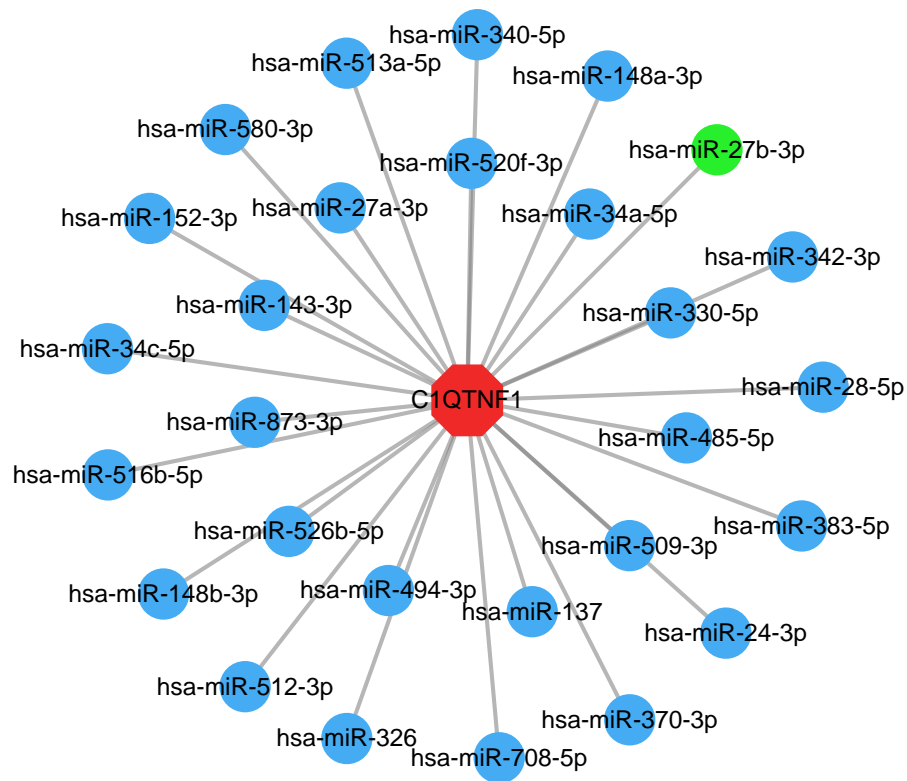

Supplement: Supplementary file 8 [file DataSheet1.ZIP › C1QTNF1 original data 1/miRNA/Interaction.pdf]

# hsa-mir-27b

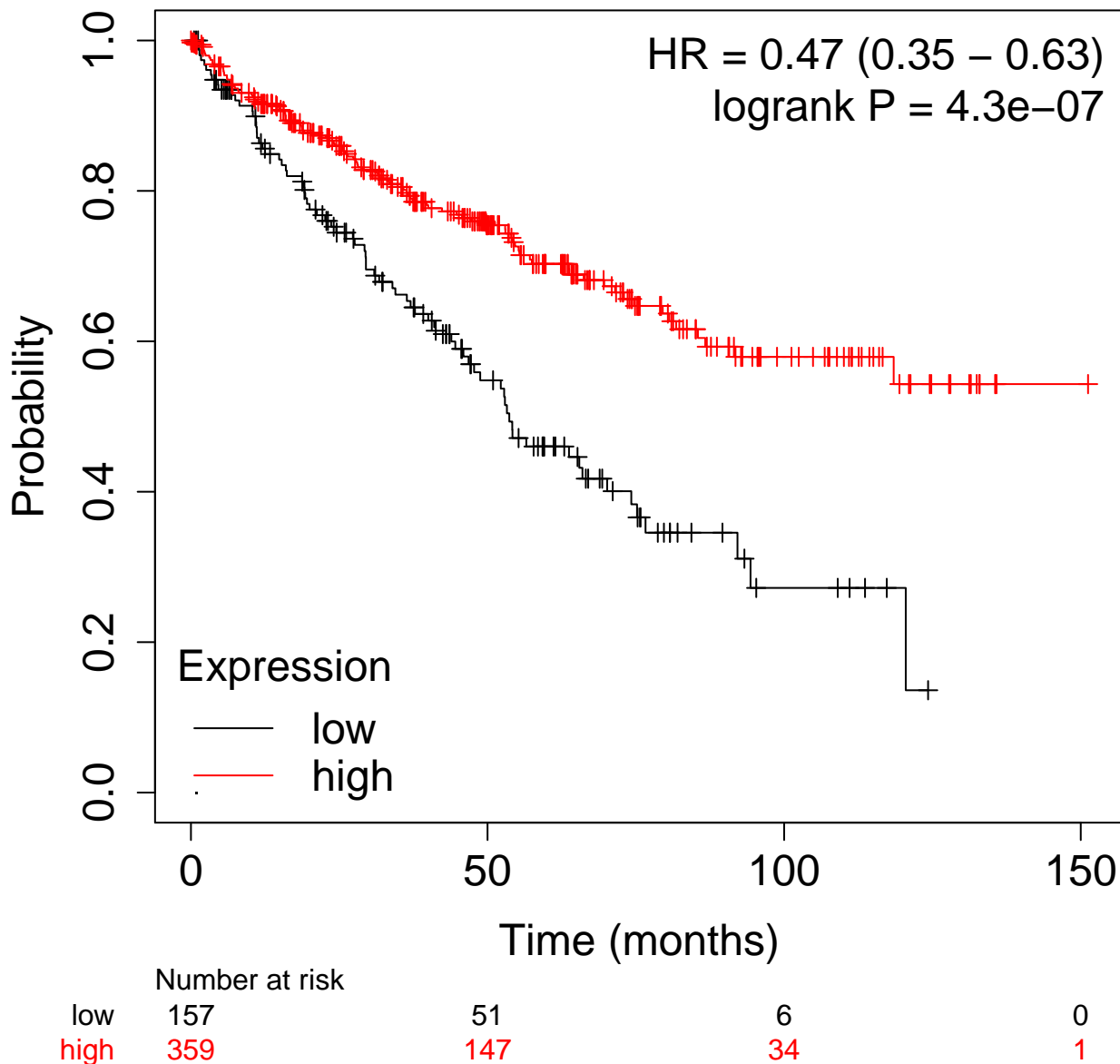

Supplement: Supplementary file 8 [file DataSheet1.ZIP › C1QTNF1 original data 1/miRNA/km_221114_071626_928900_6371eb4ae2c90_hsa-mir-27b.pdf]

The expression of hsa-miR-27b-3p  
 $\text{Log}_2(\text{RPM}+1)$

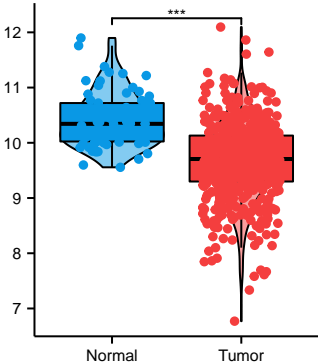

Supplement: Supplementary file 8 [file DataSheet1.ZIP › C1QTNF1 original data 1/miRNA/表达差异_非配对样本_2022-11-14_15_15_26.pdf]

The expression of C1QTNF1  
Log<sub>2</sub> (TPM+1)

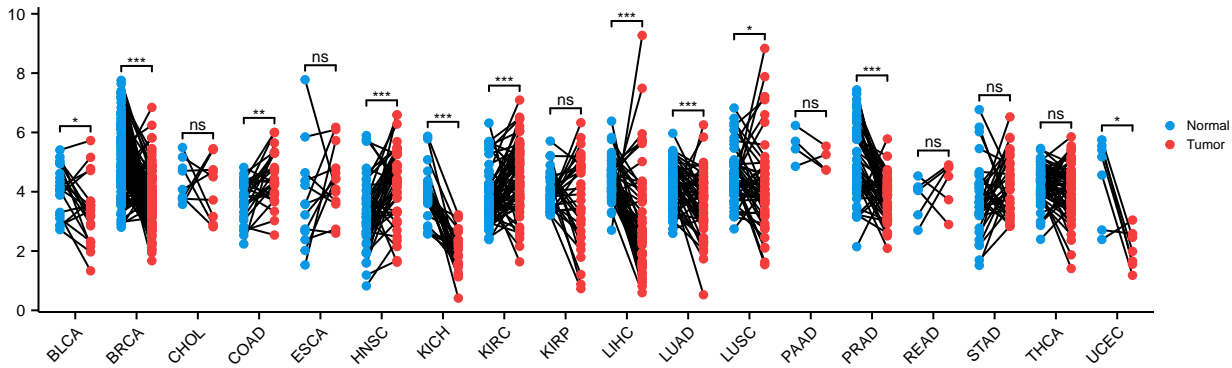

Supplement: Supplementary file 8 [file DataSheet1.ZIP › C1QTNF1 original data 1/Pan-cancer difference analysis/表达差异_配对样本_2022-11-11_16_10_26.pdf]

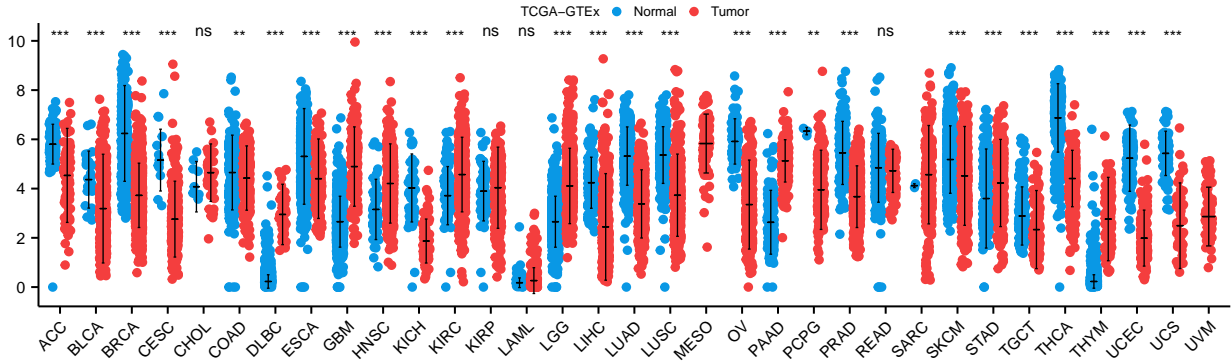

Supplement: Supplementary file 8 [file DataSheet1.ZIP › C1QTNF1 original data 1/Pan-cancer difference analysis/表达差异_非配对样本_2022-11-11_16_09_19.pdf]

# Overall Survival

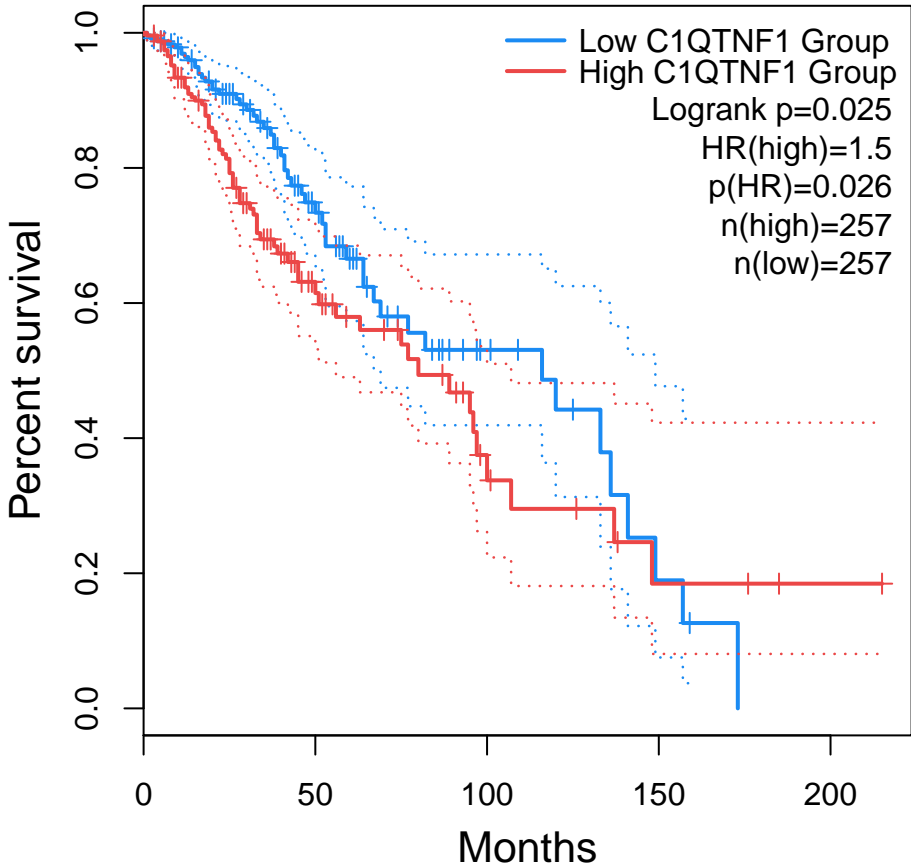

Supplement: Supplementary file 8 [file DataSheet1.ZIP › C1QTNF1 original data 1/Pan-cancer prognostic analysis/C1QTNF1_survival_bk35r.pdf]

# Overall Survival

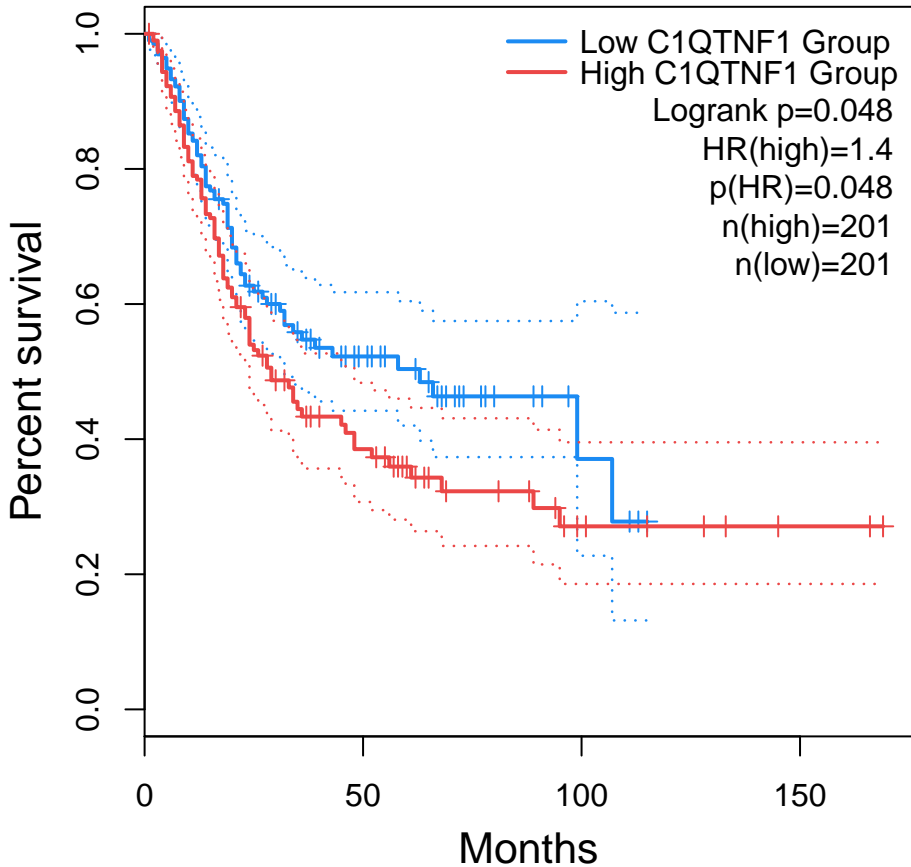

Supplement: Supplementary file 8 [file DataSheet1.ZIP › C1QTNF1 original data 1/Pan-cancer prognostic analysis/C1QTNF1_survival_ebudr.pdf]

# Overall Survival

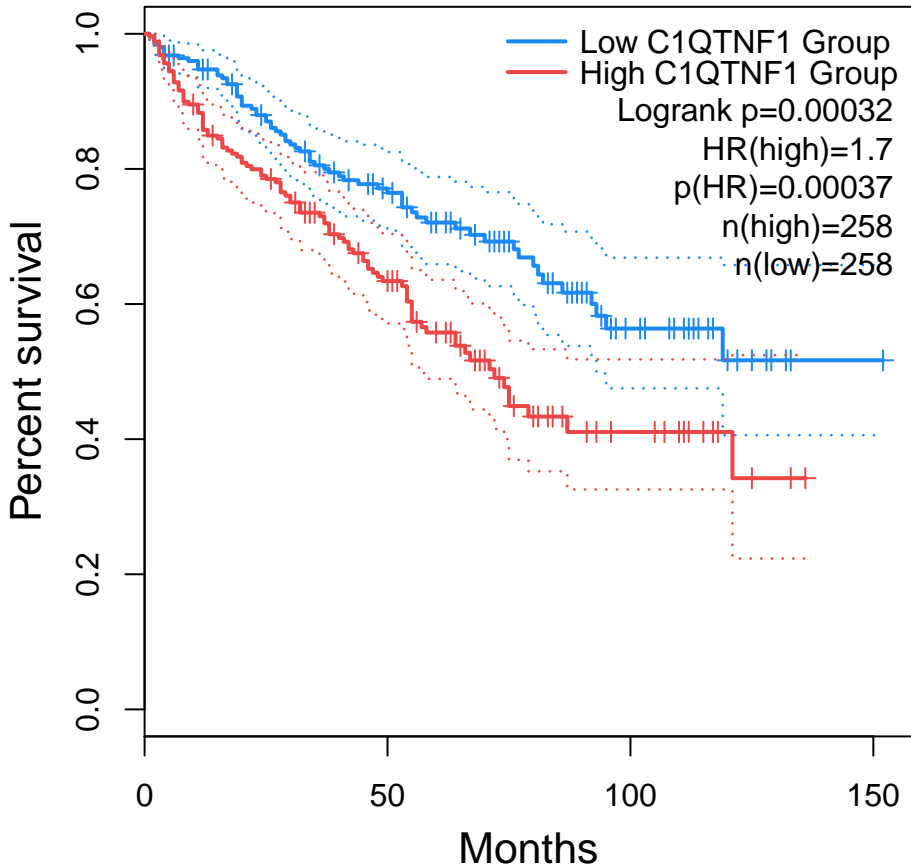

Supplement: Supplementary file 8 [file DataSheet1.ZIP › C1QTNF1 original data 1/Pan-cancer prognostic analysis/C1QTNF1_survival_fSPV5.pdf]

# Overall Survival

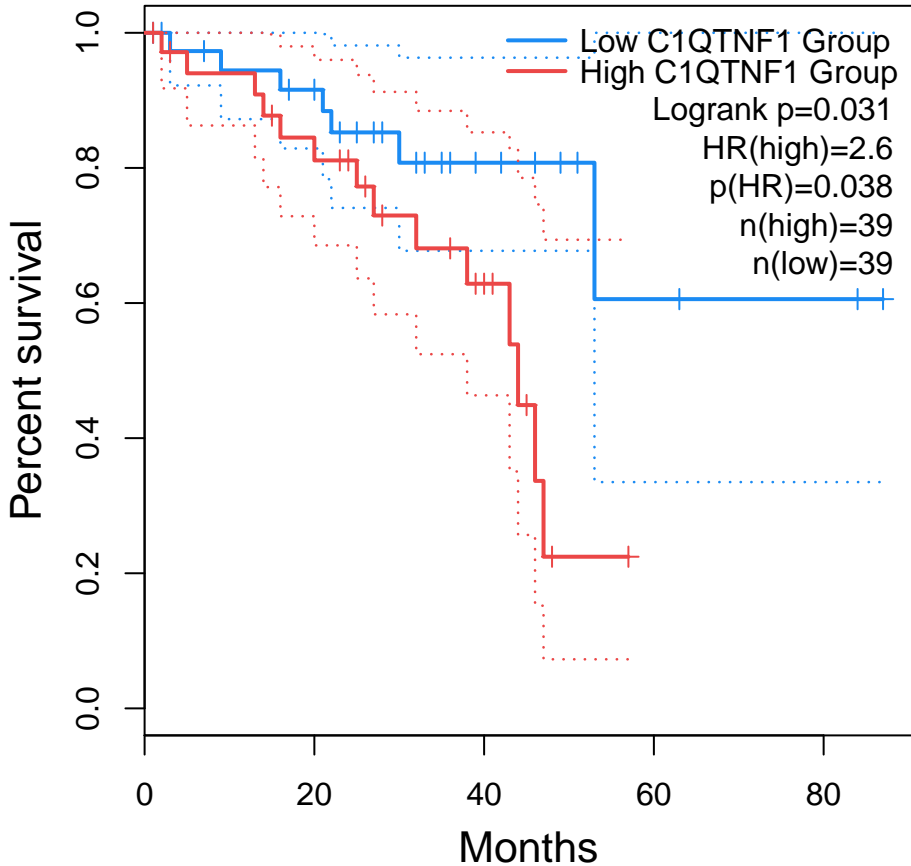

Supplement: Supplementary file 8 [file DataSheet1.ZIP › C1QTNF1 original data 1/Pan-cancer prognostic analysis/C1QTNF1_survival_Okf0J.pdf]

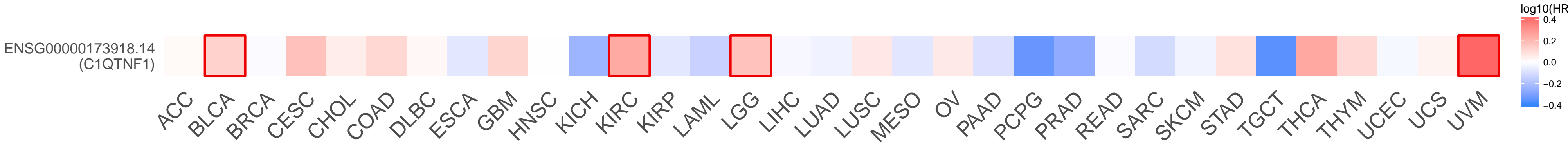

Supplement: Supplementary file 8 [file DataSheet1.ZIP › C1QTNF1 original data 1/Pan-cancer prognostic analysis/survival_map_g7elh.pdf]

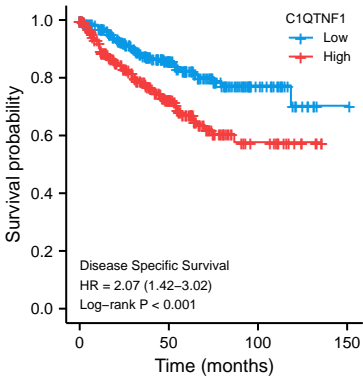

Supplement: Supplementary file 8 [file DataSheet1.ZIP › C1QTNF1 original data 1/Prognostic analysis of KIRC/预后分析_DSS.pdf]

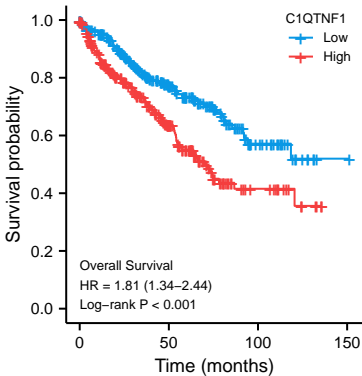

Supplement: Supplementary file 8 [file DataSheet1.ZIP › C1QTNF1 original data 1/Prognostic analysis of KIRC/预后分析_OS.pdf]

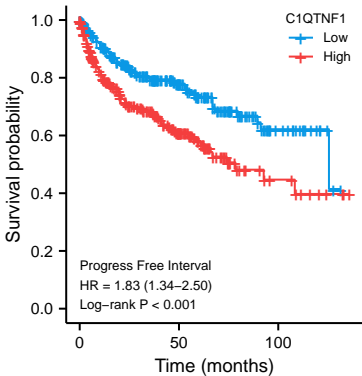

Supplement: Supplementary file 8 [file DataSheet1.ZIP › C1QTNF1 original data 1/Prognostic analysis of KIRC/预后分析_PFI.pdf]

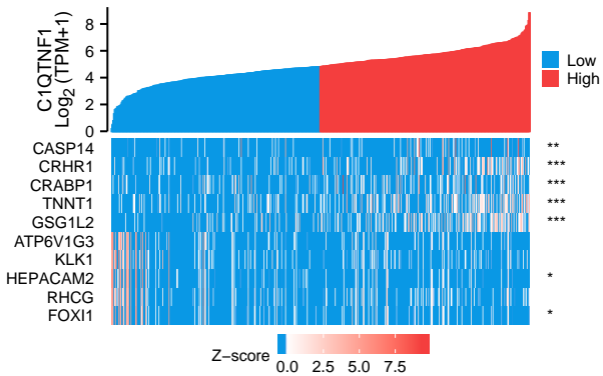

Supplement: Supplementary file 8 [file DataSheet1.ZIP › C1QTNF1 original data 1/Single gene difference analysis/单基因共表达热图_2022-11-12_00_35_26.pdf]

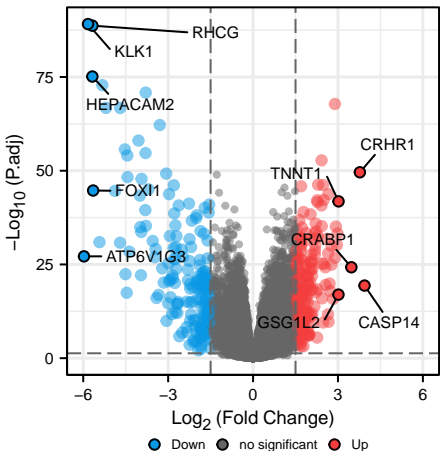

Supplement: Supplementary file 8 [file DataSheet1.ZIP › C1QTNF1 original data 1/Single gene difference analysis/火山图_2022-11-16_11_31_52.pdf]

# M stage: M0

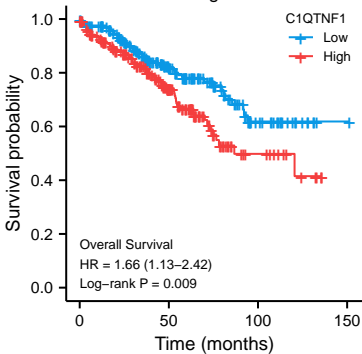

Supplement: Supplementary file 8 [file DataSheet1.ZIP › C1QTNF1 original data 1/Subgroup survival prognosis analysis/M0.pdf]

# M stage: M1

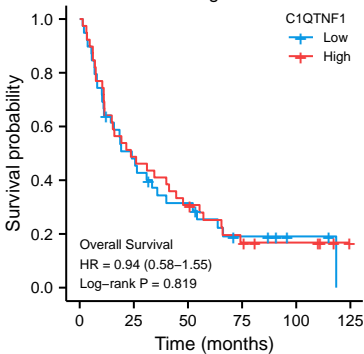

Supplement: Supplementary file 8 [file DataSheet1.ZIP › C1QTNF1 original data 1/Subgroup survival prognosis analysis/M1.pdf]

# N stage: N0

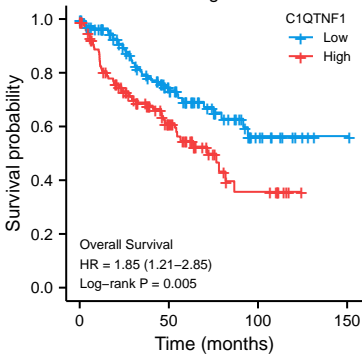

Supplement: Supplementary file 8 [file DataSheet1.ZIP › C1QTNF1 original data 1/Subgroup survival prognosis analysis/N0.pdf]

# N stage: N1

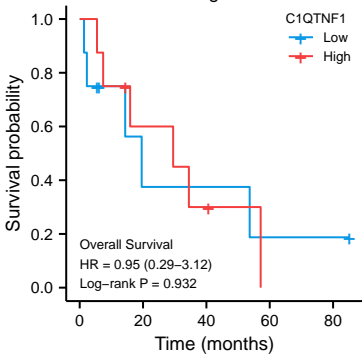

Supplement: Supplementary file 8 [file DataSheet1.ZIP › C1QTNF1 original data 1/Subgroup survival prognosis analysis/N1.pdf]

# Pathologic stage: Stage I&Stage II

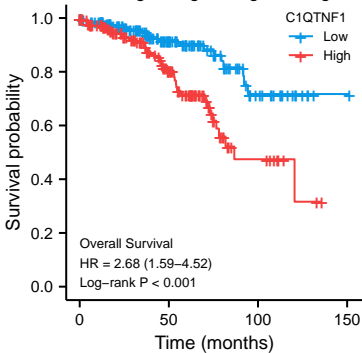

Supplement: Supplementary file 8 [file DataSheet1.ZIP › C1QTNF1 original data 1/Subgroup survival prognosis analysis/S12.pdf]

# Pathologic stage: Stage III&Stage IV

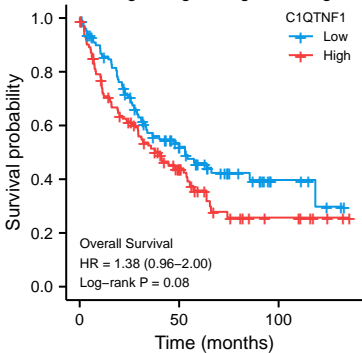

Supplement: Supplementary file 8 [file DataSheet1.ZIP › C1QTNF1 original data 1/Subgroup survival prognosis analysis/S34.pdf]

# T stage: T3&T4

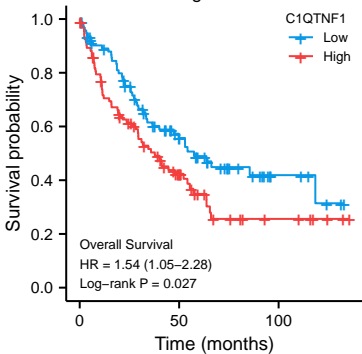

Supplement: Supplementary file 8 [file DataSheet1.ZIP › C1QTNF1 original data 1/Subgroup survival prognosis analysis/T34.pdf]

# T stage: T1&T2

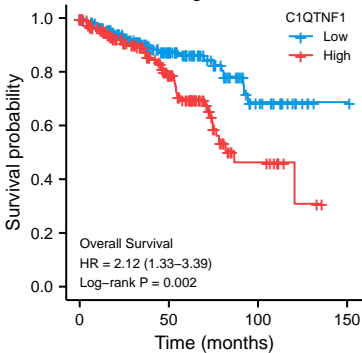

Supplement: Supplementary file 8 [file DataSheet1.ZIP › C1QTNF1 original data 1/Subgroup survival prognosis analysis/亚组KM图T12.pdf]

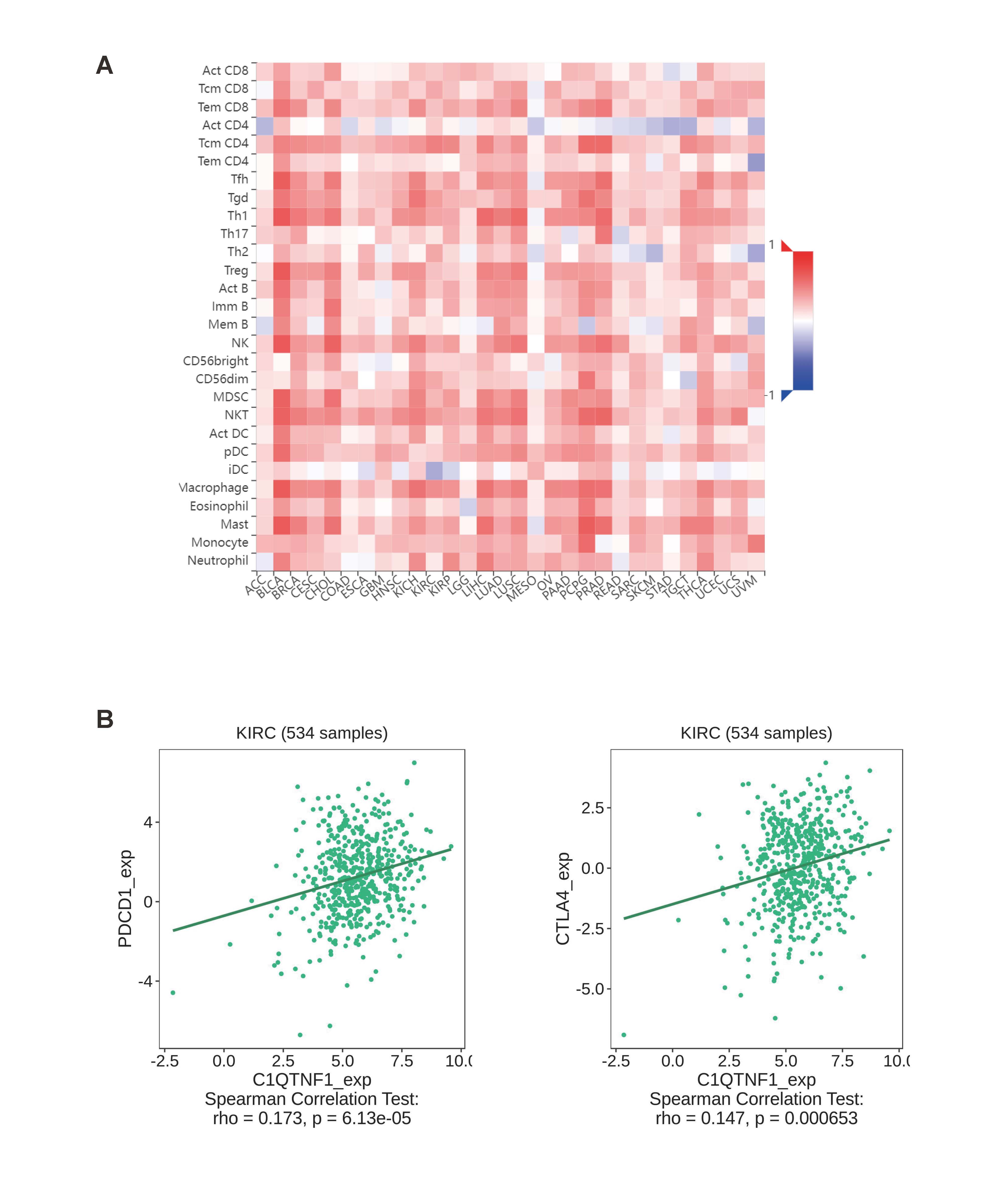

Supplement: Supplementary file 10 [file Image2.JPEG]

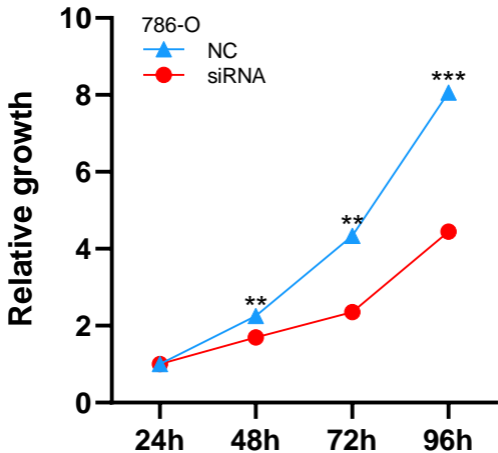

Supplement: Supplementary file 14 [file DataSheet2.ZIP › C1QTNF1 original data 2/CCK8/C1Q-786-C1Q敲除.pdf]

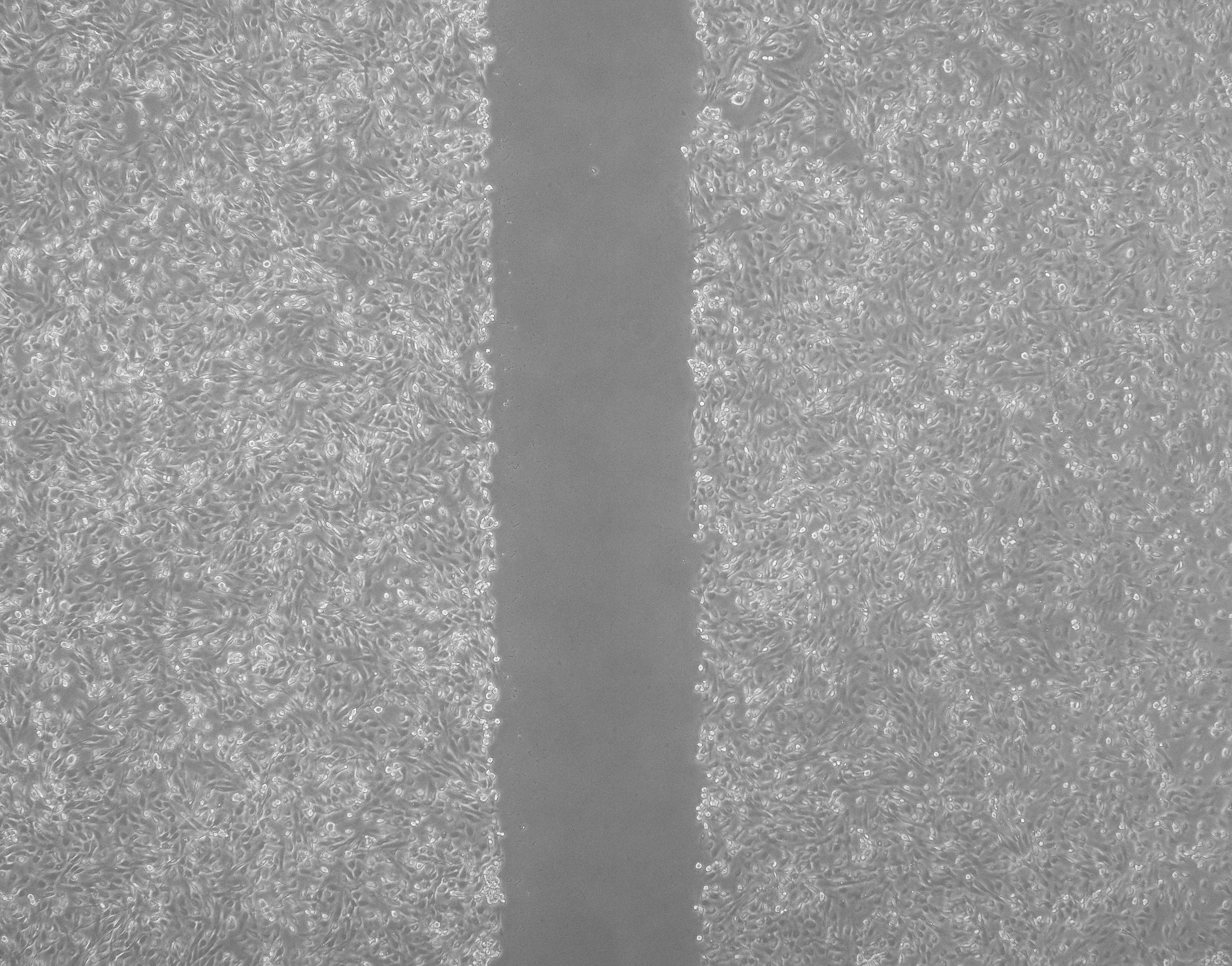

Supplement: Supplementary file 14 [file DataSheet2.ZIP › C1QTNF1 original data 2/Scratch test/nc-0.tif]

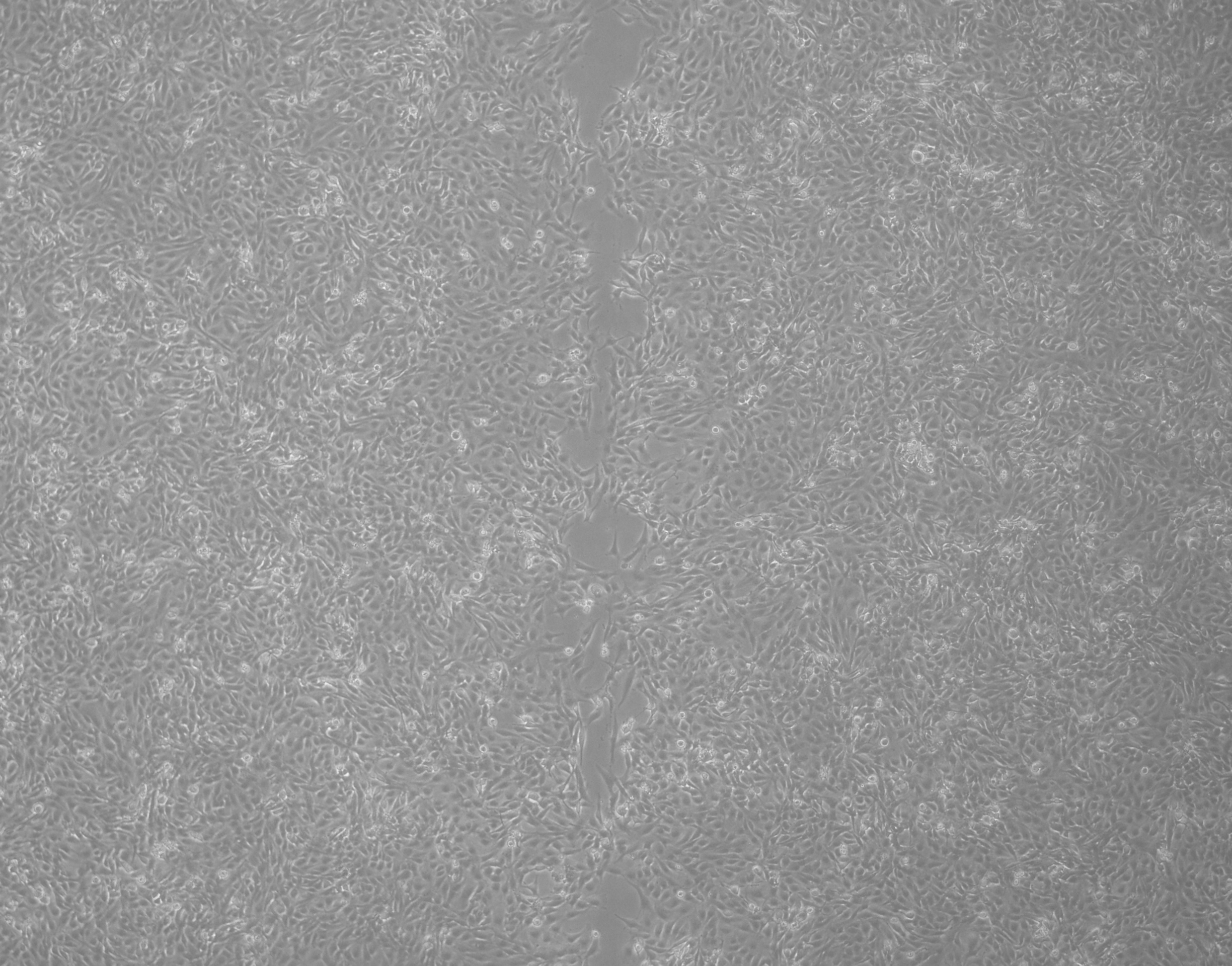

Supplement: Supplementary file 14 [file DataSheet2.ZIP › C1QTNF1 original data 2/Scratch test/nc-24.tif]

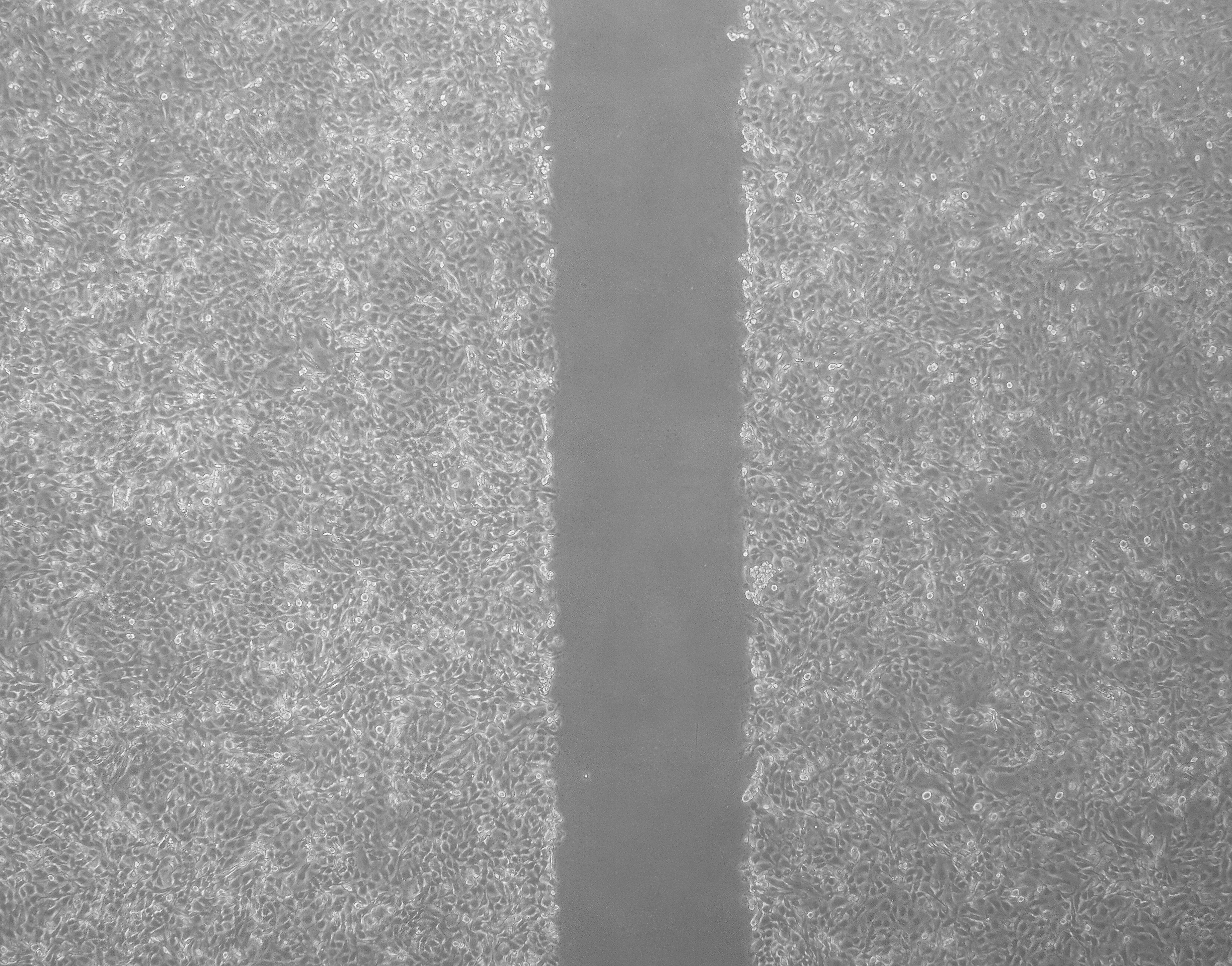

Supplement: Supplementary file 14 [file DataSheet2.ZIP › C1QTNF1 original data 2/Scratch test/si-0.tif]

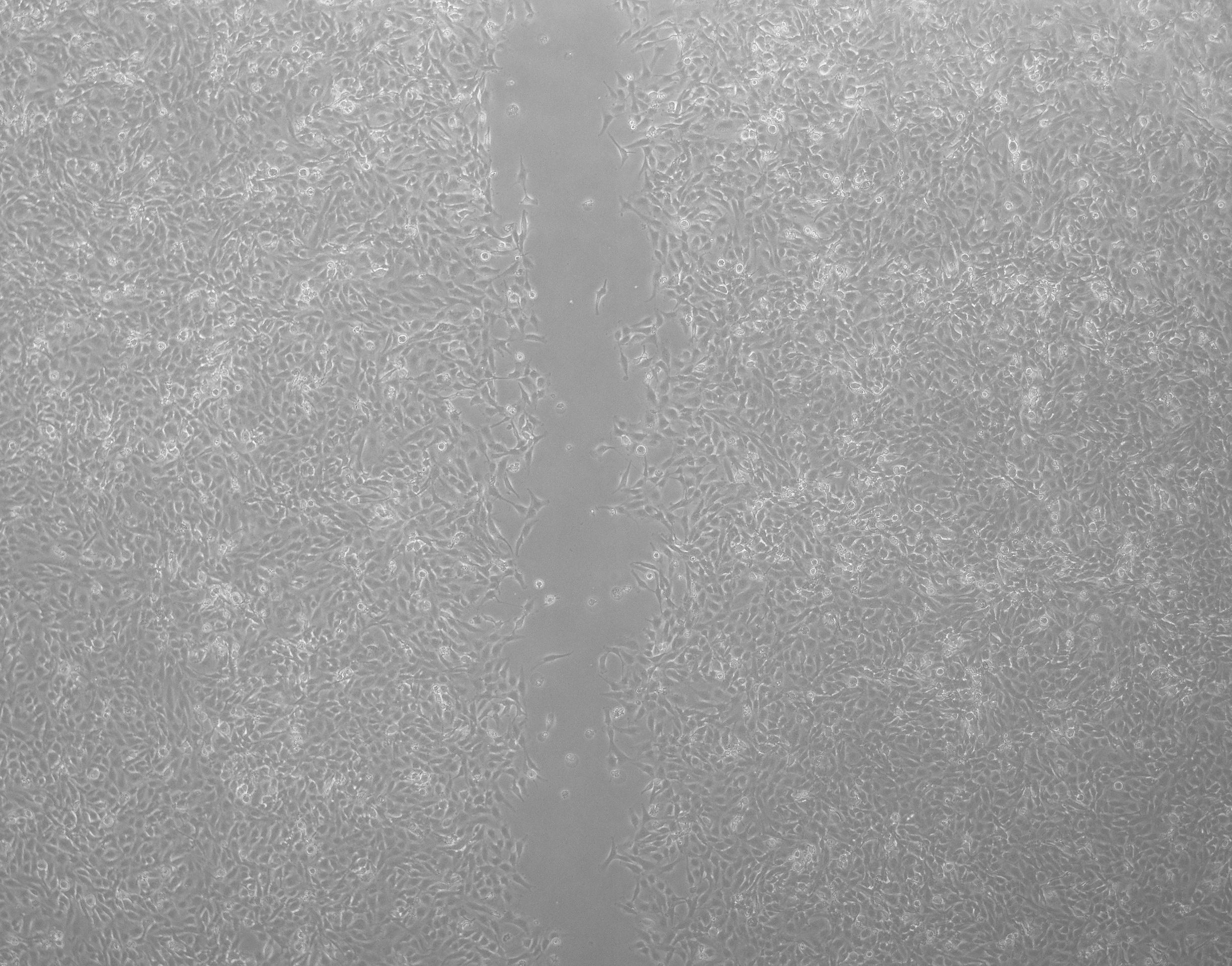

Supplement: Supplementary file 14 [file DataSheet2.ZIP › C1QTNF1 original data 2/Scratch test/si-24.tif]

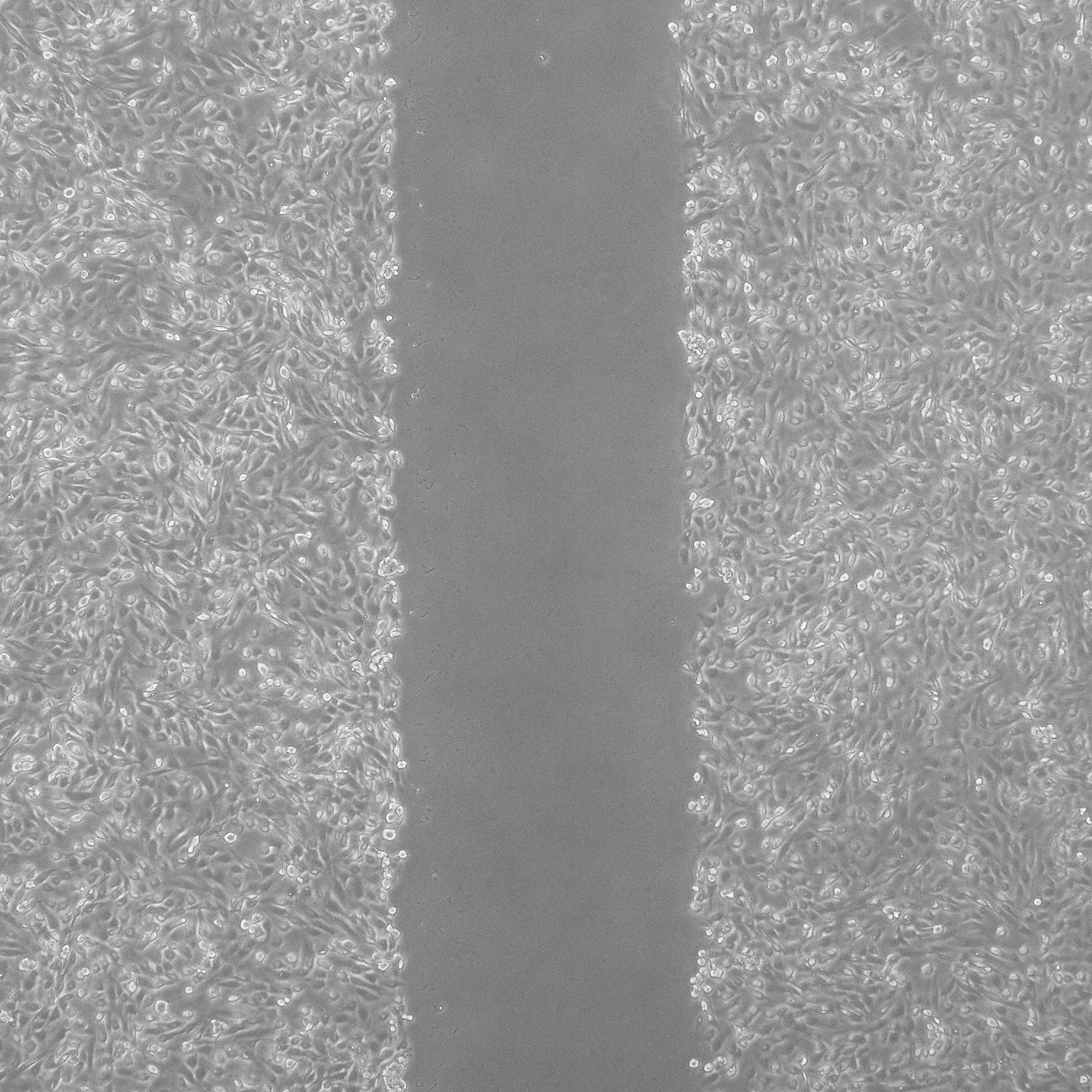

Supplement: Supplementary file 14 [file DataSheet2.ZIP › C1QTNF1 original data 2/Scratch test/图片处理/nc-0.tif]

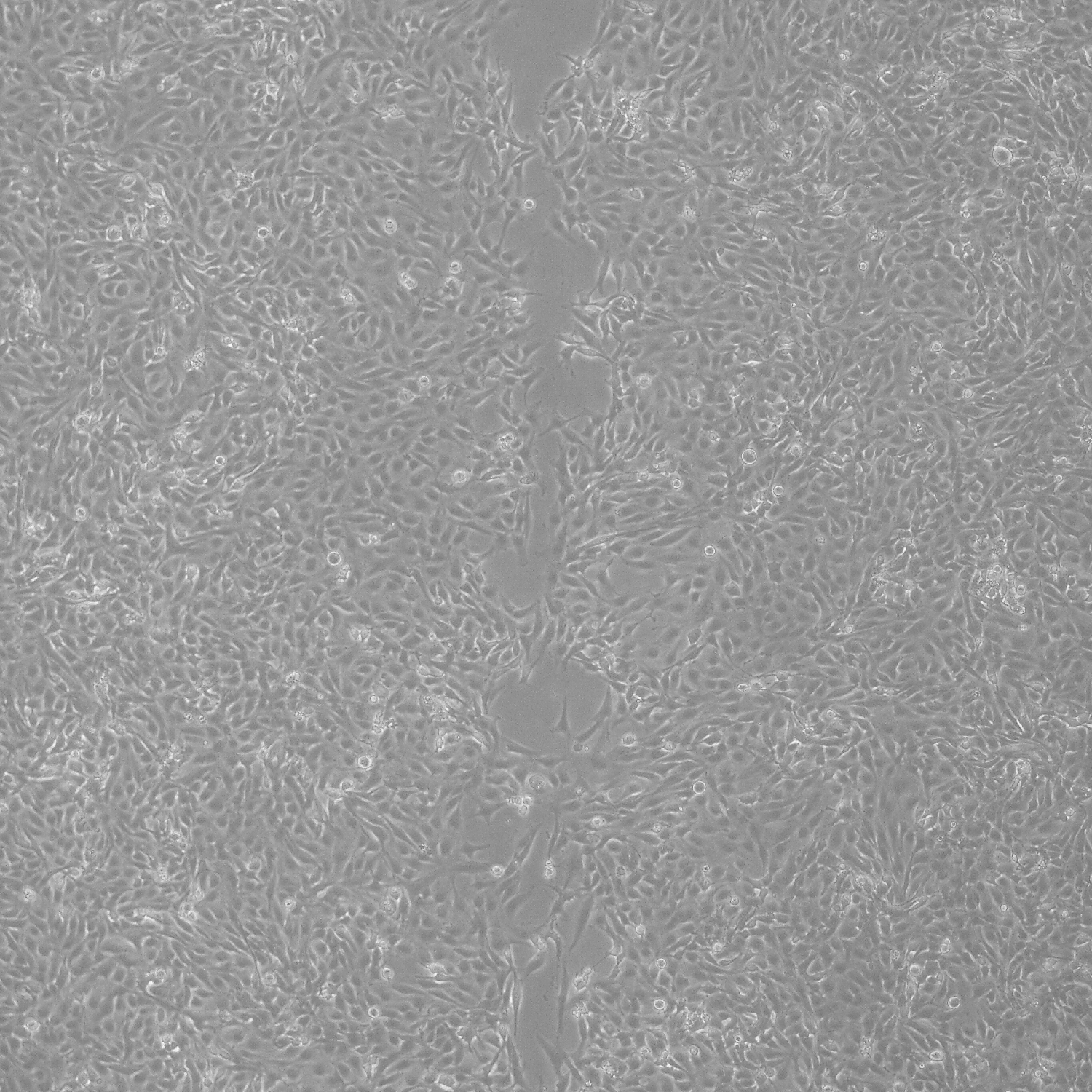

Supplement: Supplementary file 14 [file DataSheet2.ZIP › C1QTNF1 original data 2/Scratch test/图片处理/nc-24.tif]

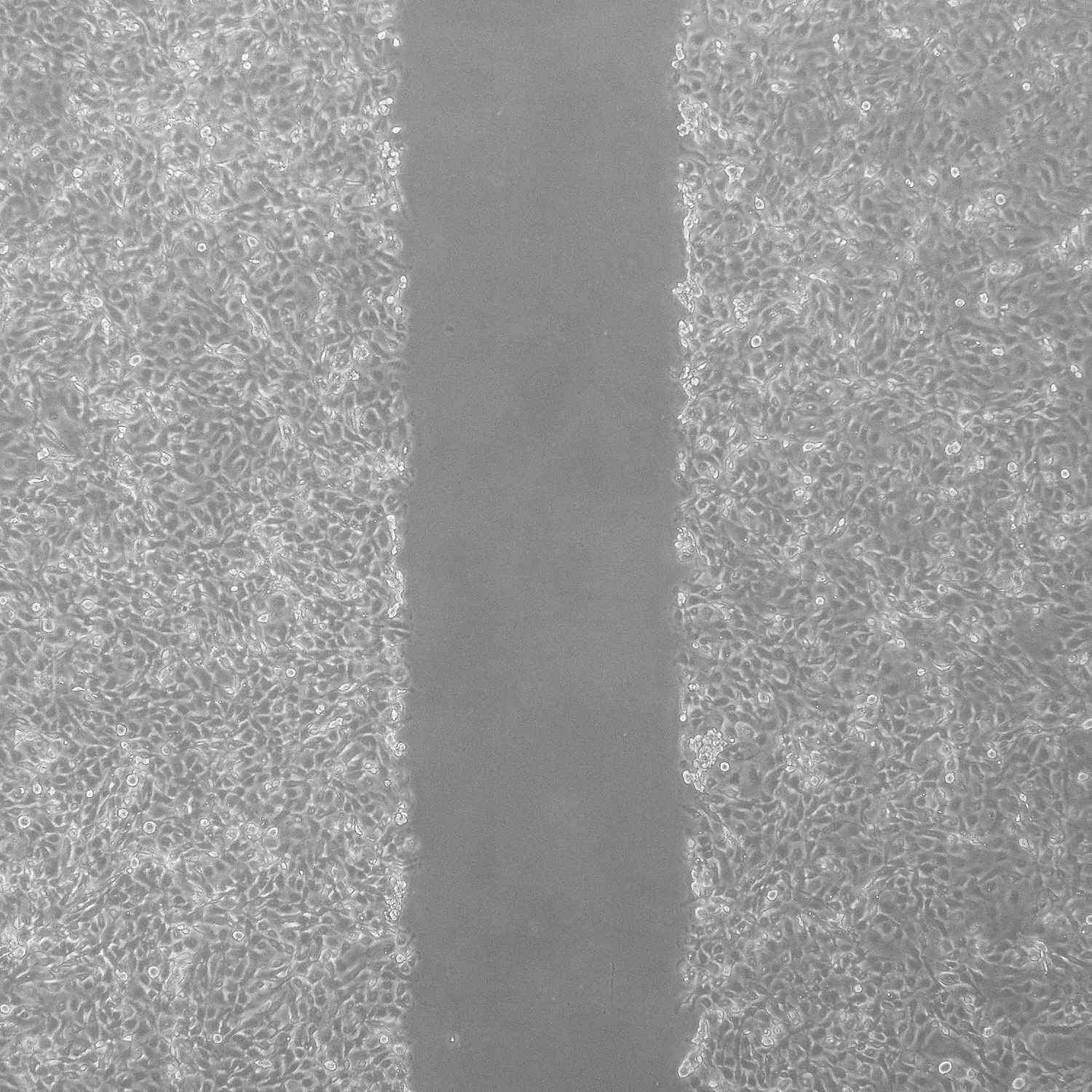

Supplement: Supplementary file 14 [file DataSheet2.ZIP › C1QTNF1 original data 2/Scratch test/图片处理/si-0.tif]

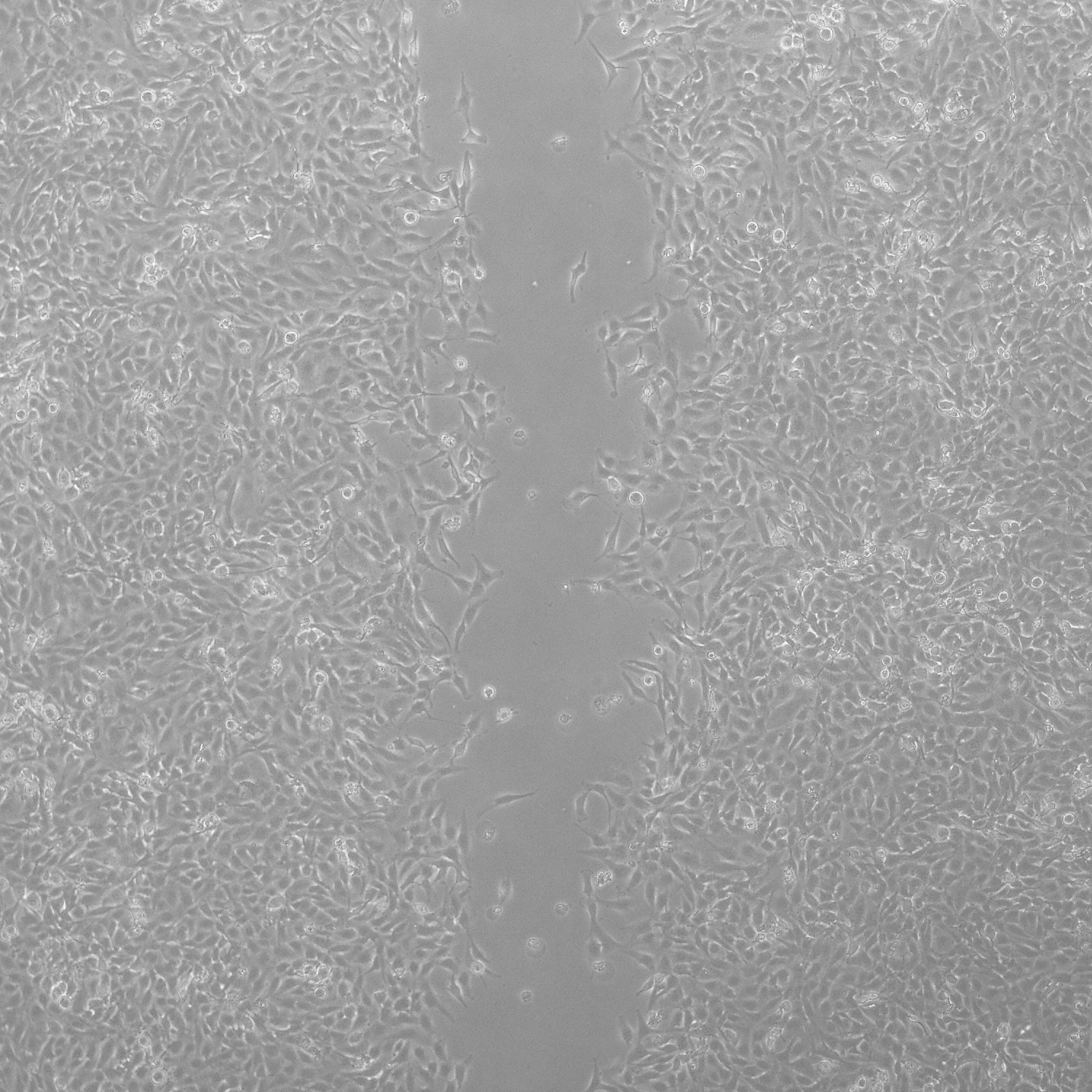

Supplement: Supplementary file 14 [file DataSheet2.ZIP › C1QTNF1 original data 2/Scratch test/图片处理/si-24.tif]

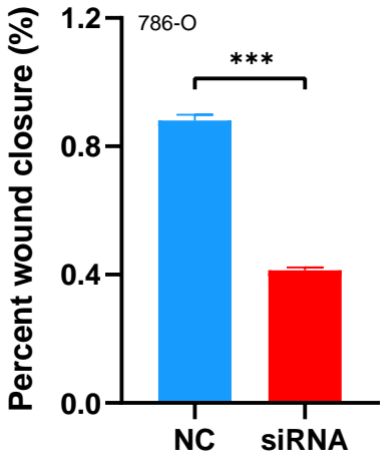

Supplement: Supplementary file 14 [file DataSheet2.ZIP › C1QTNF1 original data 2/Scratch test/图片处理/划痕半定量.pdf]

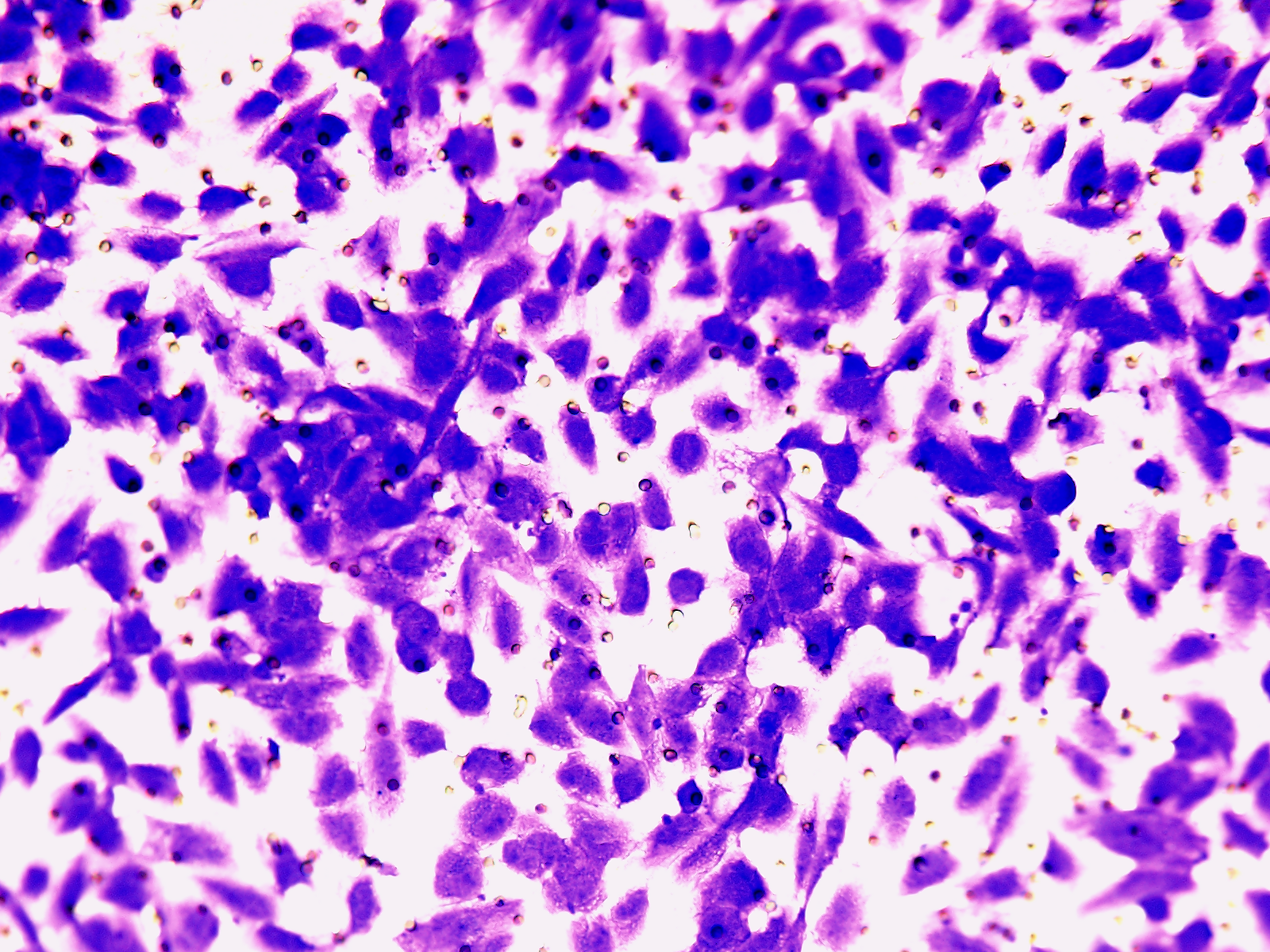

Supplement: Supplementary file 14 [file DataSheet2.ZIP › C1QTNF1 original data 2/transwell/in-nc.tif]

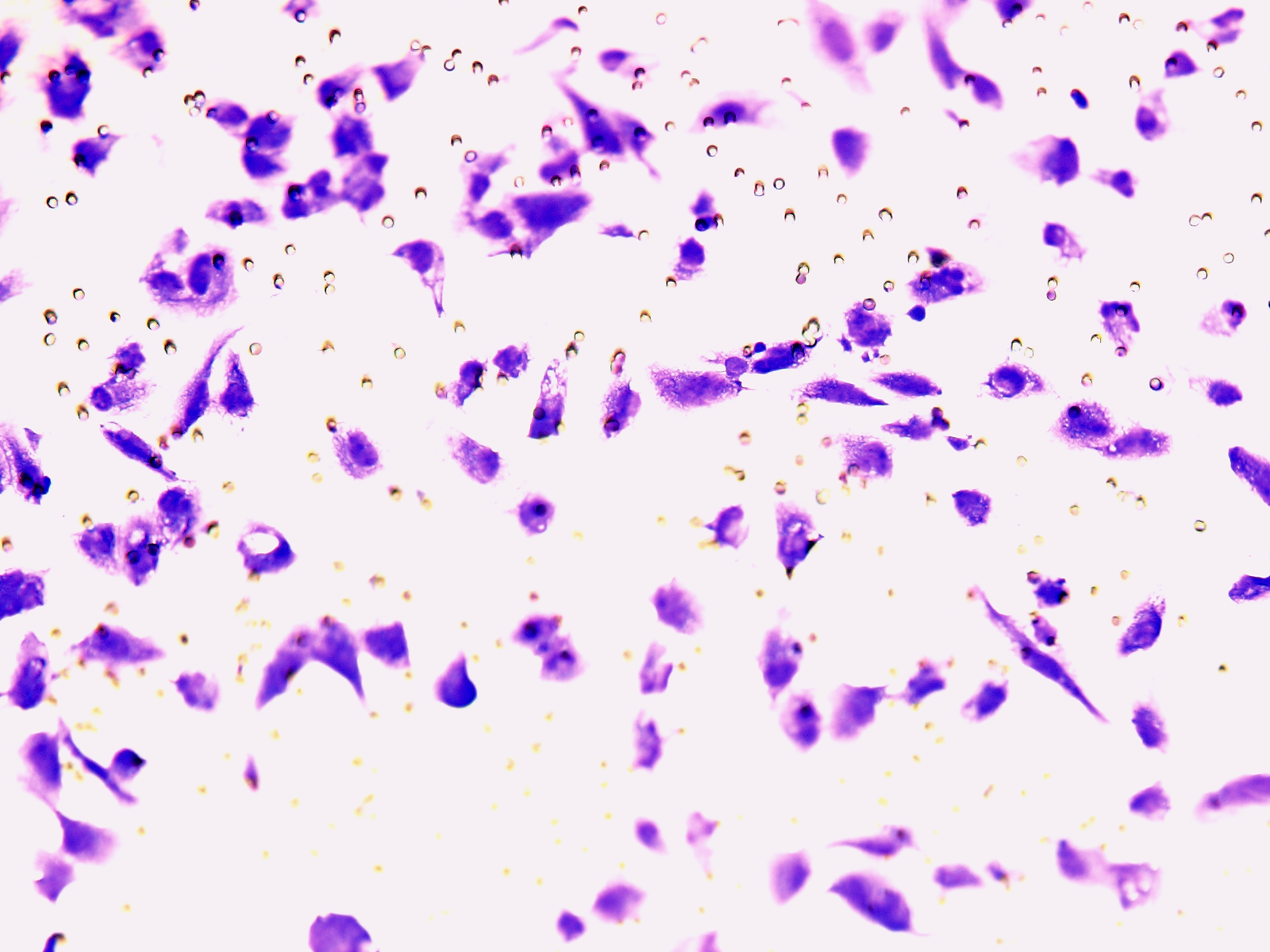

Supplement: Supplementary file 14 [file DataSheet2.ZIP › C1QTNF1 original data 2/transwell/in-si.tif]

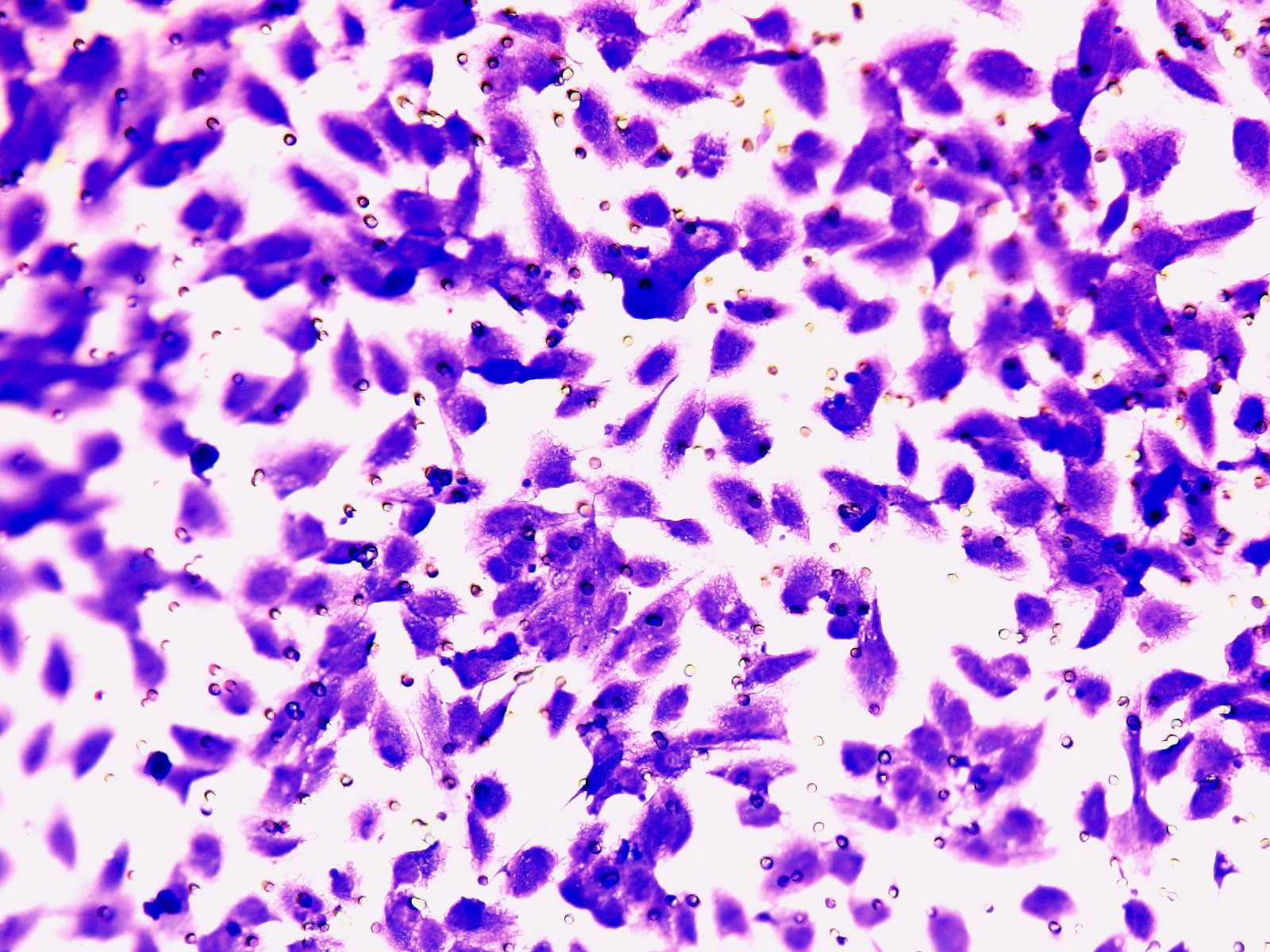

Supplement: Supplementary file 14 [file DataSheet2.ZIP › C1QTNF1 original data 2/transwell/mi-nc.tif]

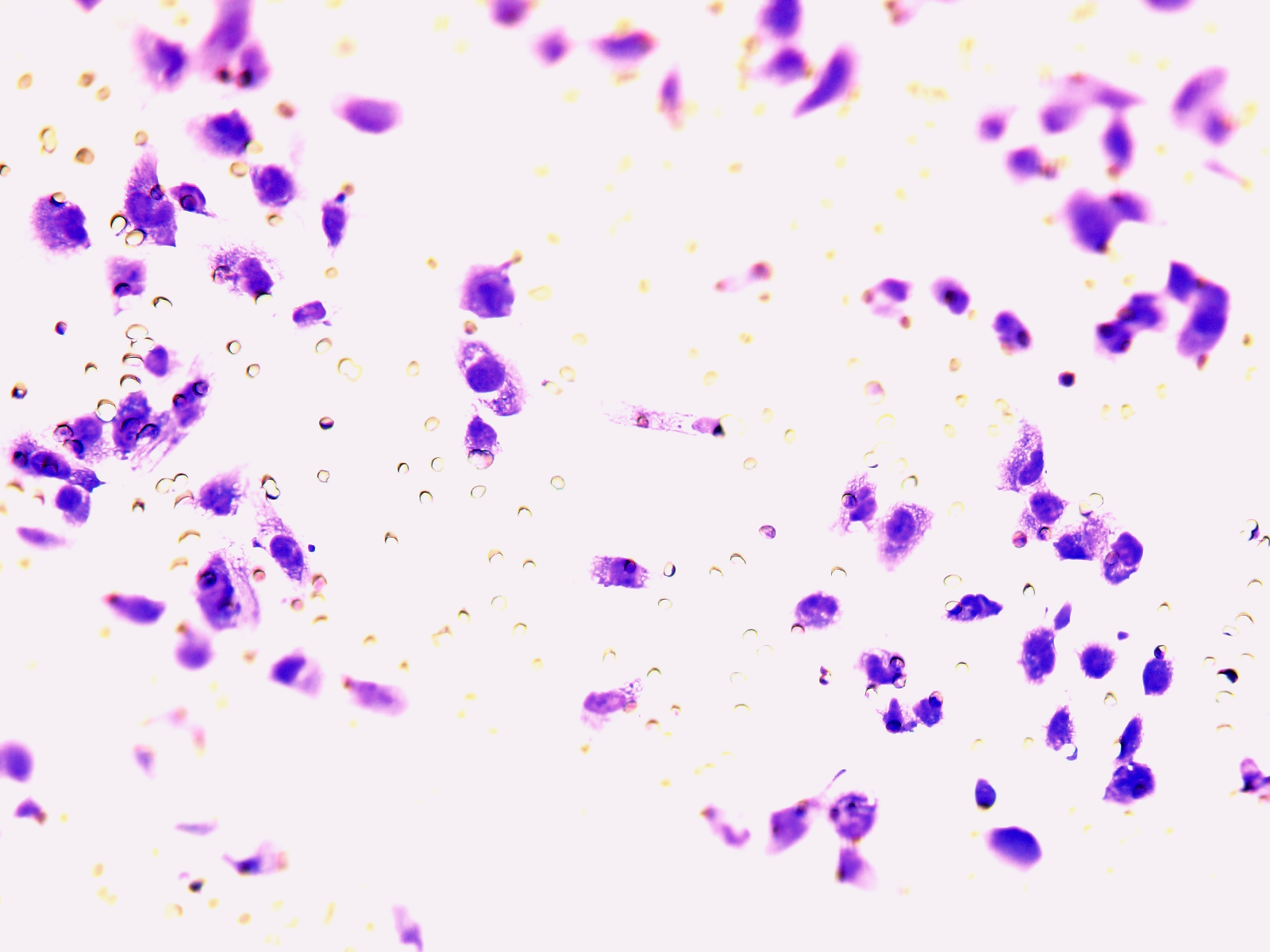

Supplement: Supplementary file 14 [file DataSheet2.ZIP › C1QTNF1 original data 2/transwell/mi-si.tif]

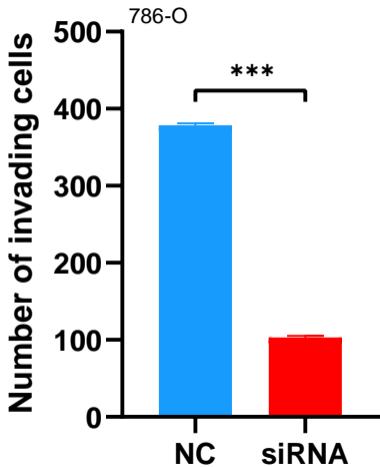

Supplement: Supplementary file 14 [file DataSheet2.ZIP › C1QTNF1 original data 2/transwell/侵袭.pdf]

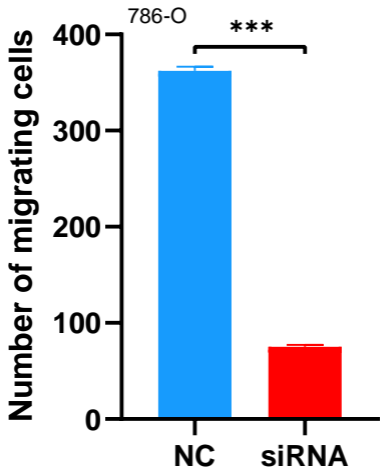

Supplement: Supplementary file 14 [file DataSheet2.ZIP › C1QTNF1 original data 2/transwell/迁移.pdf]

**
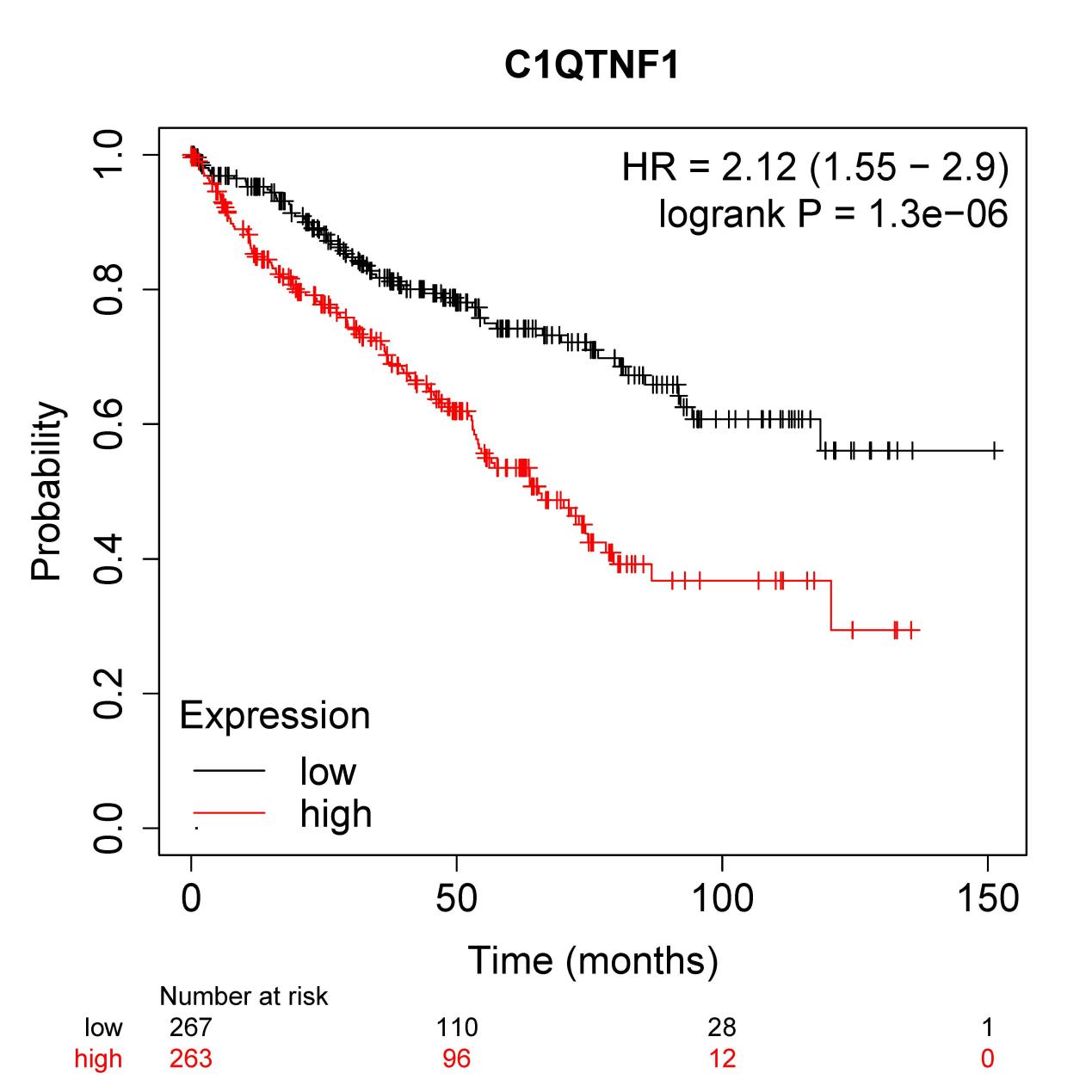
**

**Supplementary Figure 1 KIRC patients with high C1QTNF1 expression have poor survival prognosis.**

Supplement: Supplementary file 15 [file Table4.DOCX]
