# Supplementary material for: High C1QTNF1 expression mediated by potential ncRNAs is associated with poor prognosis and tumor immunity in kidney renal clear cell carcinoma
Source: Front Mol Biosci. 2023 Jul 17;10:1201155. doi: 10.3389/fmolb.2023.1201155 (PMC10387556; doi:10.3389/fmolb.2023.1201155)
Supplement: Supplementary file 5 [file Table8.DOCX]

**
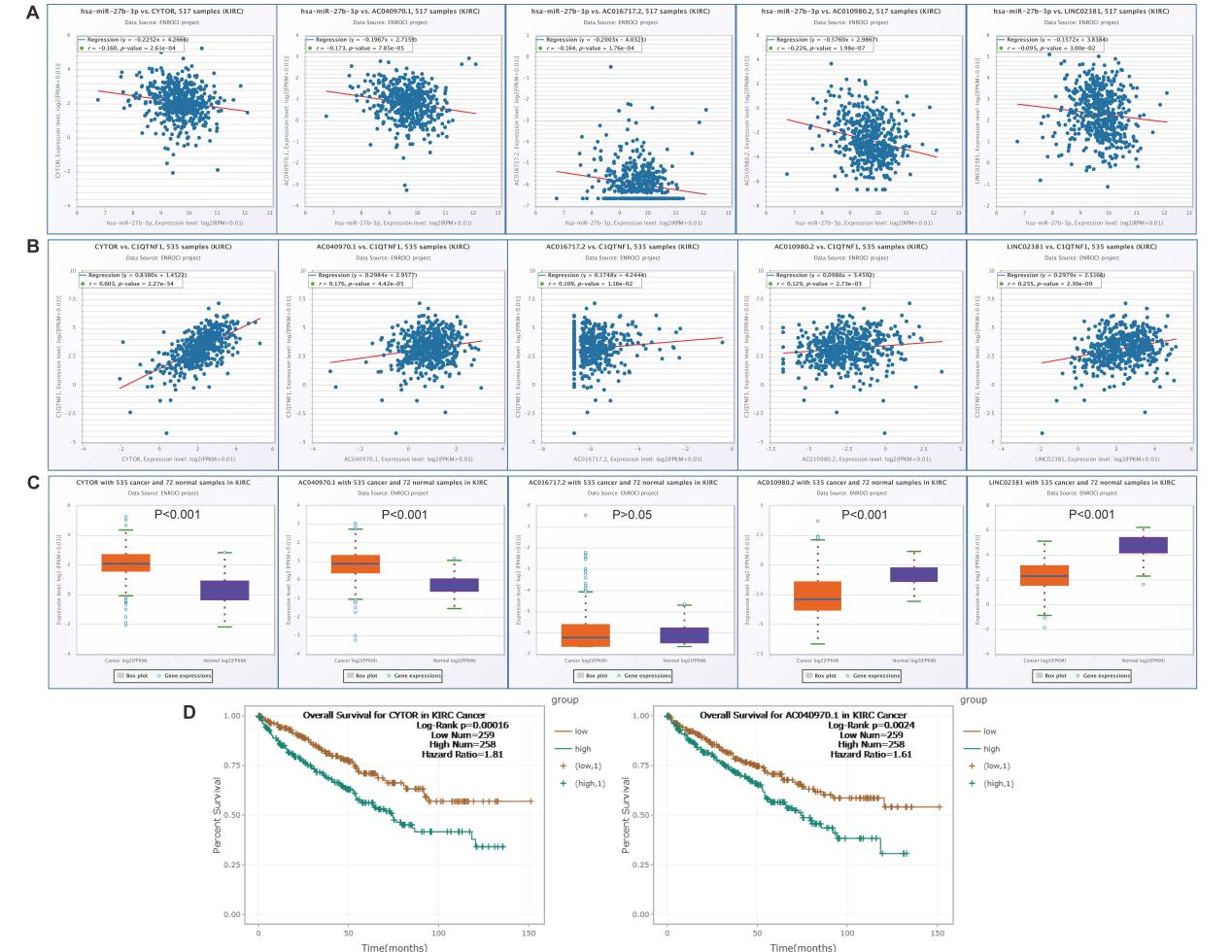
**

**Supplementary Figure 5 Identification of potential upstream lncRNAs.**

(A)The correlation between hsa-miR-27b-3p and CYTOR, AC040970.1, AC016717.2, AC010980.2,and LINC02381 in KIRC. (B) Correlation of C1QTNF1 with CYTOR, AC040970.1, AC016717.2, AC010980.2,and LINC02381 in KIRC. (C) expression of CYTOR, AC040970.1, AC016717.2, AC010980.2,and LINC02381 in KIRC and normal tissues. (D) The effect of CYTOR and AC040970.1 on the prognosis of KIRC patients.
