# Supplementary material for: High C1QTNF1 expression mediated by potential ncRNAs is associated with poor prognosis and tumor immunity in kidney renal clear cell carcinoma
Source: Front Mol Biosci. 2023 Jul 17;10:1201155. doi: 10.3389/fmolb.2023.1201155 (PMC10387556; doi:10.3389/fmolb.2023.1201155)
Supplement: Supplementary file 8 [file DataSheet1.ZIP › C1QTNF1 original data 1/Clinical relevance ananlysis/单基因Logistics回归_2022-11-11_23_58_05.docx]

| Characteristics | Total(N) | Odds Ratio(OR) | P value |
| --- | --- | --- | --- |
| T stage (T3&T4 vs. T1&T2) | 539 | 1.679 (1.176-2.405) | 0.004 |
| N stage (N1 vs. N0) | 257 | 5.427 (1.697-24.110) | 0.010 |
| M stage (M1 vs. M0) | 506 | 2.238 (1.359-3.765) | 0.002 |
| Pathologic stage (Stage III&Stage IV vs. Stage I&Stage II) | 536 | 1.637 (1.153-2.330) | 0.006 |
| Gender (Male vs. Female) | 539 | 1.352 (0.948-1.934) | 0.097 |
| Age (>60 vs. <=60) | 539 | 0.616 (0.438-0.865) | 0.005 |
