# Supplementary material for: High C1QTNF1 expression mediated by potential ncRNAs is associated with poor prognosis and tumor immunity in kidney renal clear cell carcinoma
Source: Front Mol Biosci. 2023 Jul 17;10:1201155. doi: 10.3389/fmolb.2023.1201155 (PMC10387556; doi:10.3389/fmolb.2023.1201155)
Supplement: Supplementary file 8 [file DataSheet1.ZIP › C1QTNF1 original data 1/Clinical relevance ananlysis/基线资料表_2022-11-11_23_49_54.docx]

| Characteristic | Low expression of C1QTNF1 | High expression of C1QTNF1 | p |
| --- | --- | --- | --- |
| n | 269 | 270 |  |
| T stage, n (%) |  |  | 0.002 |
| T1 | 144 (26.7%) | 134 (24.9%) |  |
| T2 | 46 (8.5%) | 25 (4.6%) |  |
| T3 | 77 (14.3%) | 102 (18.9%) |  |
| T4 | 2 (0.4%) | 9 (1.7%) |  |
| N stage, n (%) |  |  | 0.009 |
| N0 | 134 (52.1%) | 107 (41.6%) |  |
| N1 | 3 (1.2%) | 13 (5.1%) |  |
| M stage, n (%) |  |  | 0.002 |
| M0 | 226 (44.7%) | 202 (39.9%) |  |
| M1 | 26 (5.1%) | 52 (10.3%) |  |
| Pathologic stage, n (%) |  |  | 0.003 |
| Stage I | 142 (26.5%) | 130 (24.3%) |  |
| Stage II | 39 (7.3%) | 20 (3.7%) |  |
| Stage III | 58 (10.8%) | 65 (12.1%) |  |
| Stage IV | 29 (5.4%) | 53 (9.9%) |  |
| Gender, n (%) |  |  | 0.116 |
| Female | 102 (18.9%) | 84 (15.6%) |  |
| Male | 167 (31%) | 186 (34.5%) |  |
| Age, n (%) |  |  | 0.007 |
| <=60 | 118 (21.9%) | 151 (28%) |  |
| >60 | 151 (28%) | 119 (22.1%) |  |
| Age, median (IQR) | 62 (53, 72) | 59 (51, 68) | 0.002 |
