# Supplementary material for: High C1QTNF1 expression mediated by potential ncRNAs is associated with poor prognosis and tumor immunity in kidney renal clear cell carcinoma
Source: Front Mol Biosci. 2023 Jul 17;10:1201155. doi: 10.3389/fmolb.2023.1201155 (PMC10387556; doi:10.3389/fmolb.2023.1201155)
Supplement: Supplementary file 8 [file DataSheet1.ZIP › C1QTNF1 original data 1/GO and KEGG enrichment analysis/GO+KEGG-前10.docx]

| ONTOLOGY | ID | Description | GeneRatio | BgRatio | P value |
| --- | --- | --- | --- | --- | --- |
| BP | GO:0070268 | cornification | 14/322 | 112/18670 | 8.16844E-09 |
| BP | GO:0006953 | acute-phase response | 9/322 | 47/18670 | 9.24978E-08 |
| BP | GO:0031424 | keratinization | 17/322 | 224/18670 | 3.75682E-07 |
| BP | GO:0006720 | isoprenoid metabolic process | 13/322 | 139/18670 | 8.70814E-07 |
| BP | GO:0006721 | terpenoid metabolic process | 12/322 | 120/18670 | 1.12715E-06 |
| BP | GO:0043062 | extracellular structure organization | 23/322 | 422/18670 | 1.29364E-06 |
| BP | GO:0055067 | monovalent inorganic cation homeostasis | 13/322 | 154/18670 | 2.77519E-06 |
| BP | GO:0098656 | anion transmembrane transport | 18/322 | 288/18670 | 2.86174E-06 |
| BP | GO:0042445 | hormone metabolic process | 16/322 | 232/18670 | 2.91929E-06 |
| BP | GO:0016101 | diterpenoid metabolic process | 11/322 | 110/18670 | 3.16297E-06 |
| CC | GO:0062023 | collagen-containing extracellular matrix | 27/340 | 406/19717 | 2.45826E-09 |
| CC | GO:0045177 | apical part of cell | 20/340 | 384/19717 | 1.27061E-05 |
| CC | GO:0005796 | Golgi lumen | 9/340 | 102/19717 | 6.79779E-05 |
| CC | GO:0034364 | high-density lipoprotein particle | 5/340 | 26/19717 | 7.22903E-05 |
| CC | GO:0005788 | endoplasmic reticulum lumen | 16/340 | 309/19717 | 0.00010012 |
| CC | GO:0016323 | basolateral plasma membrane | 13/340 | 217/19717 | 0.000106688 |
| CC | GO:0016324 | apical plasma membrane | 16/340 | 318/19717 | 0.000139454 |
| CC | GO:0016471 | vacuolar proton-transporting V-type ATPase complex | 4/340 | 17/19717 | 0.000173105 |
| CC | GO:0005882 | intermediate filament | 12/340 | 214/19717 | 0.000358123 |
| CC | GO:0034358 | plasma lipoprotein particle | 5/340 | 37/19717 | 0.000410123 |
| MF | GO:0004252 | serine-type endopeptidase activity | 14/304 | 160/17697 | 7.02317E-07 |
| MF | GO:0048018 | receptor ligand activity | 25/304 | 482/17697 | 9.93195E-07 |
| MF | GO:0008509 | anion transmembrane transporter activity | 20/304 | 327/17697 | 1.0333E-06 |
| MF | GO:0008201 | heparin binding | 14/304 | 169/17697 | 1.35878E-06 |
| MF | GO:0008236 | serine-type peptidase activity | 14/304 | 182/17697 | 3.27091E-06 |
| MF | GO:0017171 | serine hydrolase activity | 14/304 | 186/17697 | 4.21794E-06 |
| MF | GO:0005201 | extracellular matrix structural constituent | 13/304 | 163/17697 | 4.94288E-06 |
| MF | GO:0015103 | inorganic anion transmembrane transporter activity | 12/304 | 146/17697 | 8.41712E-06 |
| MF | GO:0005200 | structural constituent of cytoskeleton | 10/304 | 102/17697 | 1.01926E-05 |
| MF | GO:0005539 | glycosaminoglycan binding | 15/304 | 229/17697 | 1.04661E-05 |
| KEGG | hsa00830 | Retinol metabolism | 9/151 | 68/8076 | 4.23273E-06 |
| KEGG | hsa04966 | Collecting duct acid secretion | 6/151 | 27/8076 | 8.27733E-06 |
| KEGG | hsa00140 | Steroid hormone biosynthesis | 7/151 | 61/8076 | 0.00013017 |
| KEGG | hsa04610 | Complement and coagulation cascades | 8/151 | 85/8076 | 0.000176726 |
| KEGG | hsa00982 | Drug metabolism - cytochrome P450 | 7/151 | 71/8076 | 0.00033954 |
| KEGG | hsa00980 | Metabolism of xenobiotics by cytochrome P450 | 7/151 | 77/8076 | 0.000559259 |
| KEGG | hsa04721 | Synaptic vesicle cycle | 7/151 | 78/8076 | 0.000604919 |
| KEGG | hsa05204 | Chemical carcinogenesis | 7/151 | 82/8076 | 0.000818032 |
| KEGG | hsa05323 | Rheumatoid arthritis | 7/151 | 93/8076 | 0.001718651 |
| KEGG | hsa00591 | Linoleic acid metabolism | 4/151 | 29/8076 | 0.001938271 |
