# Supplementary material for: High C1QTNF1 expression mediated by potential ncRNAs is associated with poor prognosis and tumor immunity in kidney renal clear cell carcinoma
Source: Front Mol Biosci. 2023 Jul 17;10:1201155. doi: 10.3389/fmolb.2023.1201155 (PMC10387556; doi:10.3389/fmolb.2023.1201155)

Characteristics	N (%)	HR (95% CI)	P value
T stage			
T1&T2	349 (64.8)	2.12(1.33−3.39)	0.002
T3&T4	190 (35.2)	1.54(1.05−2.28)	0.027
N stage			
N0	241 (93.8)	1.85(1.21−2.85)	0.005
N1	16 (6.2)	0.95(0.29−3.12)	0.932
M stage			
M0	428 (84.6)	1.66(1.13−2.42)	0.009
M1	78 (15.4)	0.94(0.58−1.55)	0.819
Pathologic stage			
Stage I&Stage II	331 (61.8)	2.68(1.59−4.52)	<0.001
Stage III&Stage IV	205 (38.2)	1.38(0.96−2.00)	0.08

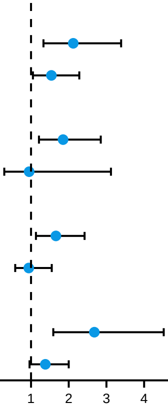

Supplement: Supplementary file 8 [file DataSheet1.ZIP › C1QTNF1 original data 1/Subgroup survival prognosis analysis/森林图_2022-11-16_10_34_28.pdf]
