# Supplementary material for: High C1QTNF1 expression mediated by potential ncRNAs is associated with poor prognosis and tumor immunity in kidney renal clear cell carcinoma
Source: Front Mol Biosci. 2023 Jul 17;10:1201155. doi: 10.3389/fmolb.2023.1201155 (PMC10387556; doi:10.3389/fmolb.2023.1201155)
Supplement: Supplementary file 9 [file Table7.DOCX]

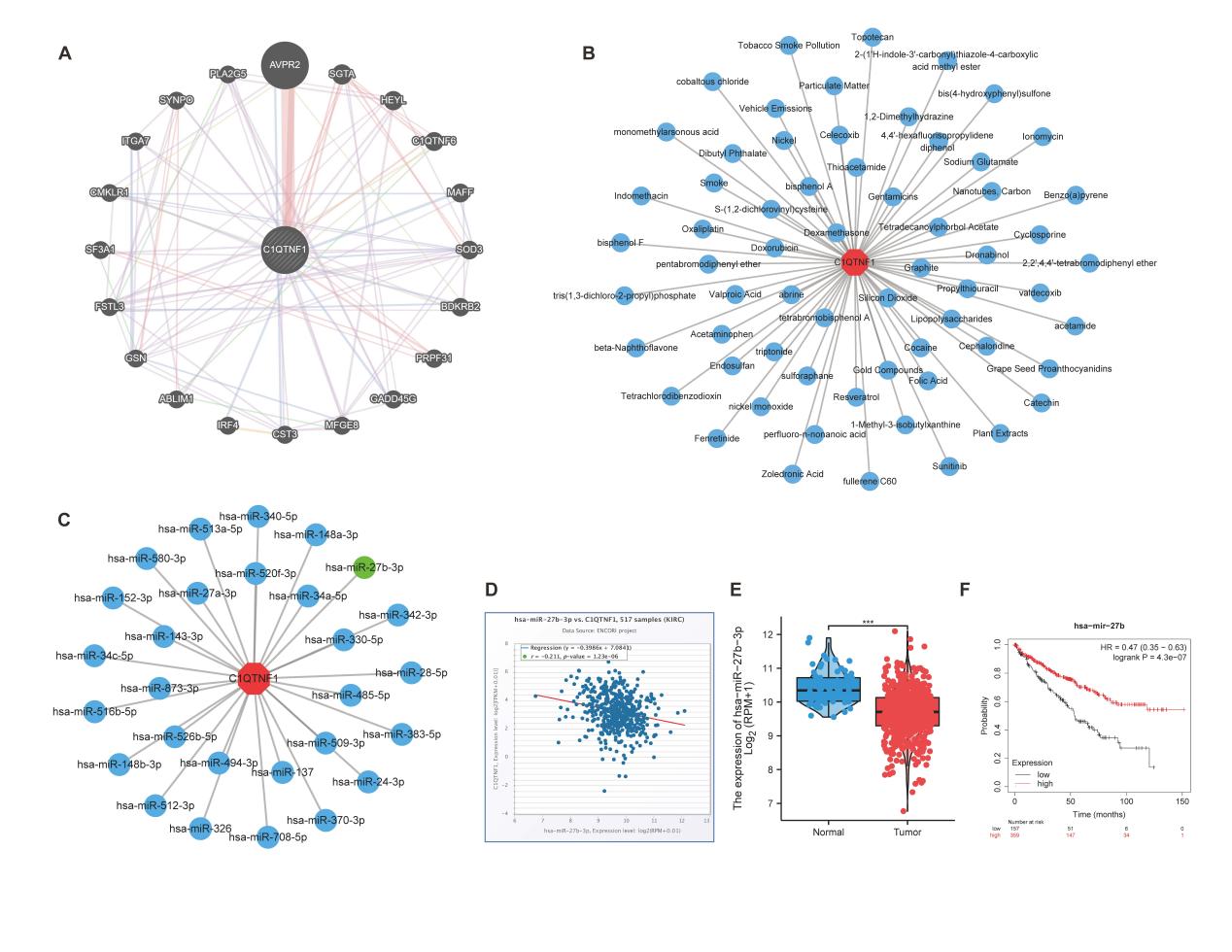


**Supplementary Figure 4 Construction of gene interaction networks as well as potential upstream miRNAs.**

(A) The interaction network of C1QTNF1 and related genes was constructed based on the GeneMANIA website. (B) C1QTNF1 chemo drug network was constructed based on the CDC database. (C) miRNA-CQTNF1 regulatory network constructed based on the ENCORI database. (D) Correlation analysis between C1QTNF1 expression and hsa-miR-27b-3p. (E) Expression analysis of hsa-miR-27b-3p in KIRC and adjacent normal tissues in TCGA database. (F) Effect of hsa-miR-27b-3p on the prognosis of KIRC patients.
