# Supplementary material for: High C1QTNF1 expression mediated by potential ncRNAs is associated with poor prognosis and tumor immunity in kidney renal clear cell carcinoma
Source: Front Mol Biosci. 2023 Jul 17;10:1201155. doi: 10.3389/fmolb.2023.1201155 (PMC10387556; doi:10.3389/fmolb.2023.1201155)
Supplement: Supplementary file 13 [file Table5.DOCX]

**
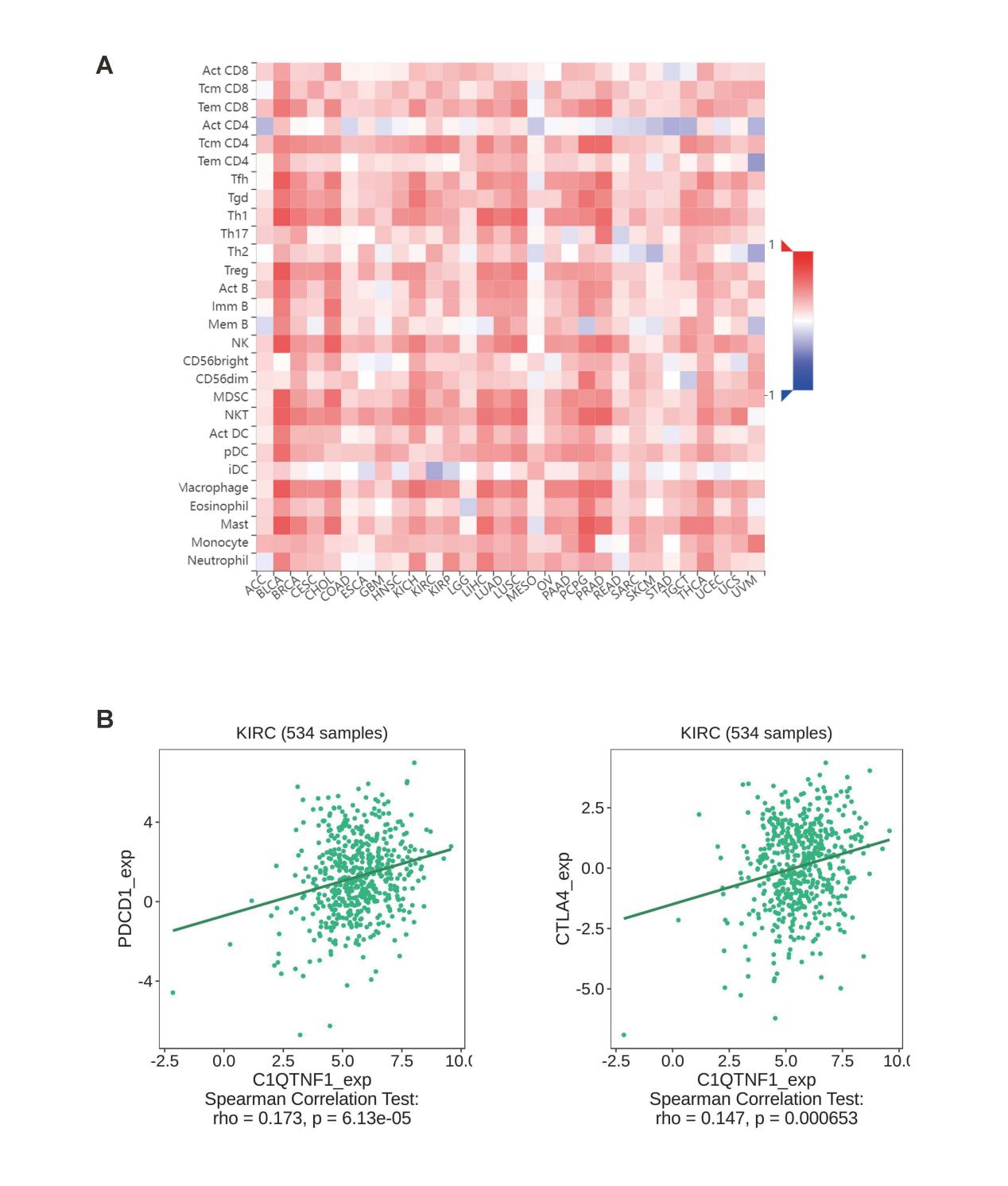
**

**Supplementary Figure 2 Relationship between C1QTNF1 and immune infiltration in TISIDB database.**

**(A) C1QTNF1 was strongly correlated with TIL infiltration abundance. (B) In the TISIDB database, C1QTNF1 was also positively correlated with two immune markers. P < 0.05.**
