# Supplementary material for: High C1QTNF1 expression mediated by potential ncRNAs is associated with poor prognosis and tumor immunity in kidney renal clear cell carcinoma
Source: Front Mol Biosci. 2023 Jul 17;10:1201155. doi: 10.3389/fmolb.2023.1201155 (PMC10387556; doi:10.3389/fmolb.2023.1201155)
Supplement: Supplementary file 16 [file Table6.DOCX]

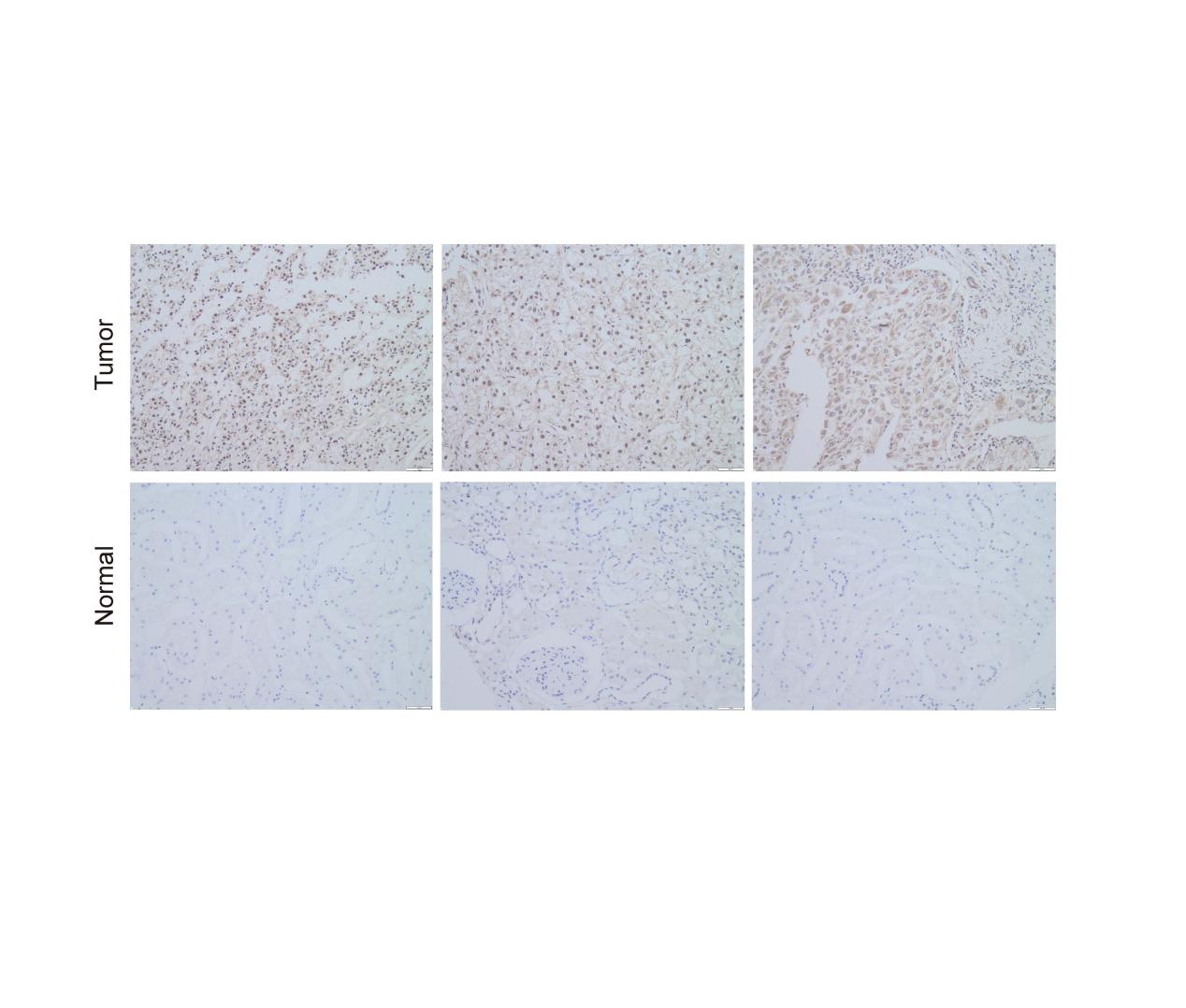


**Supplementary Figure 3**

The results of IHC staining showed that the protein level of C1QTNF1 was increased in KIRC tissues compared with normal tissues.
