# Supplementary material for: High C1QTNF1 expression mediated by potential ncRNAs is associated with poor prognosis and tumor immunity in kidney renal clear cell carcinoma
Source: Front Mol Biosci. 2023 Jul 17;10:1201155. doi: 10.3389/fmolb.2023.1201155 (PMC10387556; doi:10.3389/fmolb.2023.1201155)
Supplement: Supplementary file 8 [file DataSheet1.ZIP › C1QTNF1 original data 1/Time ROC curve analysis/时间依赖ROC_2022-11-11_17_04_50.pdf]

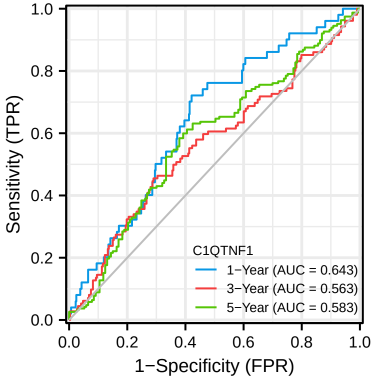

Sensitivity (TPR)
1.0
0.8
0.6
0.4
0.2
0.0
0.0
0.2
0.4
0.6
0.8
1.0
1−Specificity (FPR)
C1QTNF1
1−Year (AUC = 0.643)
3−Year (AUC = 0.563)
5−Year (AUC = 0.583)
