# Supplementary material for: High C1QTNF1 expression mediated by potential ncRNAs is associated with poor prognosis and tumor immunity in kidney renal clear cell carcinoma
Source: Front Mol Biosci. 2023 Jul 17;10:1201155. doi: 10.3389/fmolb.2023.1201155 (PMC10387556; doi:10.3389/fmolb.2023.1201155)
Supplement: Supplementary file 8 [file DataSheet1.ZIP › C1QTNF1 original data 1/Univariate and multivariate Cox/Calibration可视化_2022-11-12_00_17_03.pdf]

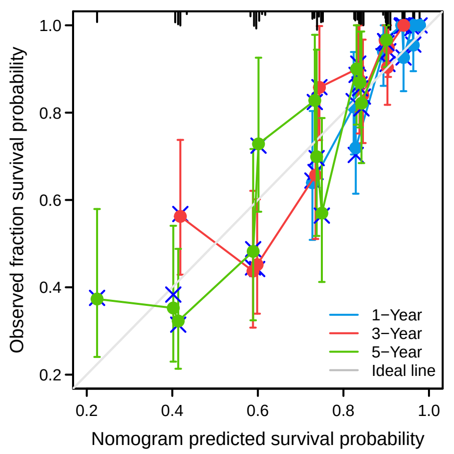

1.0
0.8
0.6
0.4
0.2
Observed fraction survival probability
0.2
0.4
0.6
0.8
1.0
Nomogram predicted survival probability
1−Year
3−Year
5−Year
Ideal line
