# Supplementary material for: High C1QTNF1 expression mediated by potential ncRNAs is associated with poor prognosis and tumor immunity in kidney renal clear cell carcinoma
Source: Front Mol Biosci. 2023 Jul 17;10:1201155. doi: 10.3389/fmolb.2023.1201155 (PMC10387556; doi:10.3389/fmolb.2023.1201155)
Supplement: Supplementary file 8 [file DataSheet1.ZIP › C1QTNF1 original data 1/Univariate and multivariate Cox/Nomogram图_2022-11-12_00_12_35.pdf]

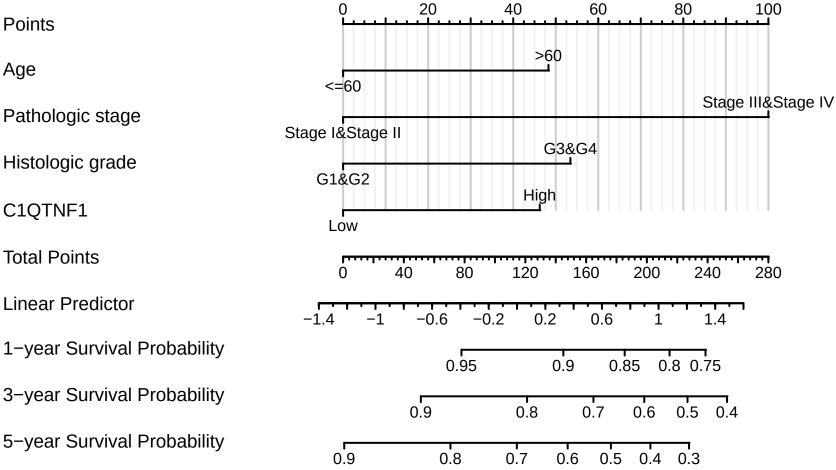
Points
0
20
40
60
80
100
Age
>60
<=60
Pathologic stage
Stage III&Stage IV
Stage I&Stage II
Histologic grade
G3&G4
G1&G2
C1QTNF1
High
Low
Total Points
0
40
80
120
160
200
240
280
Linear Predictor
−1.4
−1
−0.6
−0.2
0.2
0.6
1
1.4
1−year Survival Probability
0.95
0.9
0.85
0.8
0.75
3−year Survival Probability
0.9
0.8
0.7
0.6
0.5
0.4
5−year Survival Probability
0.9
0.8
0.7
0.6
0.5
0.4
0.3
