# Supplementary material for: High C1QTNF1 expression mediated by potential ncRNAs is associated with poor prognosis and tumor immunity in kidney renal clear cell carcinoma
Source: Front Mol Biosci. 2023 Jul 17;10:1201155. doi: 10.3389/fmolb.2023.1201155 (PMC10387556; doi:10.3389/fmolb.2023.1201155)
Supplement: Supplementary file 8 [file DataSheet1.ZIP › C1QTNF1 original data 1/Univariate and multivariate Cox/单_多因素Cox回归_2022-11-12_00_05_30.docx]

| Characteristics | Total(N) | Univariate analysis | |  | Multivariate analysis | |
| --- | --- | --- | --- | --- | --- | --- |
|  |  | Hazard ratio (95% CI) | P value |  | Hazard ratio (95% CI) | P value |
| T stage | 539 |  |  |  |  |  |
| T1&T2 | 349 | Reference |  |  |  |  |
| T3&T4 | 190 | 3.228 (2.382-4.374) | **<0.001** |  | 0.975 (0.424-2.246) | 0.953 |
| N stage | 257 |  |  |  |  |  |
| N0 | 241 | Reference |  |  |  |  |
| N1 | 16 | 3.453 (1.832-6.508) | **<0.001** |  | 1.705 (0.831-3.496) | 0.146 |
| Pathologic stage | 536 |  |  |  |  |  |
| Stage I&Stage II | 331 | Reference |  |  |  |  |
| Stage III&Stage IV | 205 | 3.946 (2.872-5.423) | **<0.001** |  | 2.567 (1.074-6.133) | **0.034** |
| Gender | 539 |  |  |  |  |  |
| Female | 186 | Reference |  |  |  |  |
| Male | 353 | 0.930 (0.682-1.268) | 0.648 |  |  |  |
| Age | 539 |  |  |  |  |  |
| <=60 | 269 | Reference |  |  |  |  |
| >60 | 270 | 1.765 (1.298-2.398) | **<0.001** |  | 1.638 (1.069-2.510) | **0.023** |
| Race | 532 |  |  |  |  |  |
| White | 467 | Reference |  |  |  |  |
| Black or African American | 57 | 0.857 (0.465-1.582) | 0.622 |  |  |  |
| Asian | 8 | 0.545 (0.076-3.905) | 0.546 |  |  |  |
| C1QTNF1 | 539 |  |  |  |  |  |
| Low | 269 | Reference |  |  |  |  |
| High | 270 | 1.819 (1.338-2.472) | **<0.001** |  | 1.610 (1.040-2.491) | **0.033** |
| Histologic grade | 531 |  |  |  |  |  |
| G1&G2 | 249 | Reference |  |  |  |  |
| G3&G4 | 282 | 2.702 (1.918-3.807) | **<0.001** |  | 1.871 (1.145-3.058) | **0.012** |
